# Supplementary material for: High performance mechano-optoelectronic molecular switch
Source: Nat Commun. 2023 Sep 13;14:5639. doi: 10.1038/s41467-023-41433-0 (PMC10499996; doi:10.1038/s41467-023-41433-0)
Supplement: Supplementary file 1 — Supplementary Information [file 41467_2023_41433_MOESM1_ESM.docx]

**Supplementary Information for**

**High Performance Mechano-optoelectronic Molecular Switch**

Zhenyu Yang^1,4^, Pierre-André Cazade^2,4^, Jin-Liang Lin^1,4^, Zhou Cao^1^, Ningyue Chen^1^, Dongdong Zhang^1^, Lian Duan^1^, Christian A. Nijhuis^3^, Damien Thompson^2✉^ & Yuan Li^1✉^

^1^ Key Laboratory of Organic Optoelectronics and Molecular Engineering and Laboratory of Flexible Electronics Technology, Department of Chemistry, Tsinghua University, Beijing 100084, China.

^2^ Department of Physics, Bernal Institute, University of Limerick, Limerick, V94 T9PX, Ireland.

^3^ Department of Molecules and Materials MESA+ Institute for Nanotechnology, Molecules Center and Center for Brain-Inspired NanoSystems Faculty of Science and Technology, University of Twente; Enschede, The Netherlands.

^4^ These authors contributed equally: Zhenyu Yang, Pierre-André Cazade, and Jin-Liang Lin

^✉^Corresponding author. Email: [damien.thompson@ul.ie](mailto:damien.thompson@ul.ie) and [yuanli_thu@tsinghua.edu.cn](mailto:yuanli_thu@tsinghua.edu.cn)

This file includes:

Supplementary Figures: page 2 to 56

Supplementary Tables: page 57

Supplementary Discussion: page 58 to 70

Supplementary References: page 70

**Supplementary Figures**

**Supplementary Fig. 1**.**The synthetic route of target molecule TPE-C_10_-SH.**

**TPE-OH**

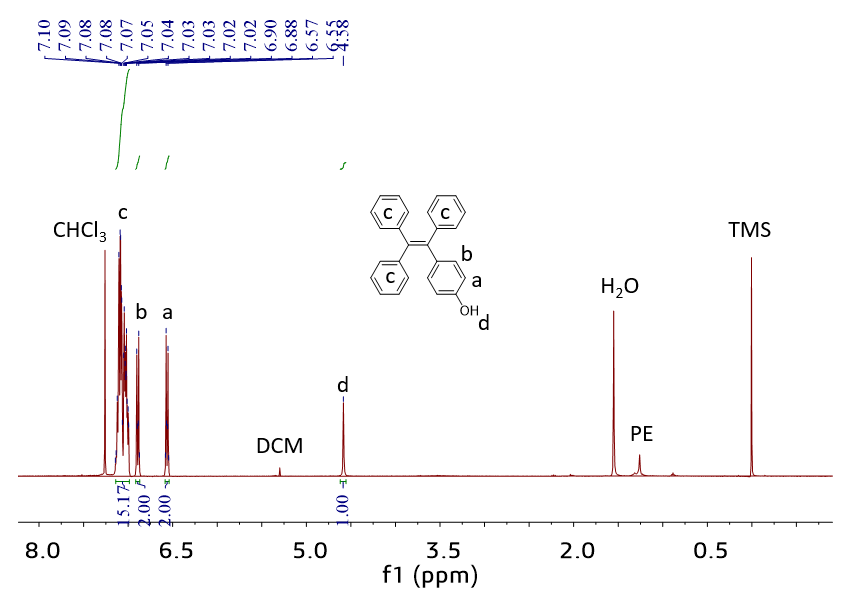


**Supplementary Fig. 2.** **The ^1^H-NMR spectrum of TPE-OH.**


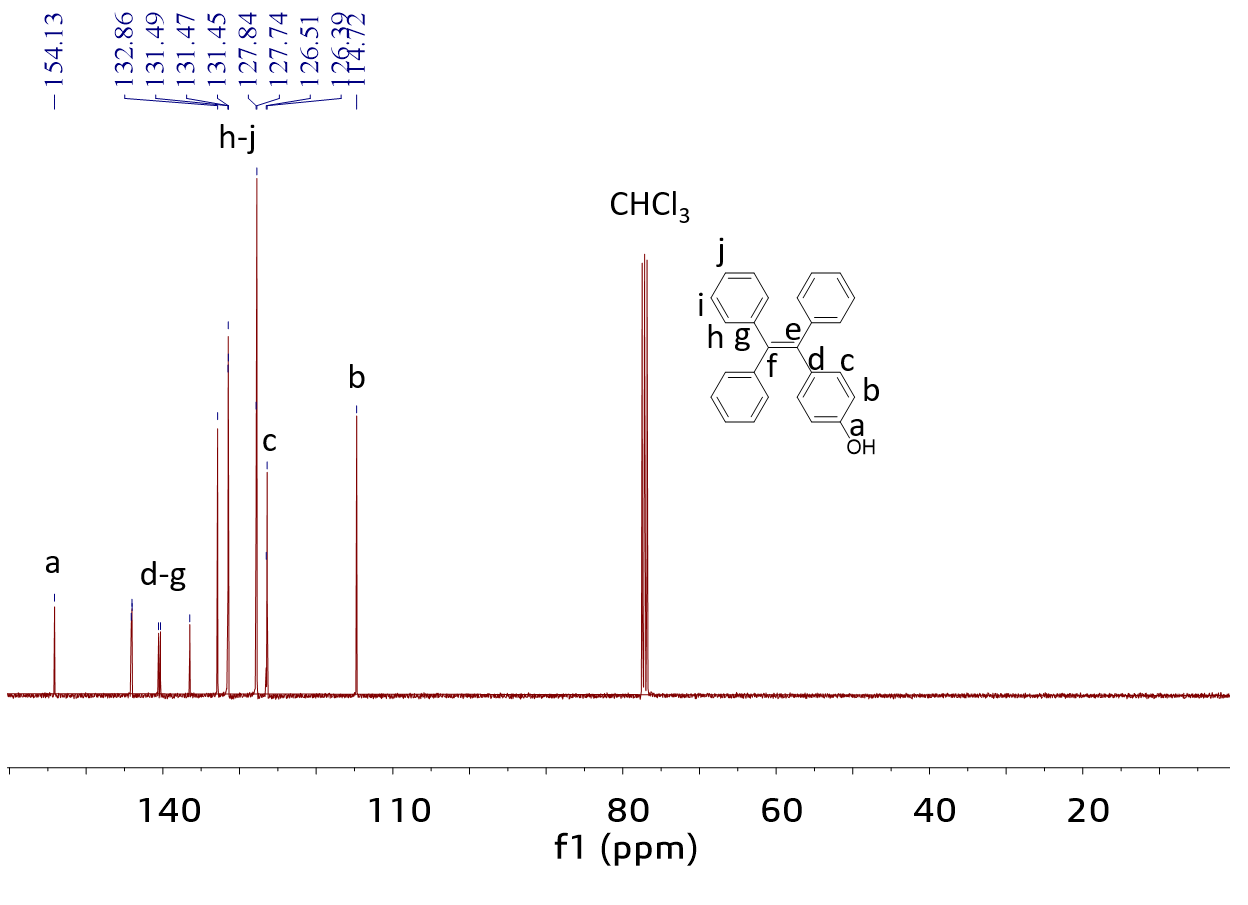


**Supplementary Fig. 3**. **The ^13^C-NMR spectrum of TPE-OH.**


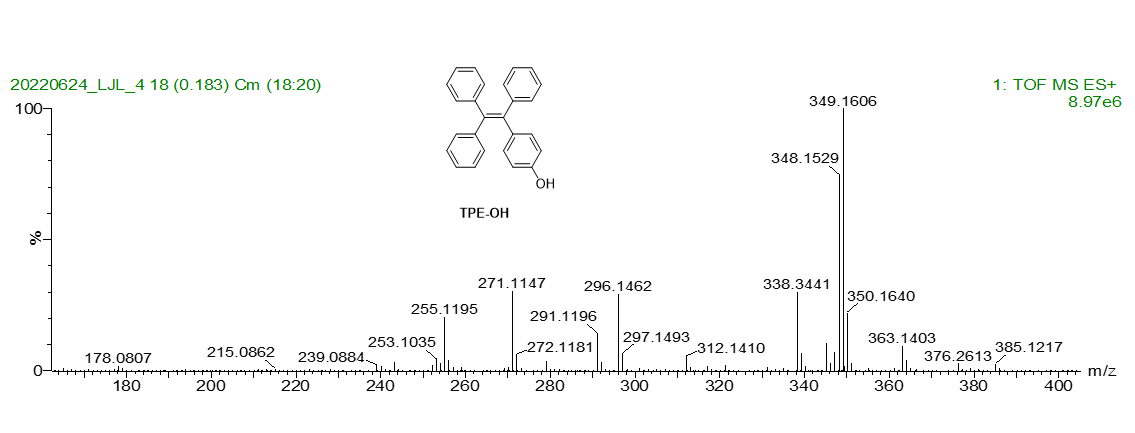


**Supplementary Fig. 4.** **The HRMS spectrum of TPE-OH.**

**TPE-C_10_-Br**

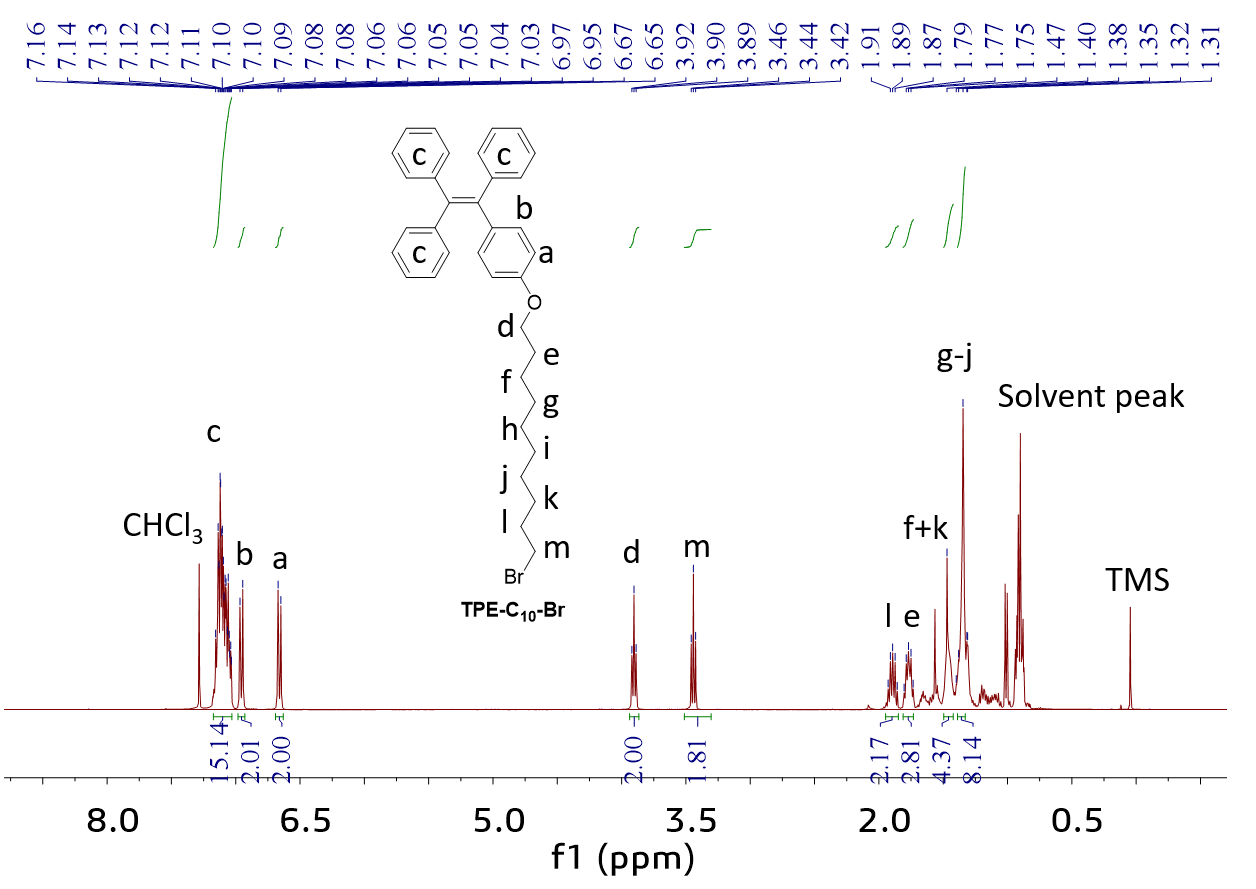


**Supplementary Fig. 5.** **The ^1^H-NMR spectrum of TPE-C_10_-Br.**


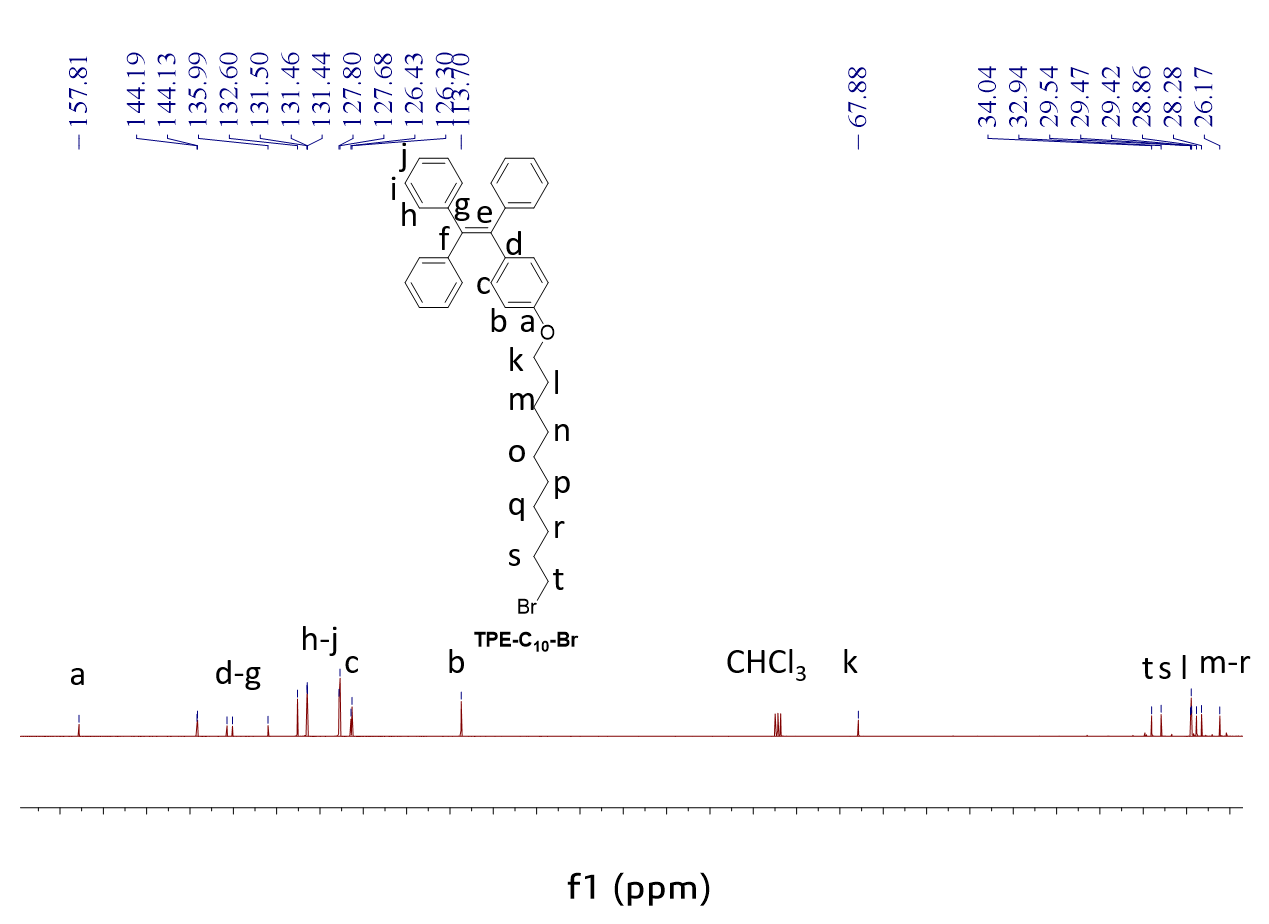


**Supplementary Fig. 6. The ^13^C-NMR spectrum of TPE-C_10_-Br.**


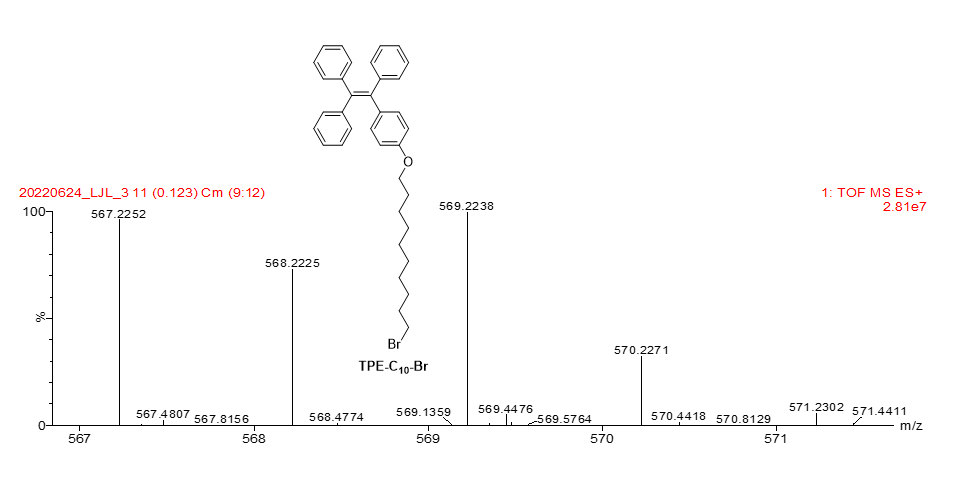


**Supplementary Fig. 7. The HRMS spectrum of TPE-C_10_-Br.**

**TPE-C_10_-SAc**

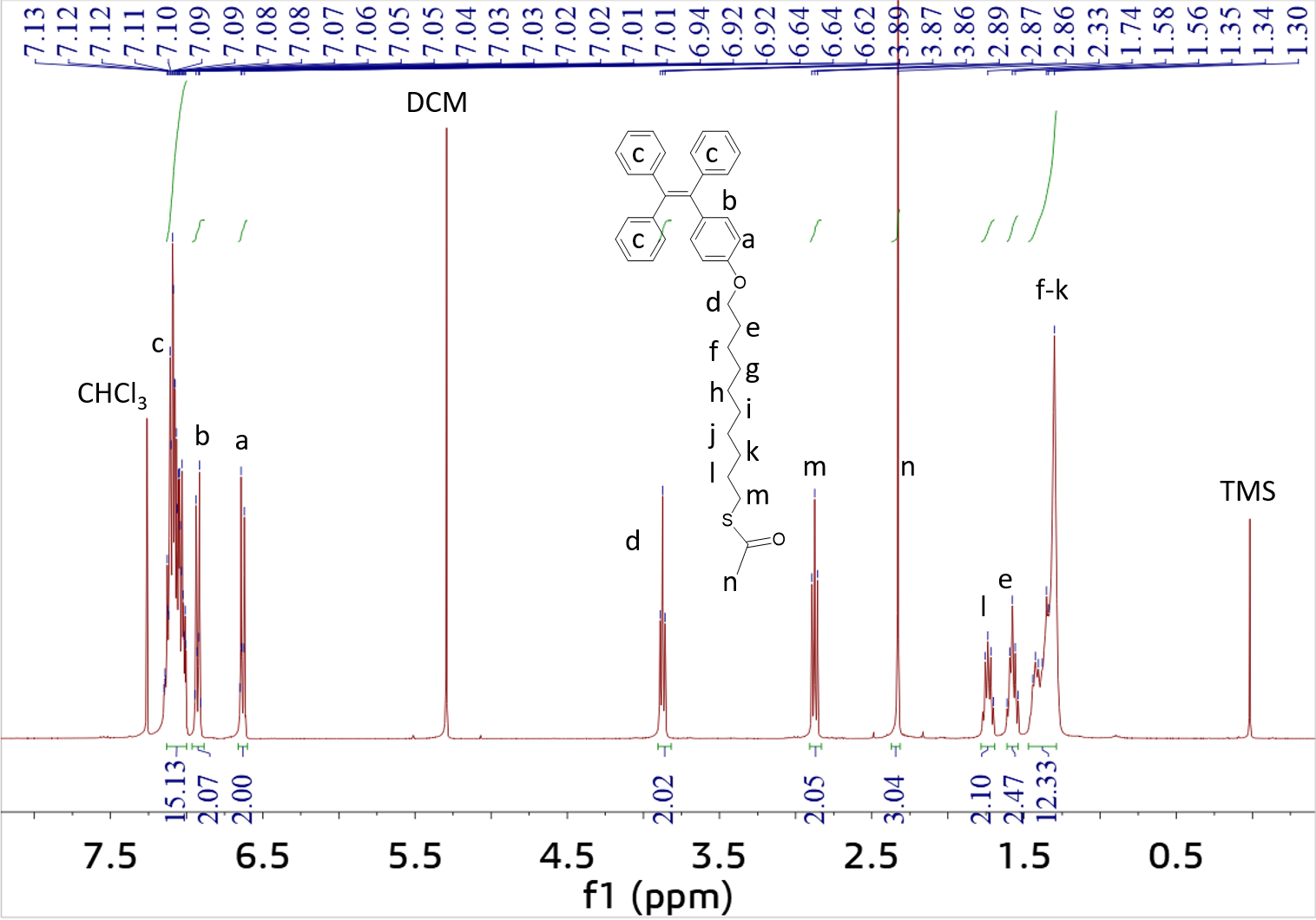


**Supplementary Fig. 8.** **The ^1^H-NMR spectrum of TPE-C_10_-SAc.**


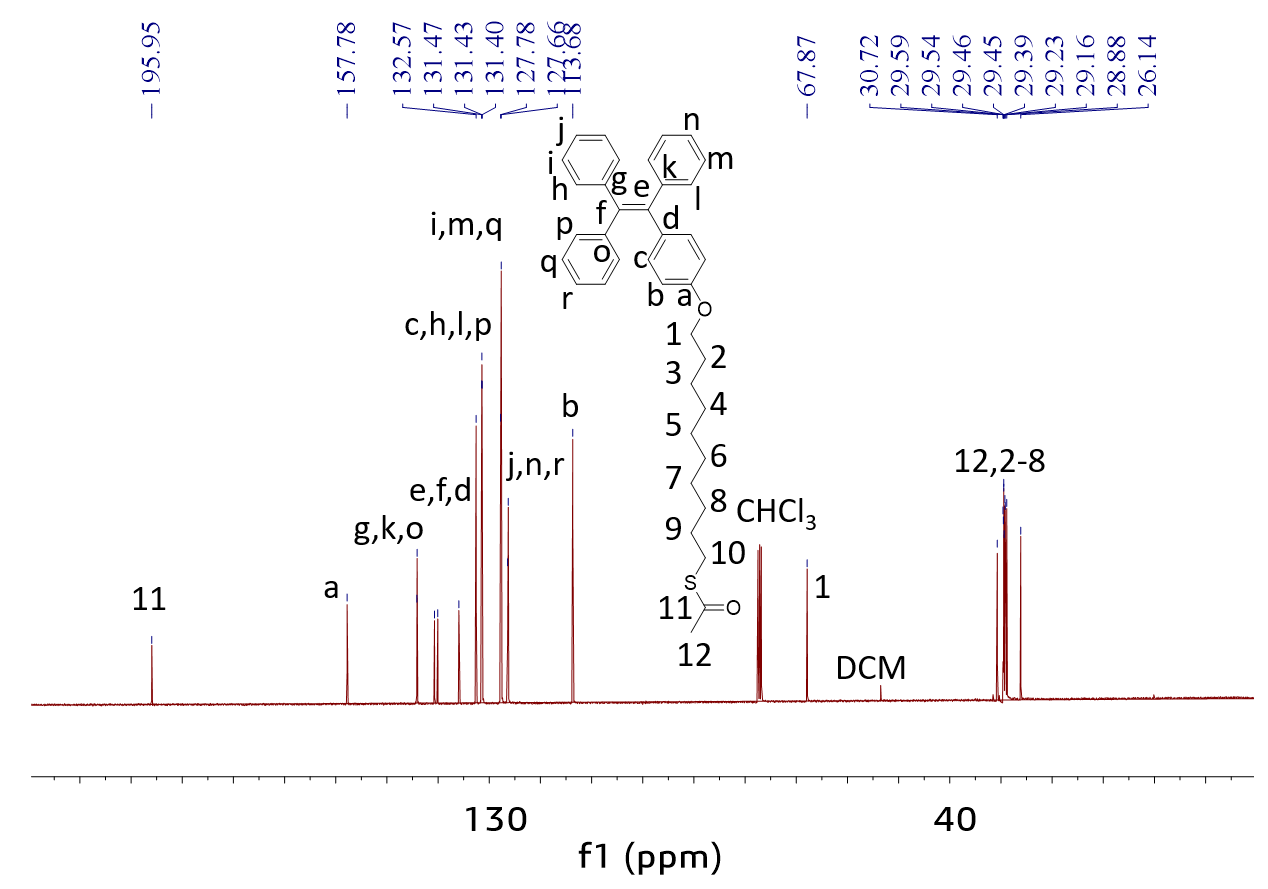


**Supplementary Fig. 9.** **The ^13^C-NMR spectrum of TPE-C_10_-SAc.**


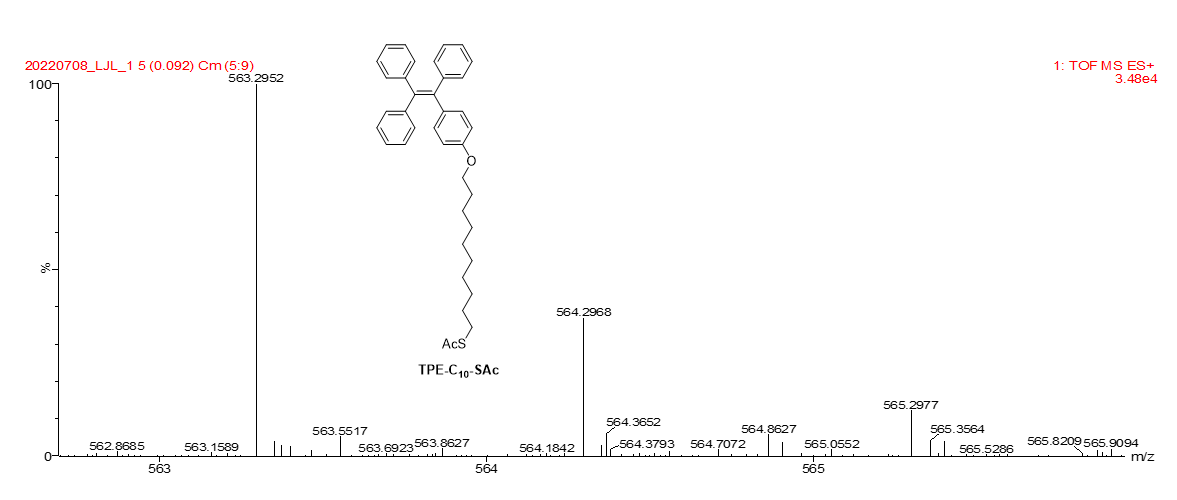


**Supplementary Fig. 10. The HRMS spectrum of TPE-C_10_-SAc.**

**TPE-C_10_-SH**

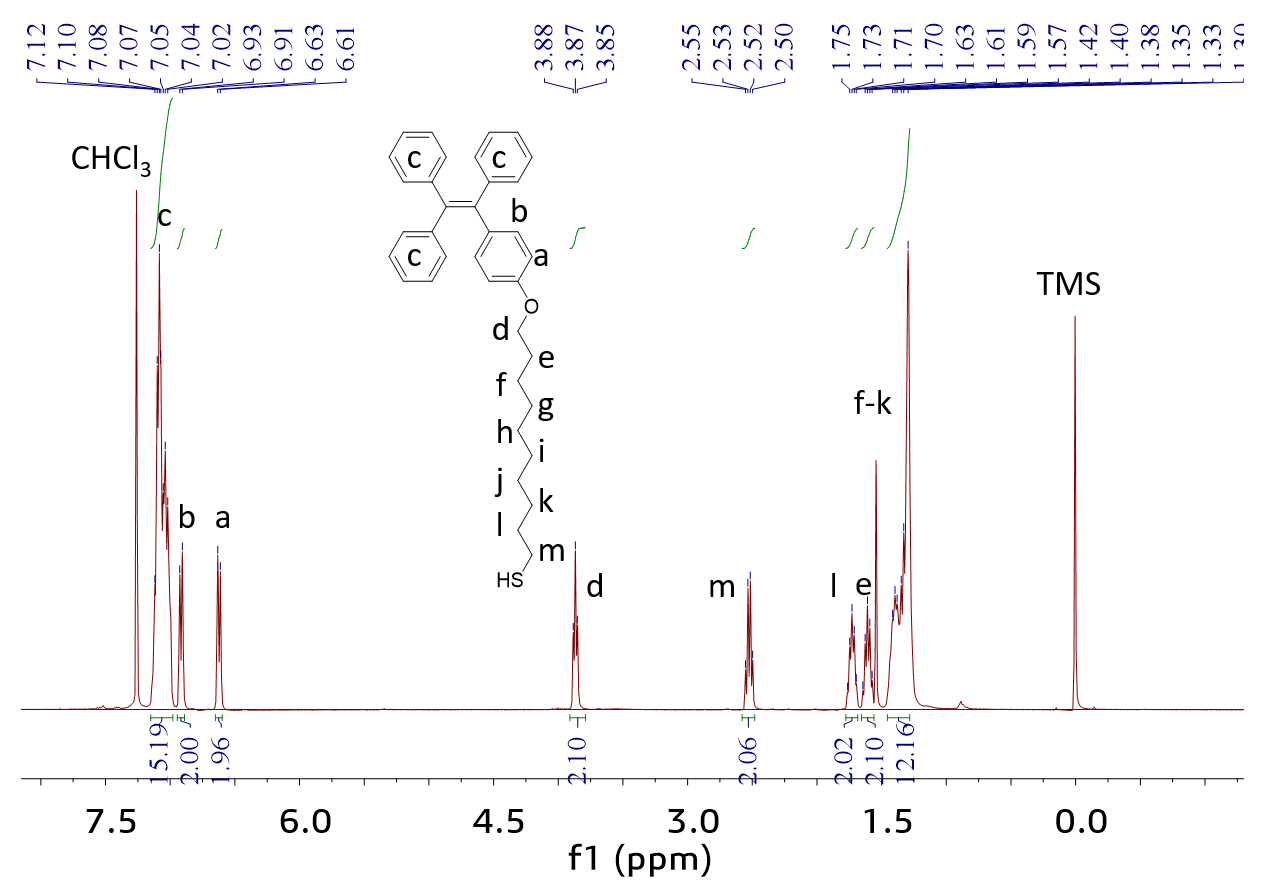


**Supplementary Fig. 11.** **The ^1^H-NMR spectrum of TPE-C_10_-SH.**


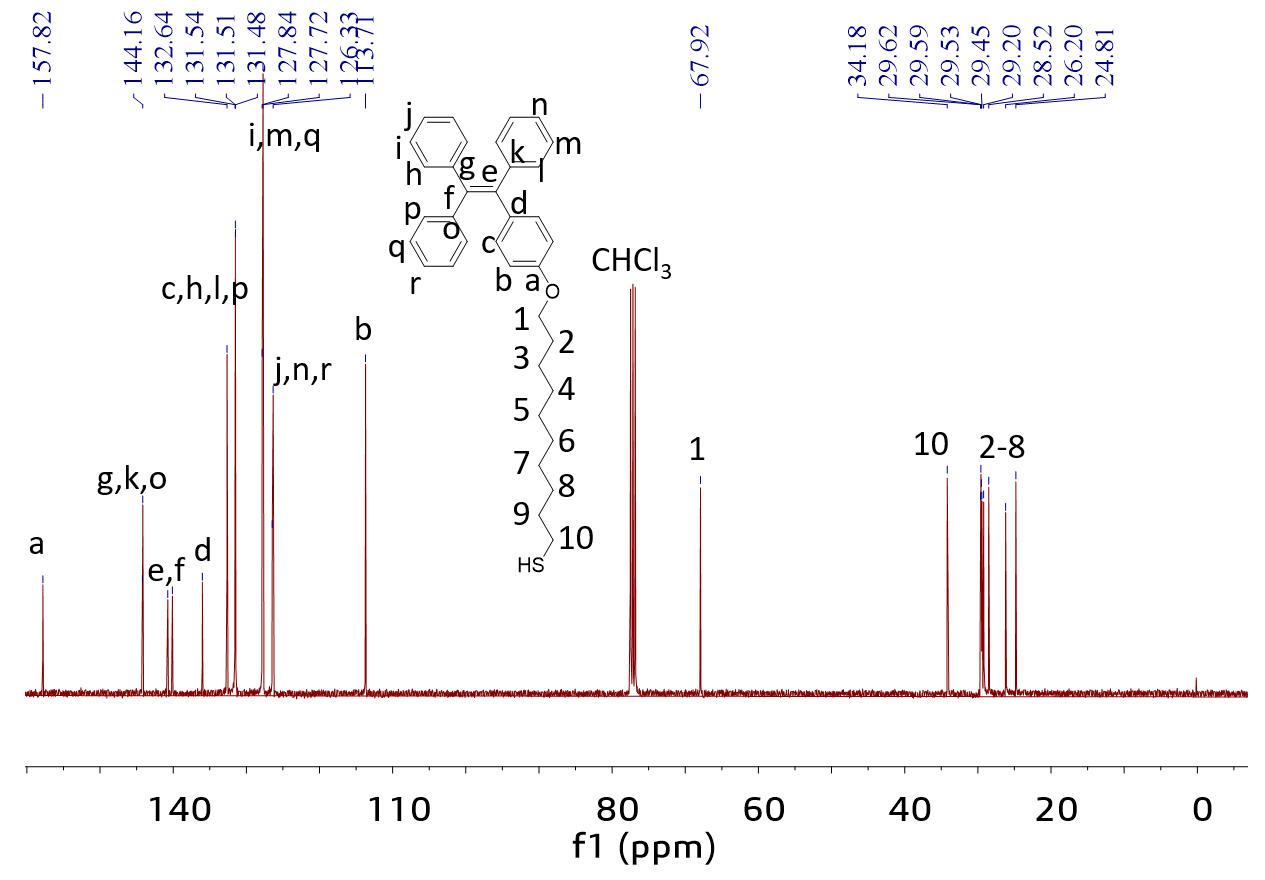


**Supplementary Fig. 12. The ^13^C-NMR spectrum of TPE-C_10_-SH.**


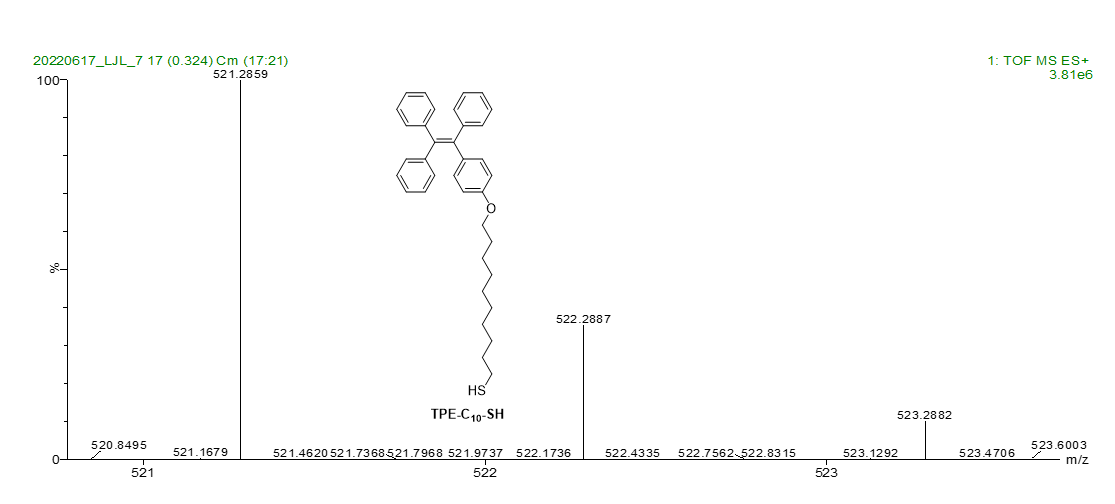


**Supplementary Fig. 13.** **The HRMS spectrum of TPE-C_10_-SH.**

**di-TPE**

**Supplementary Fig. 14**.**The synthetic route of di-TPE.**

**2Ph-2Bpin-NH**

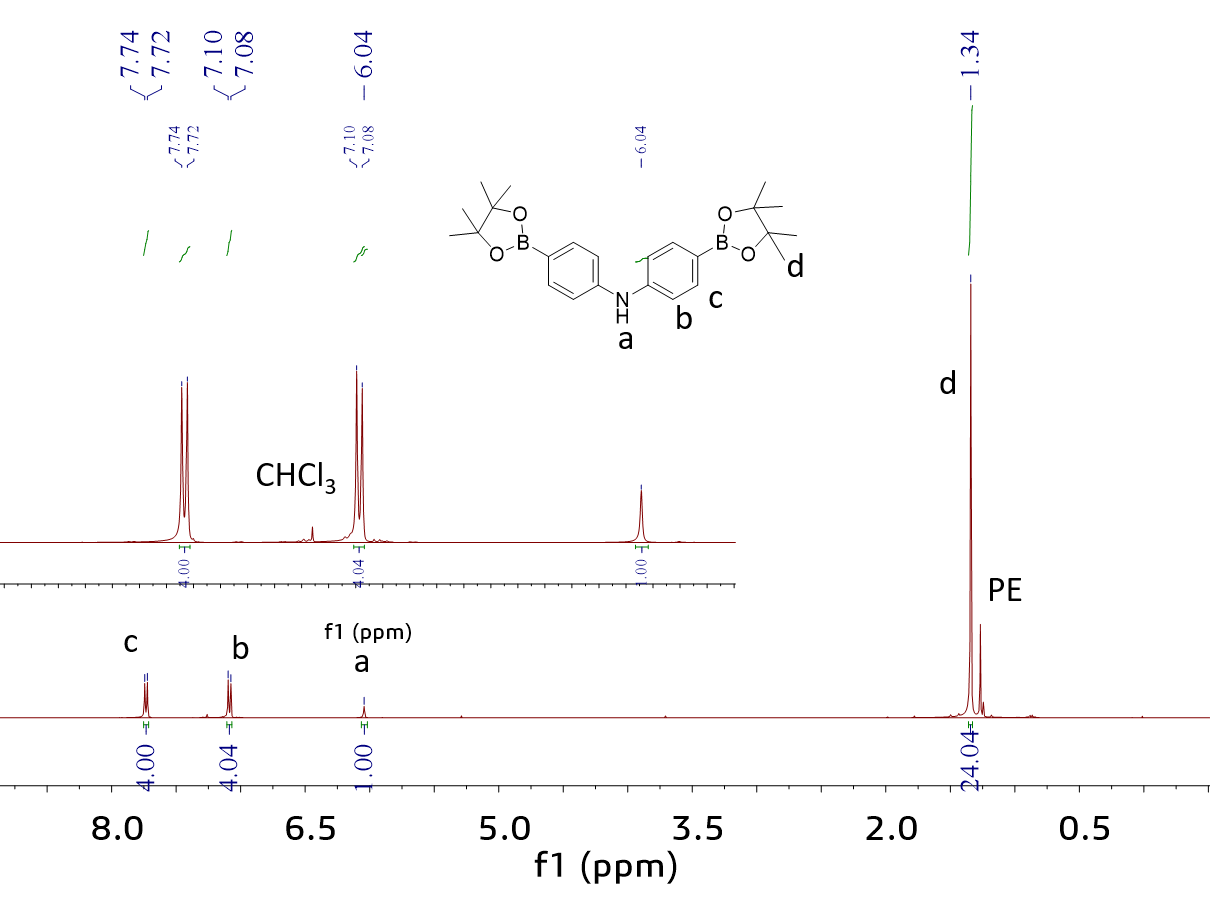


**Supplementary Fig. 15.** **The ^1^H-NMR spectrum of 2Ph-2Bpin-NH.**


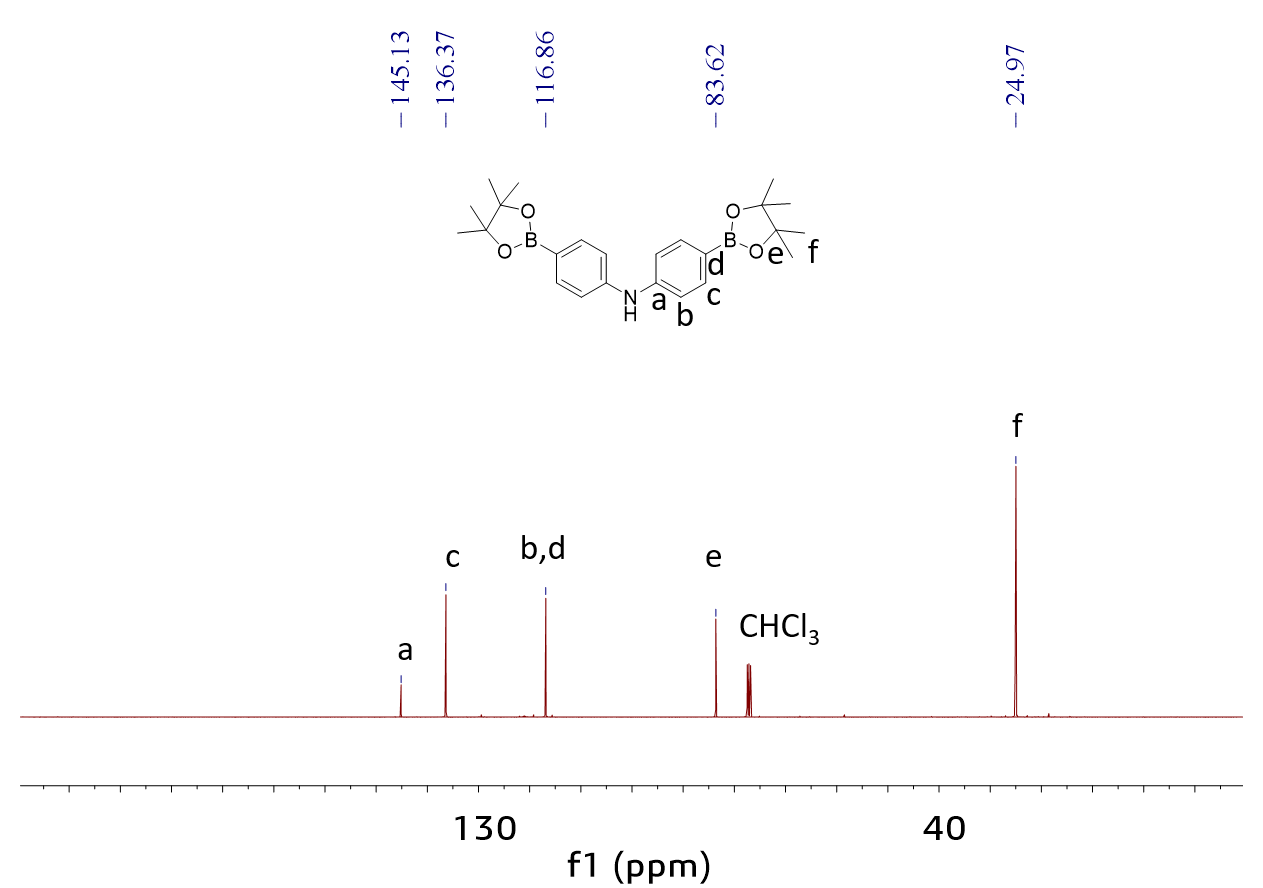


**Supplementary Fig. 16.** **The ^13^C-NMR spectrum of 2Ph-2Bpin-NH.**


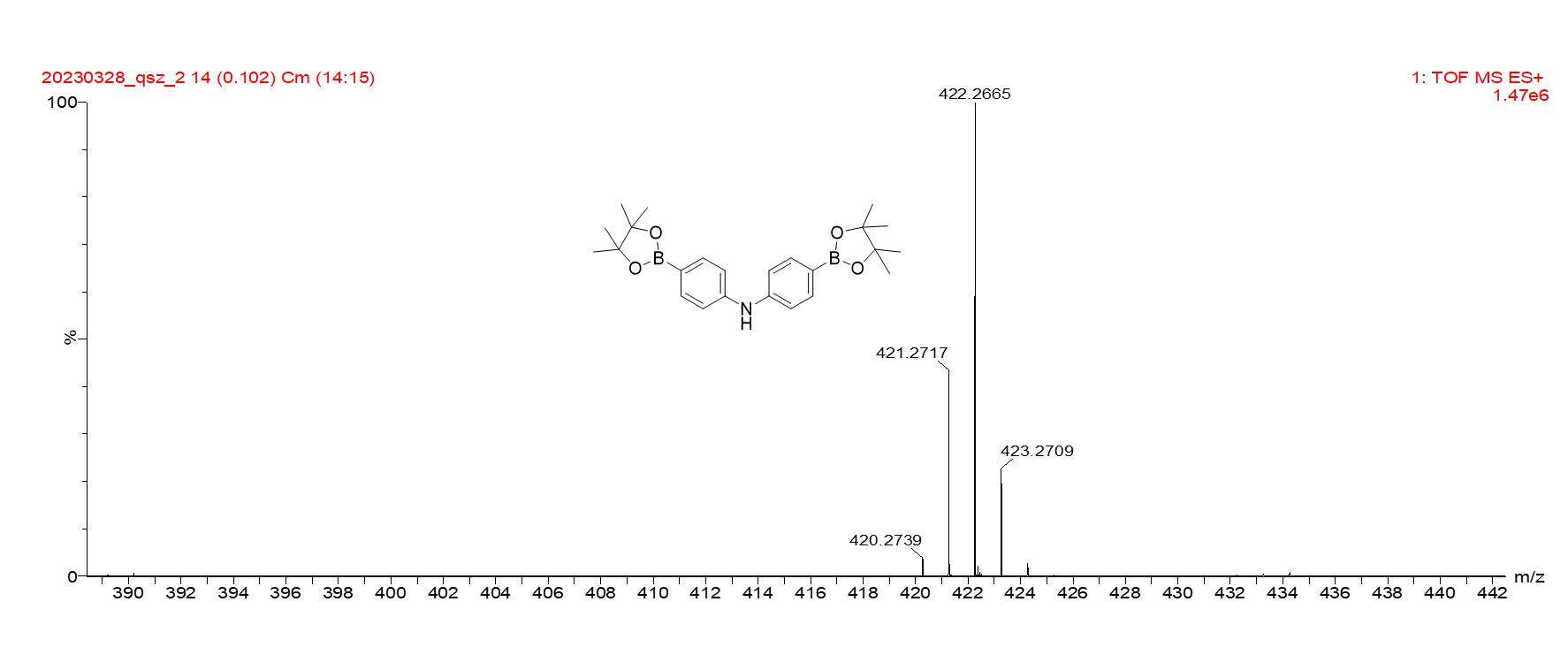


**Supplementary Fig. 17.** **The HRMS spectrum of 2Ph-2Bpin-NH.**

**2TPE-NH**

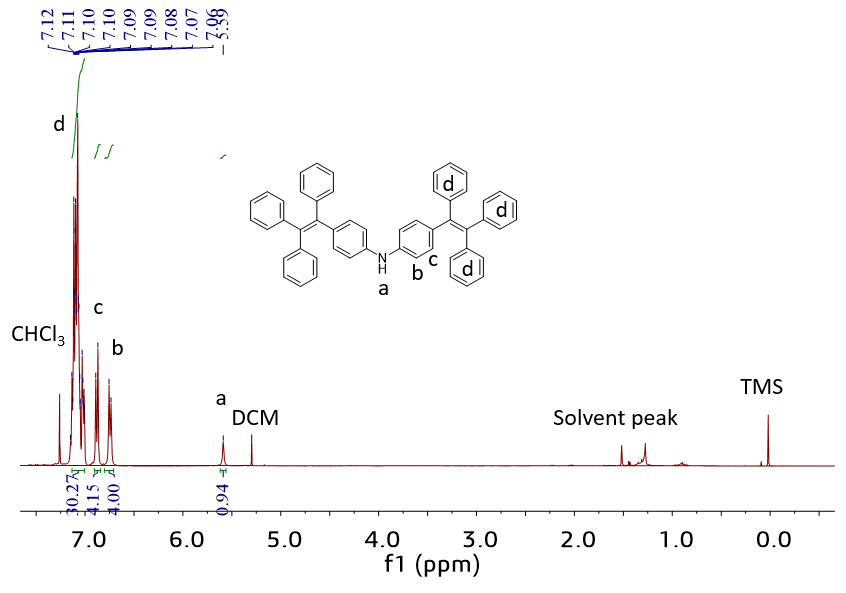


**Supplementary Fig. 18.** **The ^1^H-NMR spectrum of 2TPE-NH.**


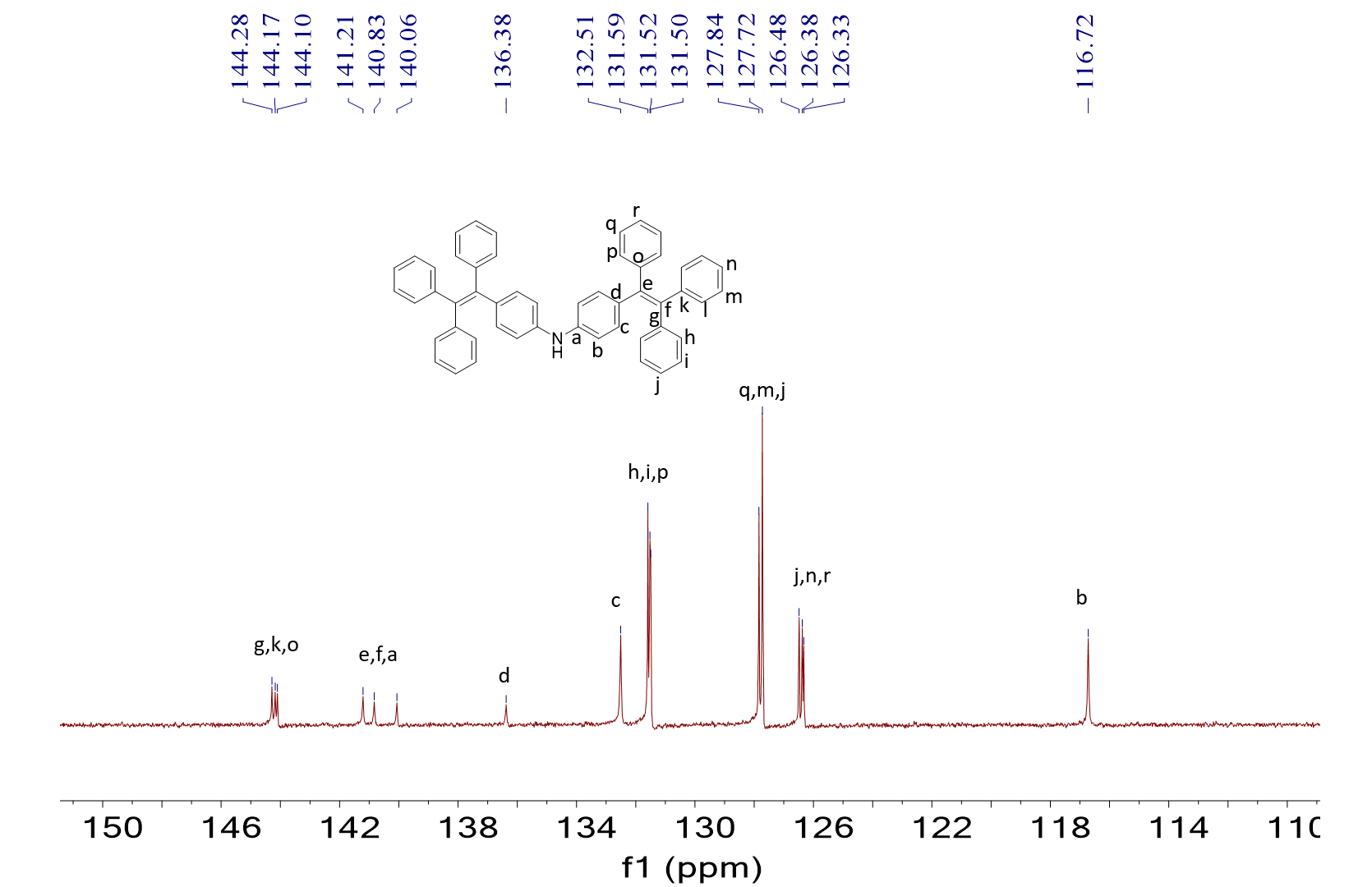


**Supplementary Fig. 19.** **The ^13^C-NMR spectrum of 2TPE-NH.**


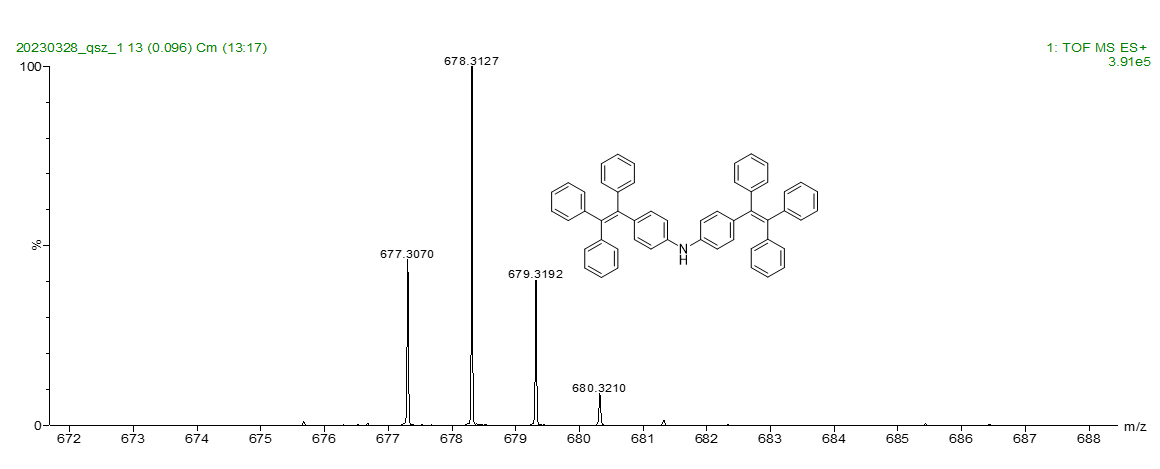


**Supplementary Fig. 20. The HRMS spectrum of 2TPE-NH.**

**2TPE-C_10_-STr**

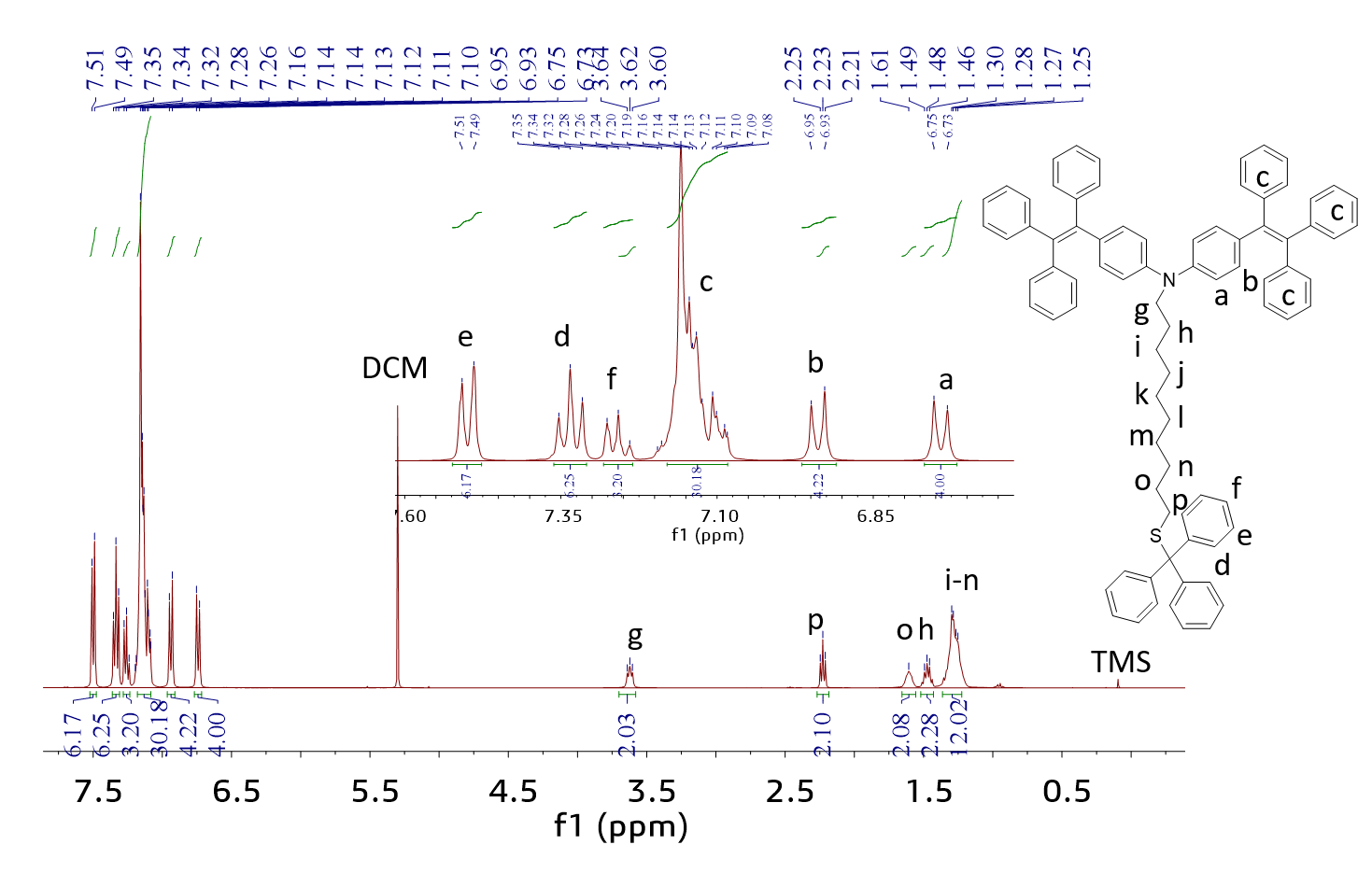


**Supplementary Fig. 21. The ^1^H-NMR spectrum of 2TPE-C_10_-STr.**


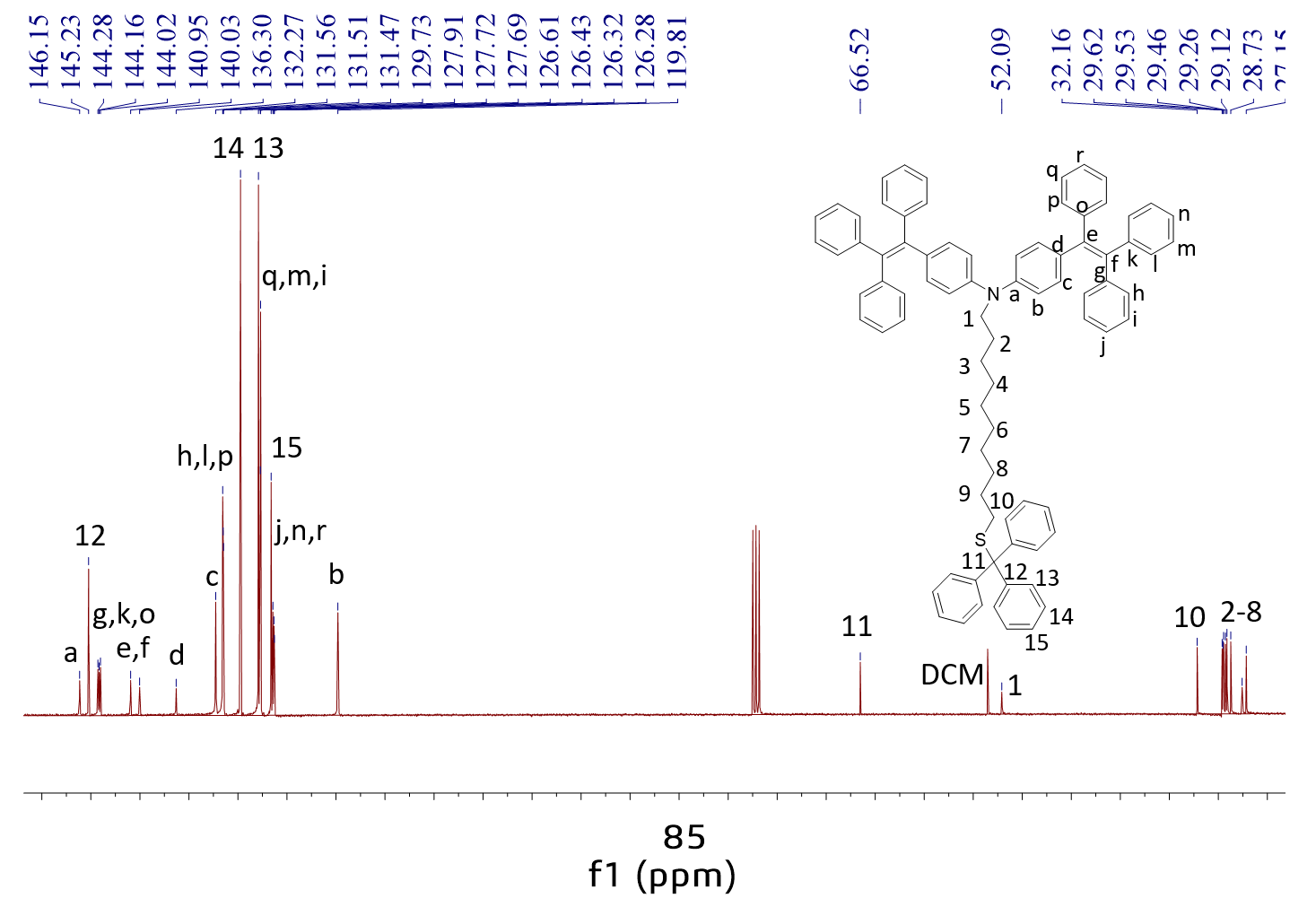


**Supplementary Fig. 22.** **The ^13^C-NMR spectrum of 2TPE-C_10_-STr.**


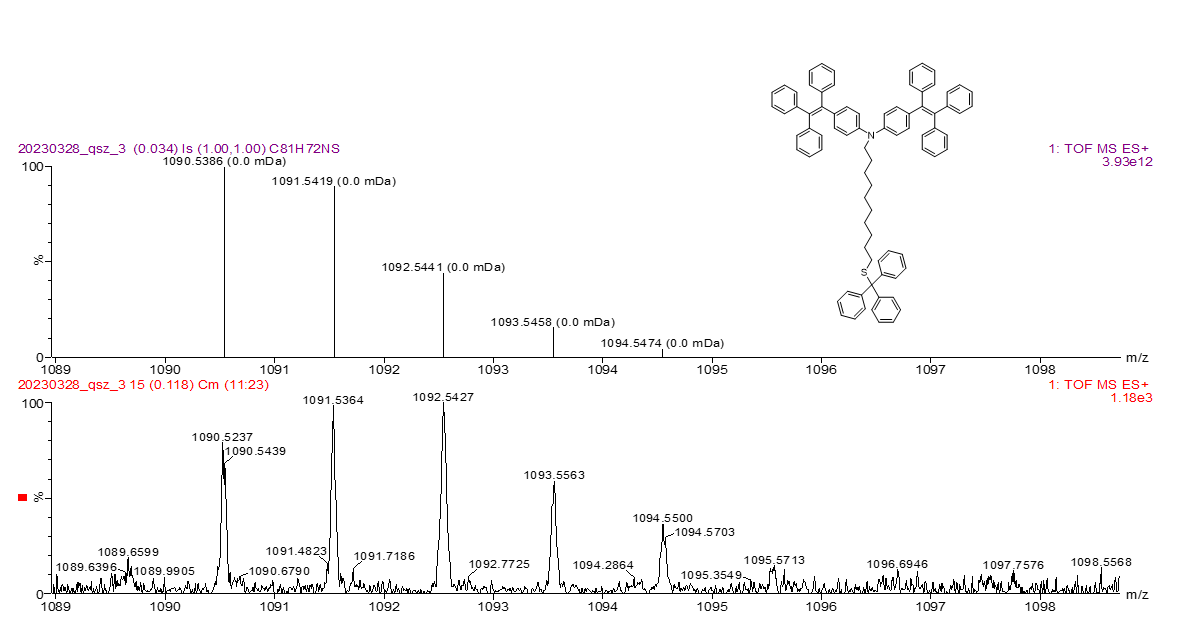


**Supplementary Fig. 23.** **The HRMS spectrum of 2TPE-C_10_-STr.**

**di-TPE**

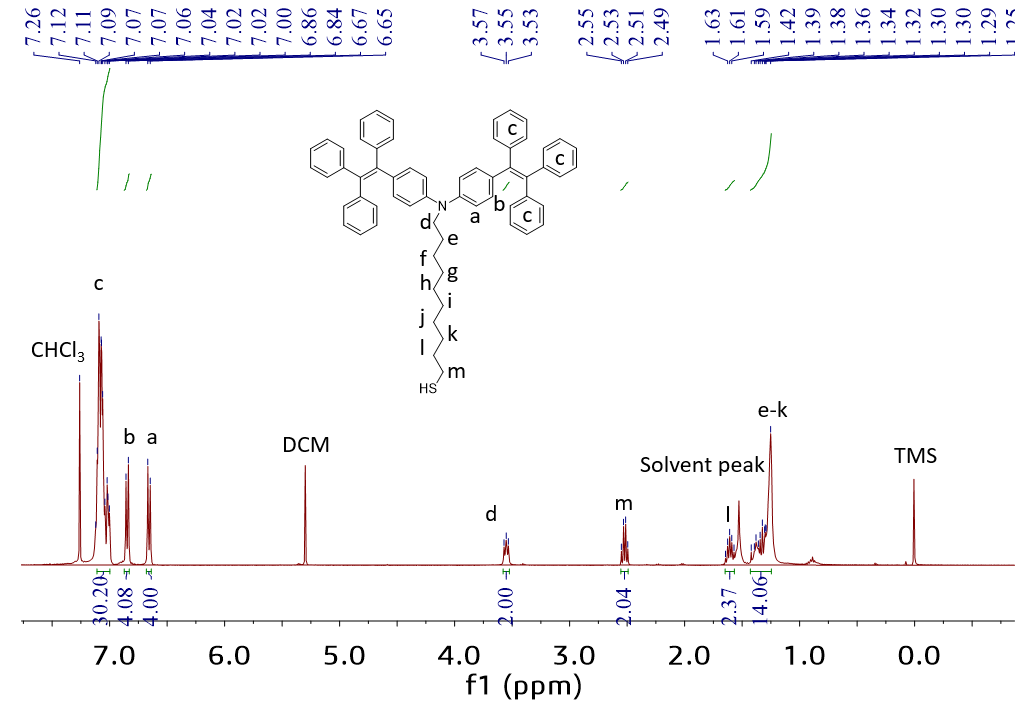


**Supplementary Fig. 24.** **The ^1^H-NMR spectrum of di-TPE.**


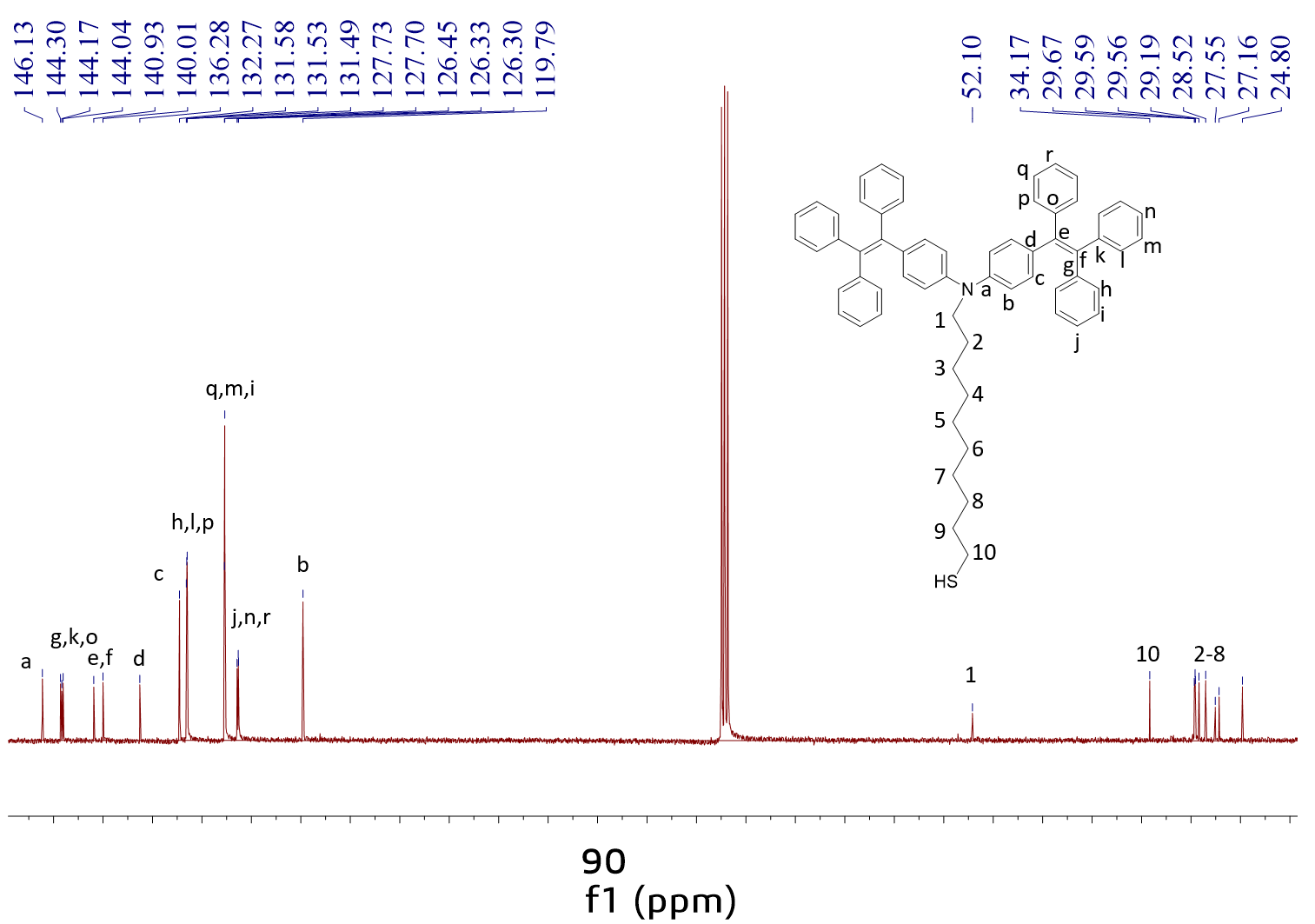


**Supplementary Fig. 25.** **The ^13^C-NMR spectrum of di-TPE.**


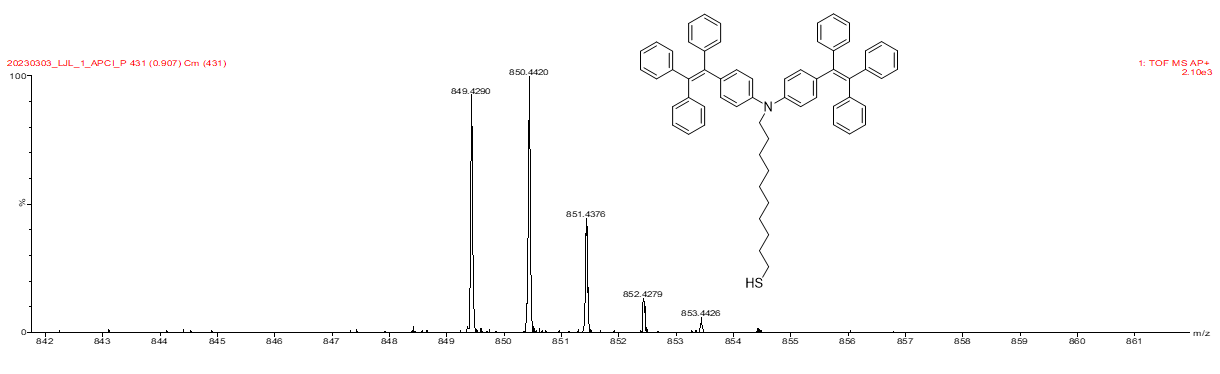


**Supplementary Fig. 26.** **The HRMS spectrum of di-TPE.**

**tri-TPE**

**Supplementary Fig. 27.The synthetic route of tri-TPE.**

**2Br-TPE-OMe**

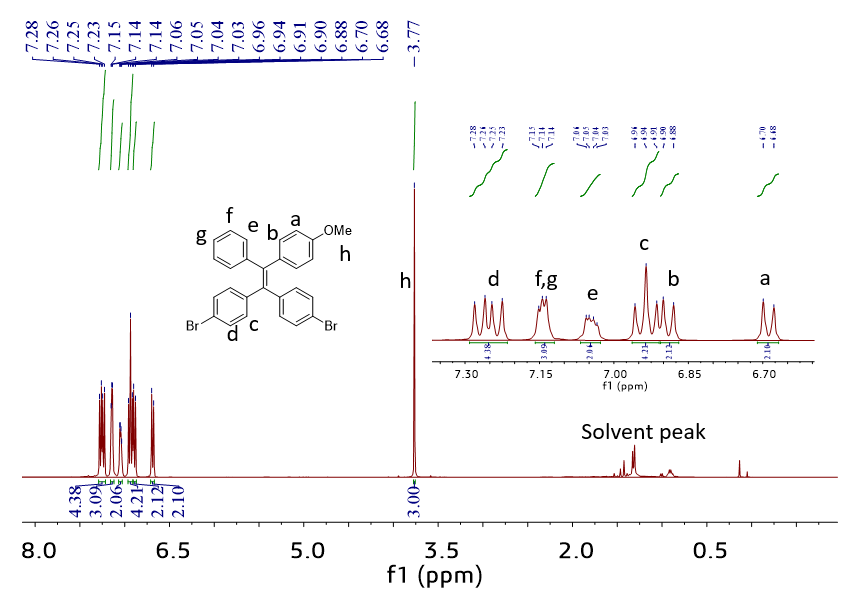


**Supplementary Fig. 28.** **The ^1^H-NMR spectrum of 2Br-TPE-OMe.**


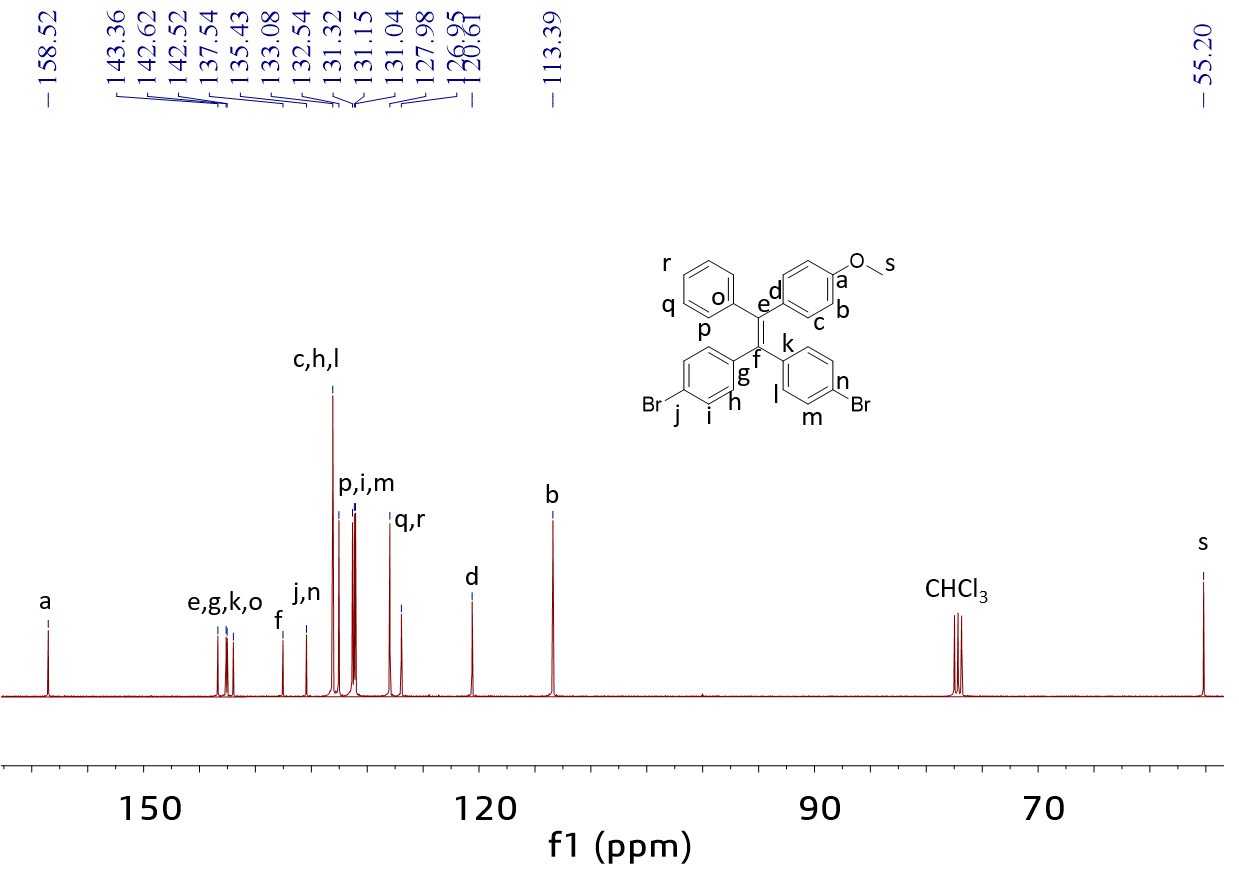


**Supplementary Fig. 29. The ^13^C-NMR spectrum of 2Br-TPE-OMe.**


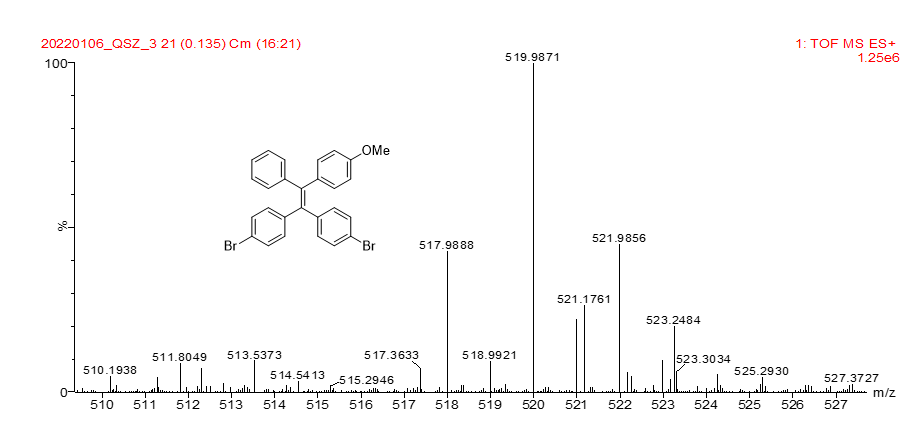
**Supplementary Fig. 30.** **The HRMS spectrum of 2Br-TPE-OMe.**

**2Br-TPE-OH**

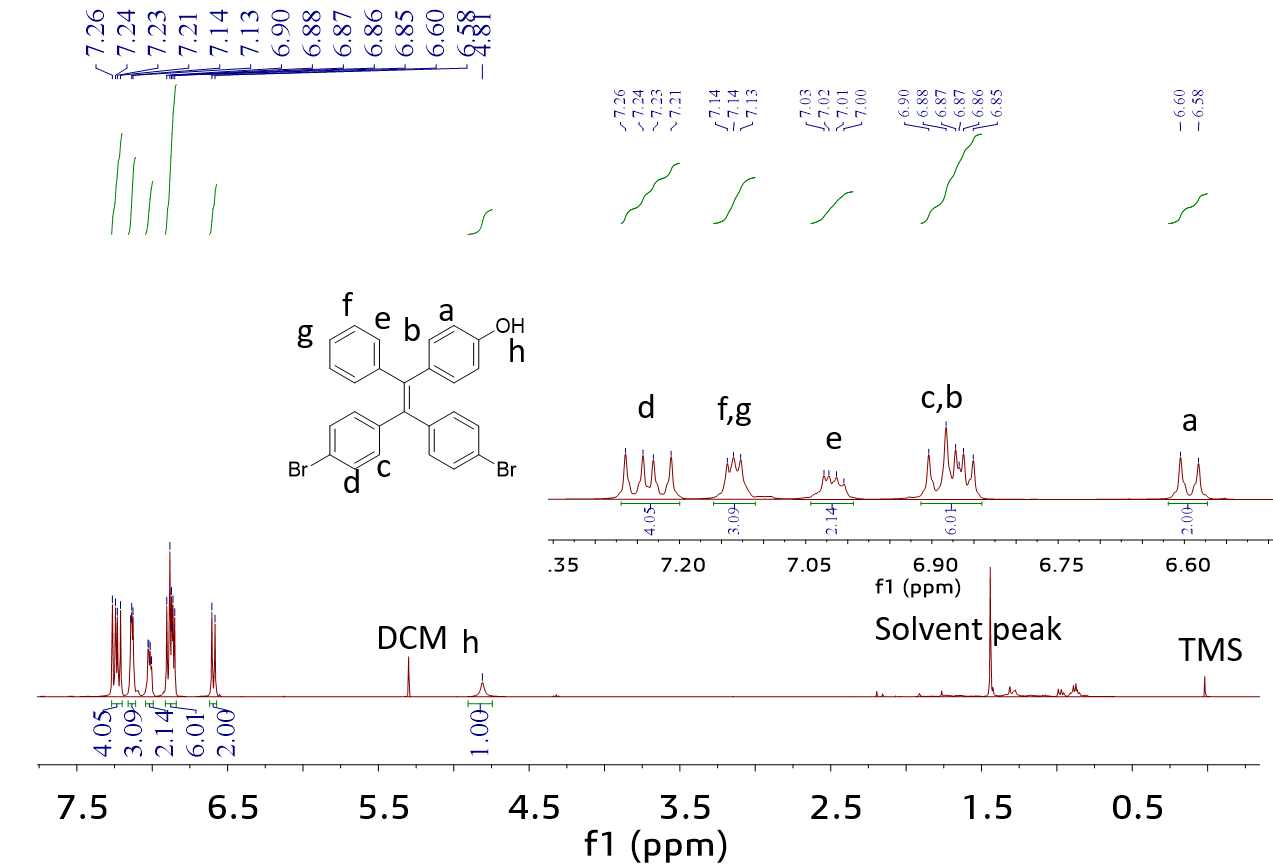


**Supplementary Fig. 31.** **The ^1^H-NMR spectrum of 2Br-TPE-OH.**


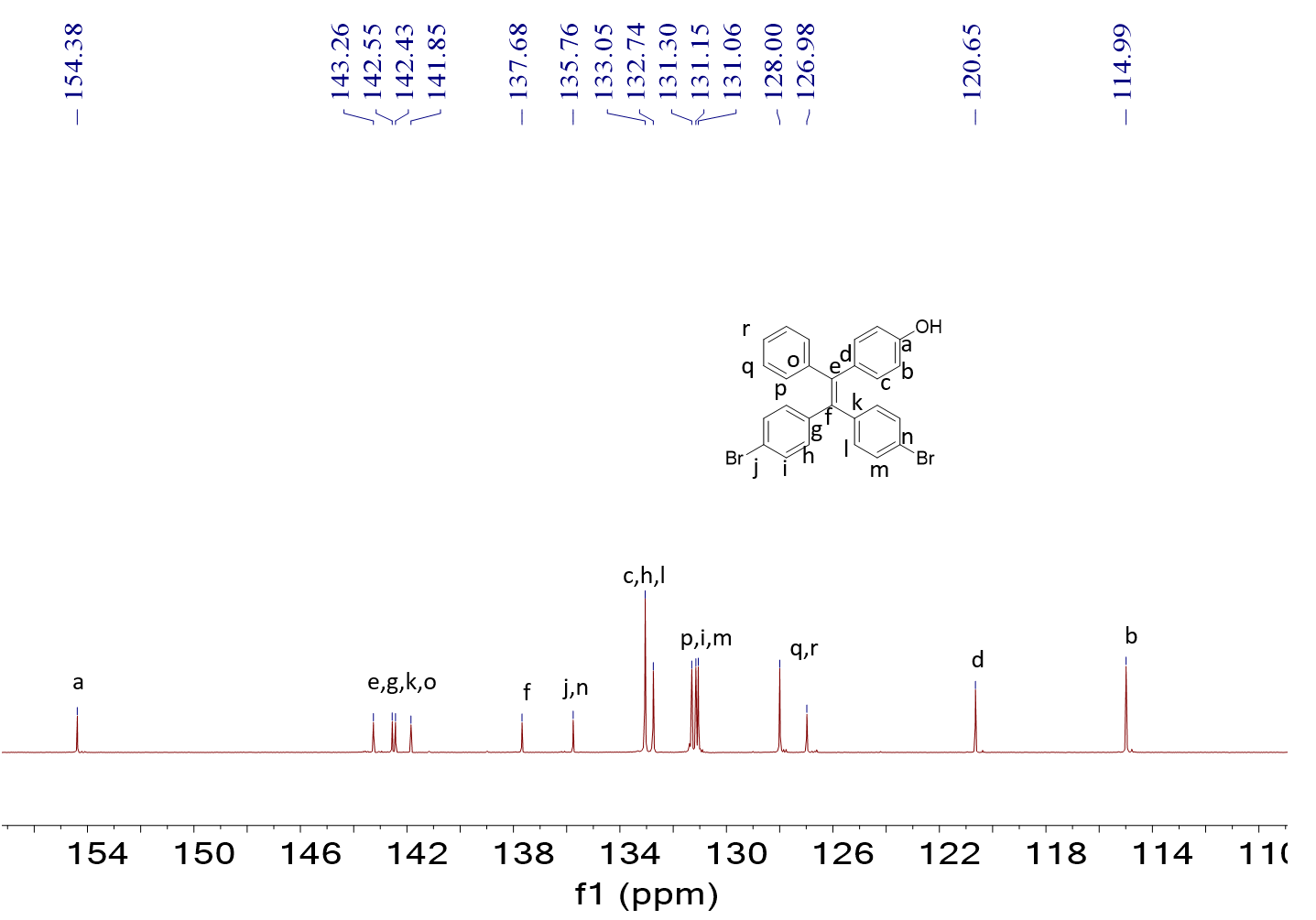


**Supplementary Fig. 32.** **The ^13^C-NMR spectrum of 2Br-TPE-OH.**


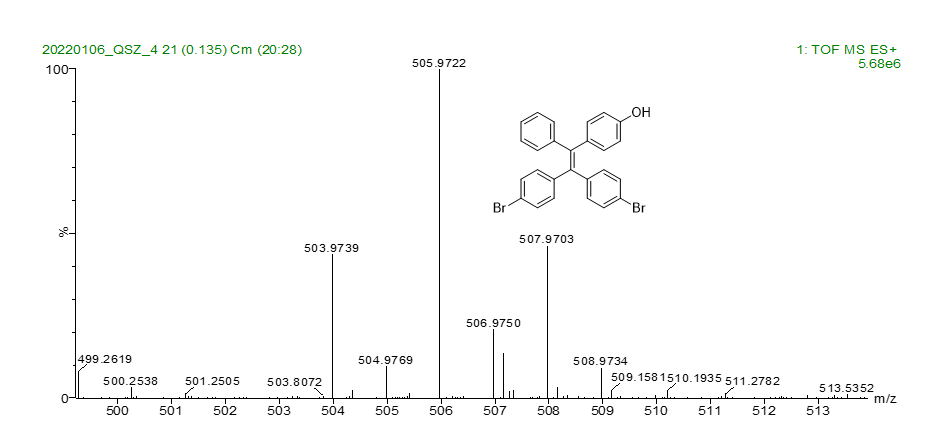
**Supplementary Fig. 33.** **The HRMS spectrum of 2Br-TPE-OH.**

**2Br-TPE-C_10_-STr**

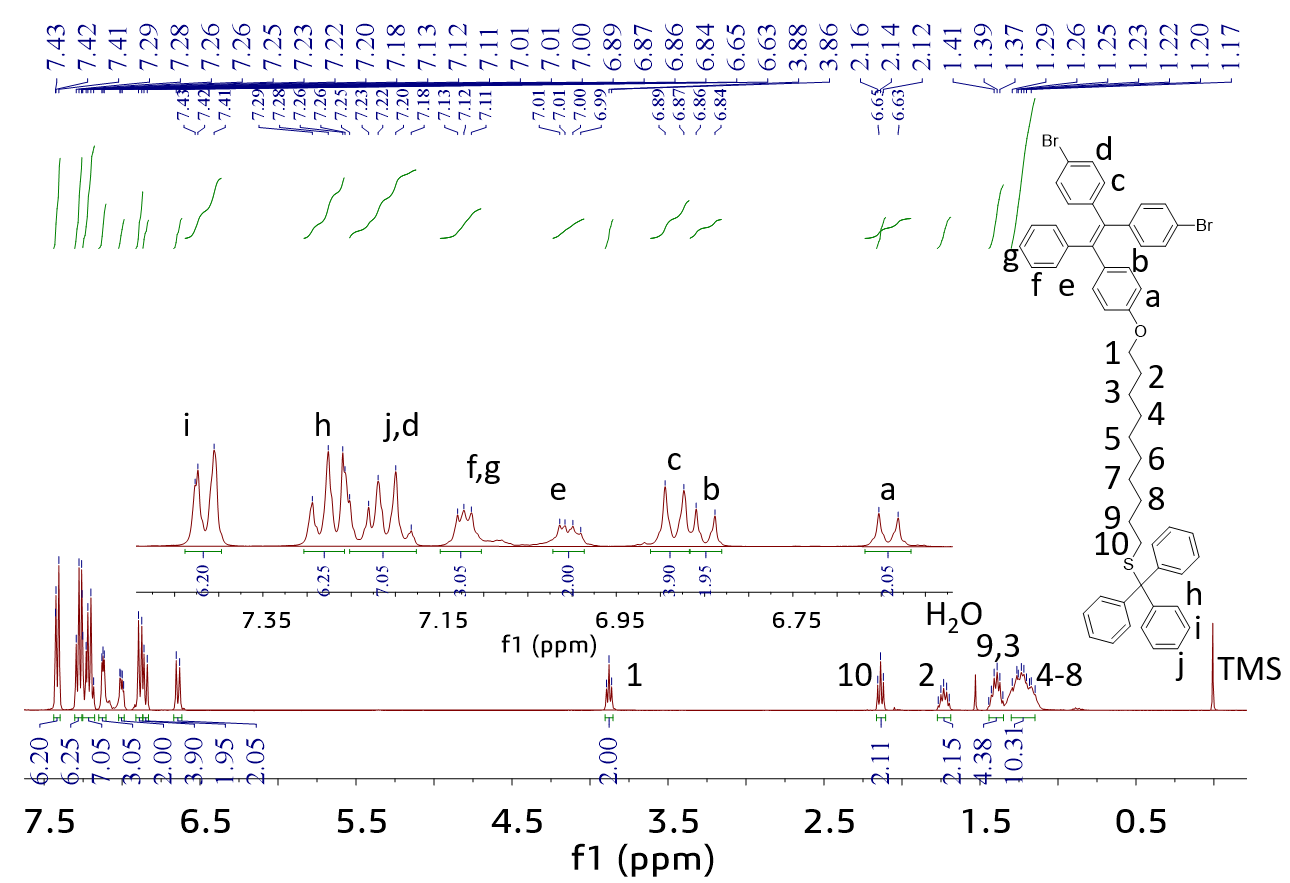


**Supplementary Fig. 34.** **The ^1^H-NMR spectrum of 2Br-TPE-C_10_-STr.**


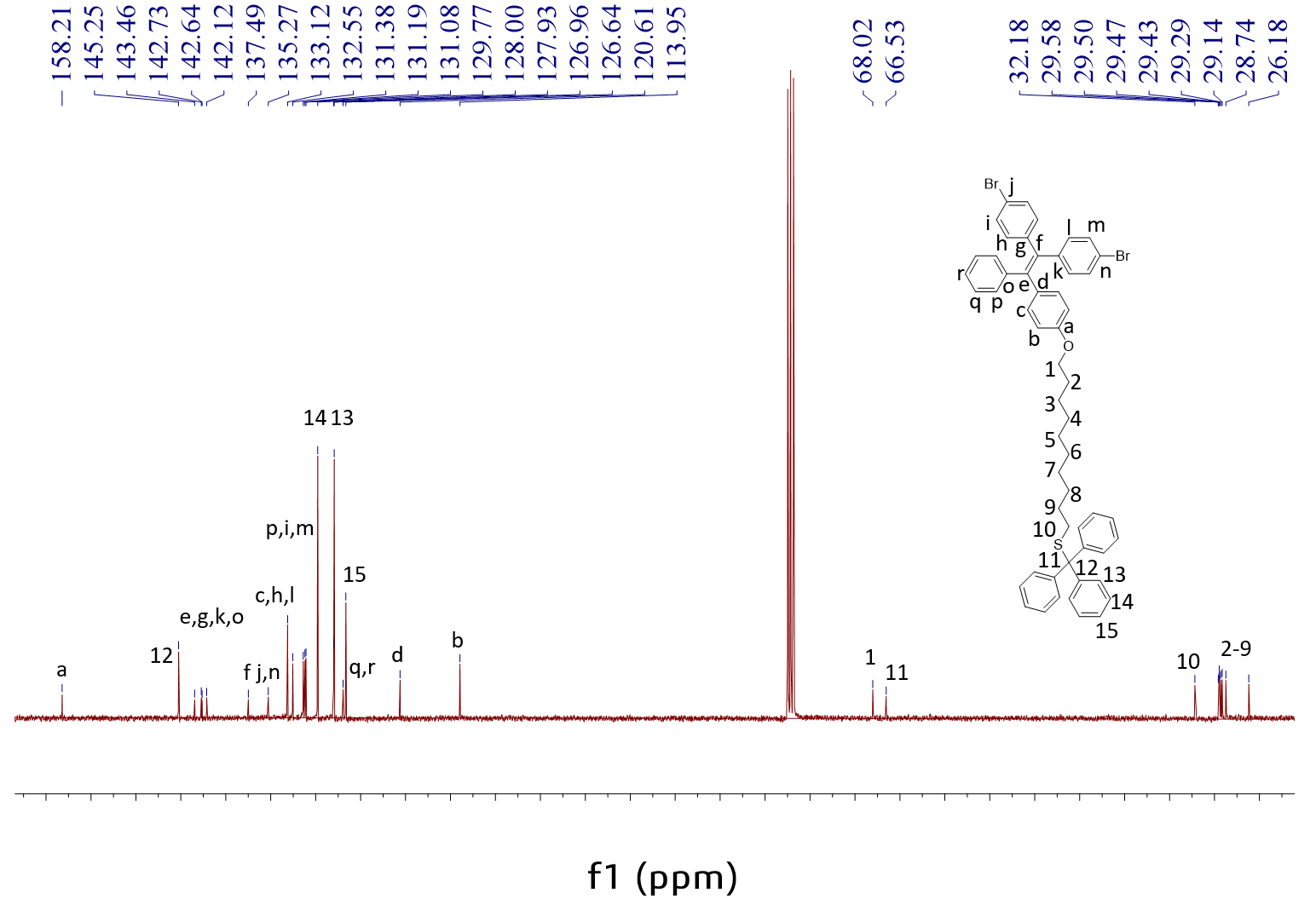


**Supplementary Fig. 35.** **The ^13^C-NMR spectrum of 2Br-TPE-C_10_-STr.**


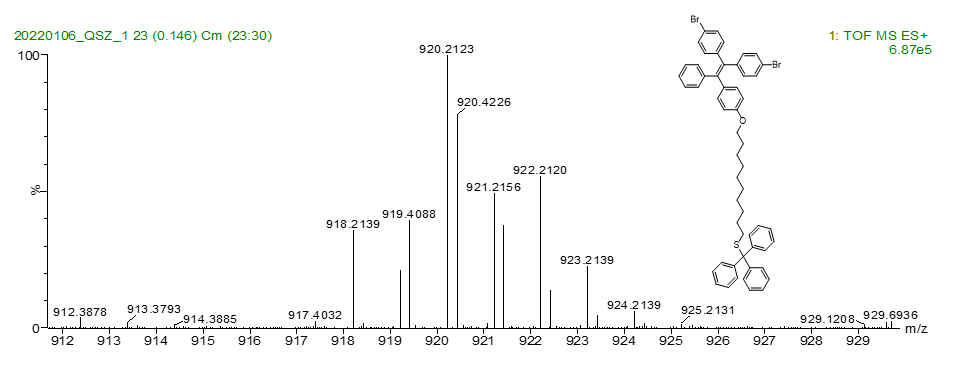


**Supplementary Fig. 36.** **The HRMS spectrum of 2Br-TPE-C_10_-STr.**

**3TPE-C10-STr**

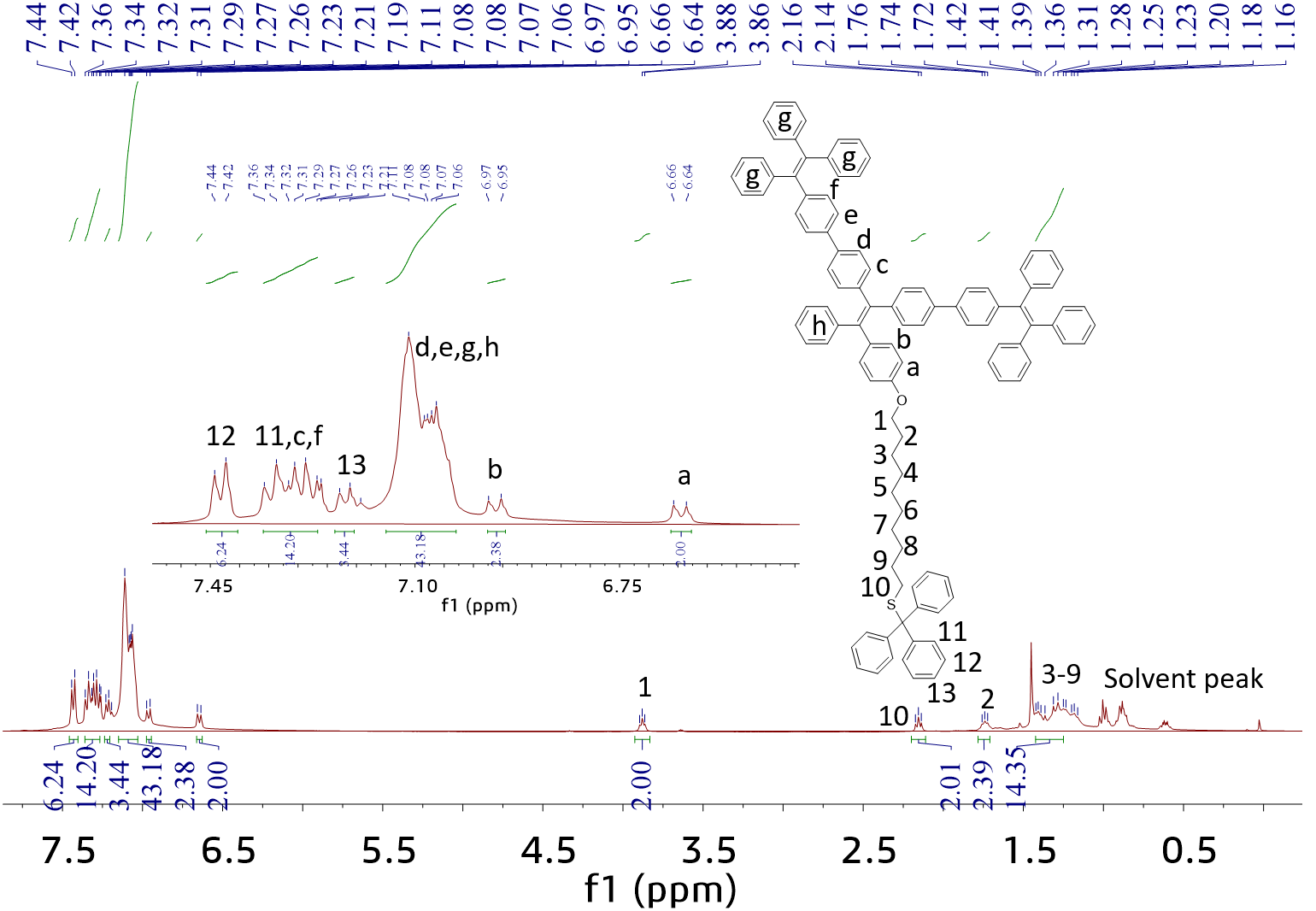


**Supplementary Fig. 37.** **The ^1^H-NMR spectrum of 3TPE-C_10_-STr.**


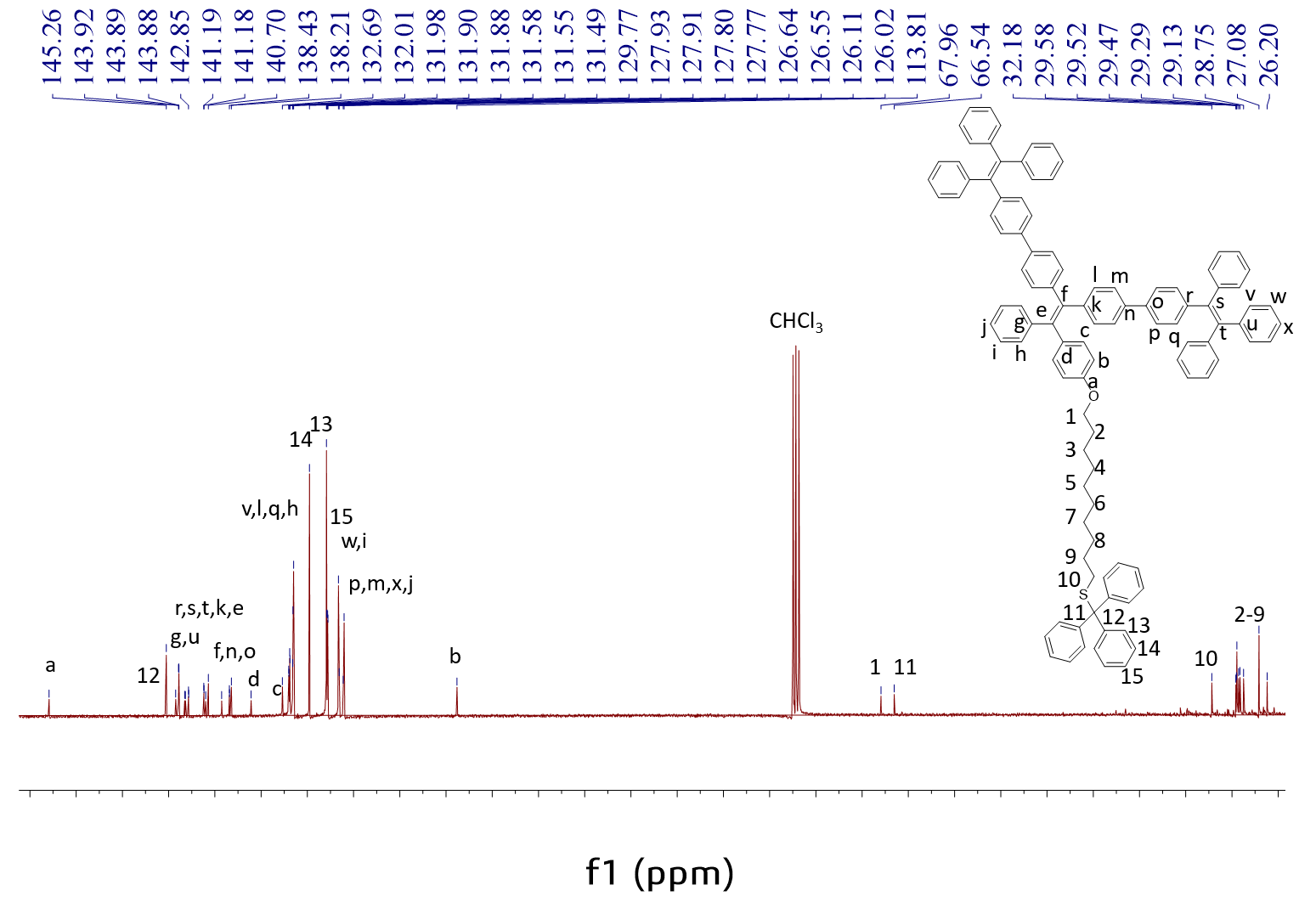


**Supplementary Fig. 38.** **The ^13^C-NMR spectrum of 3TPE-C_10_-STr.**


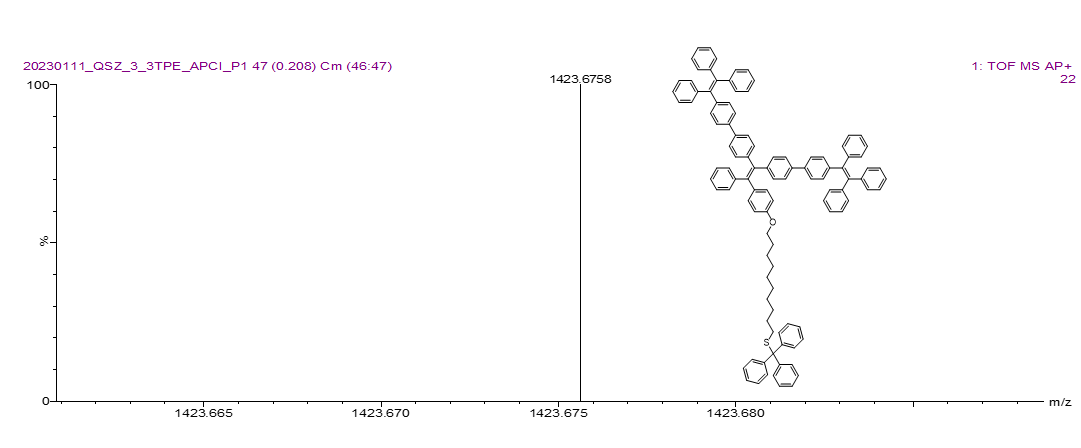


**Supplementary Fig. 39. The HRMS spectrum of 3TPE-C_10_-STr.**

**tri-TPE**

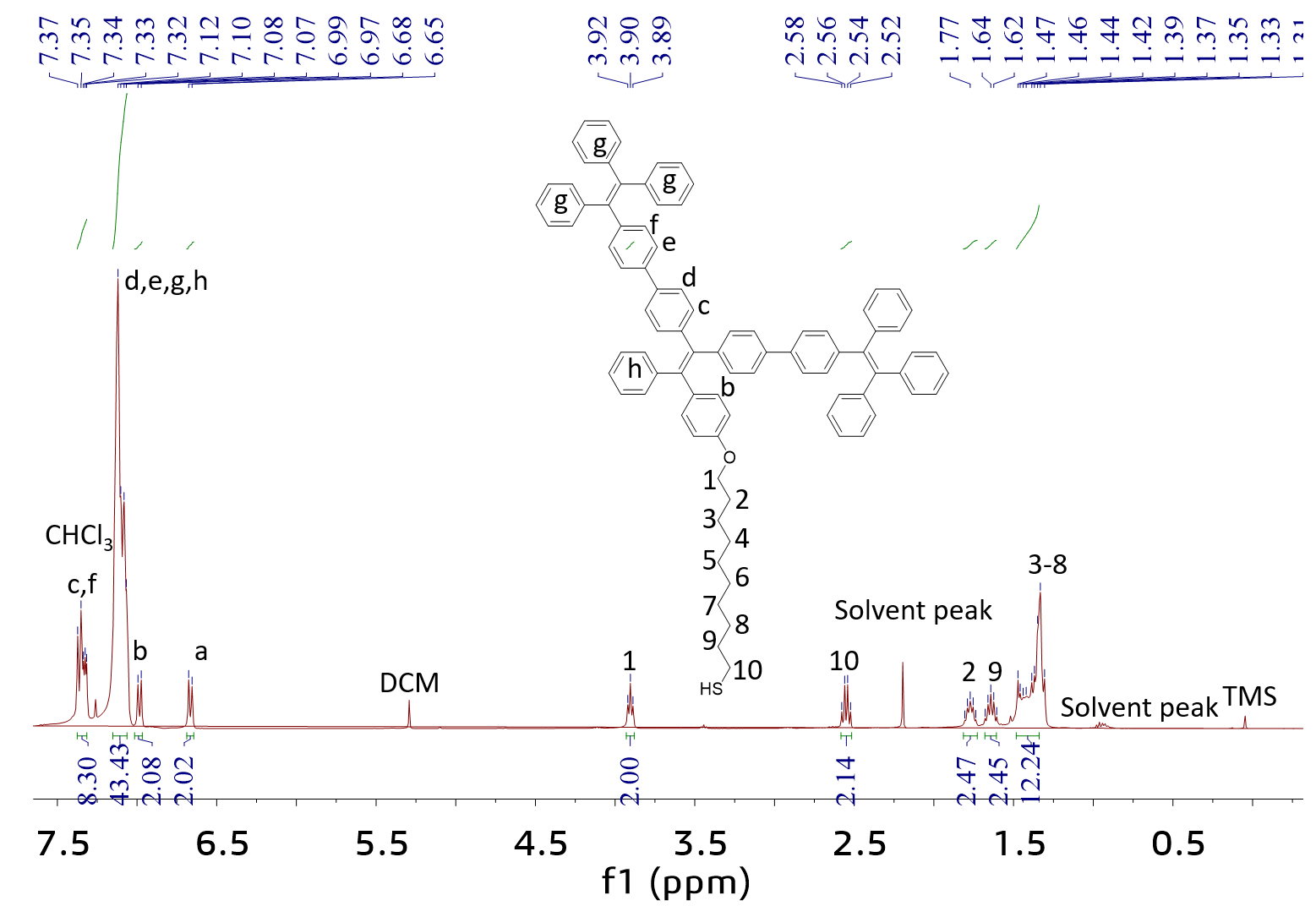


**Supplementary Fig. 40.** **The ^1^H-NMR spectrum of tri-TPE.**


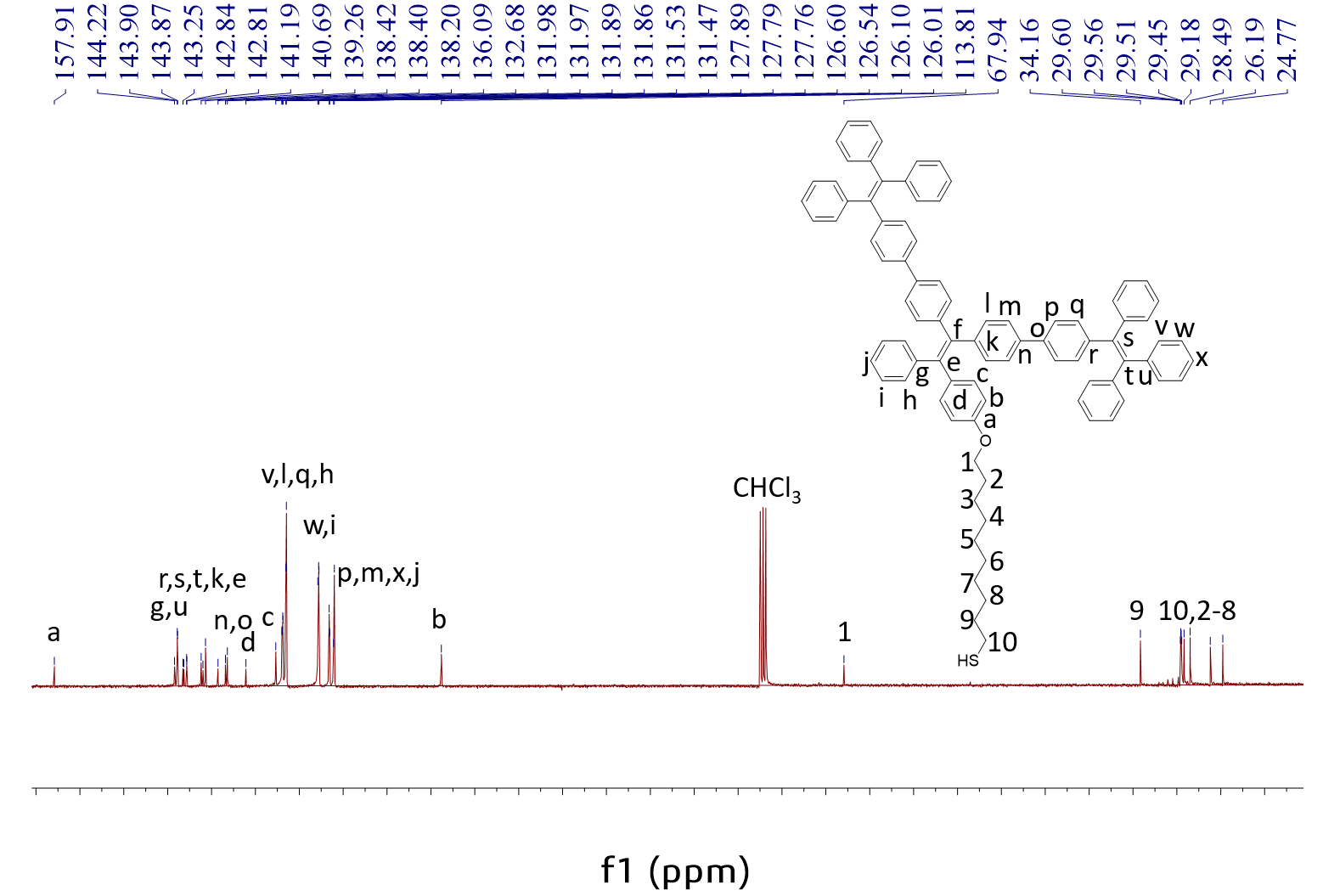


**Supplementary Fig. 41.** **The ^13^C-NMR spectrum of tri-TPE.**


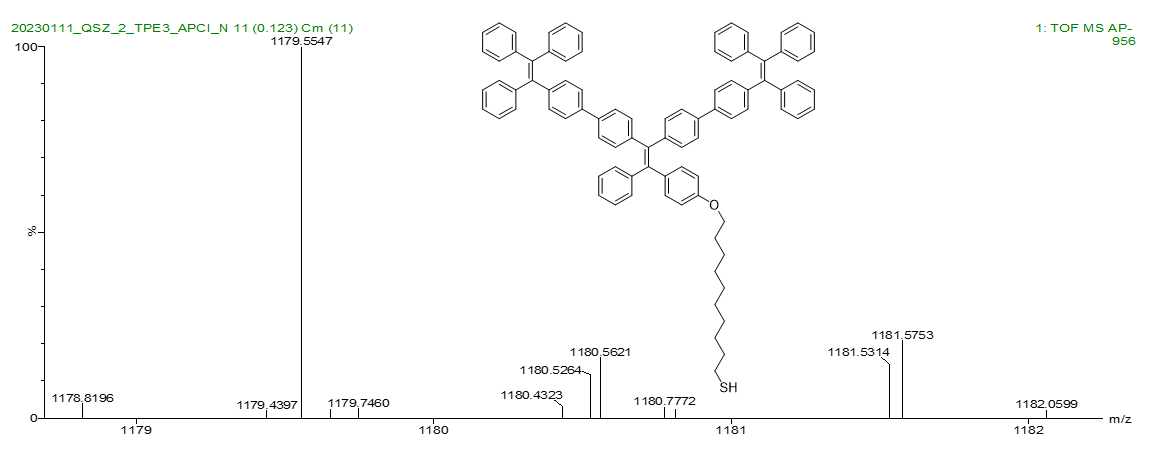


**Supplementary Fig. 42. The HRMS spectrum of tri-TPE.**

**tetra-TPE**

**Supplementary Fig. 43.The synthetic route of tetra-TPE.**

**3Br-TPE-OMe**

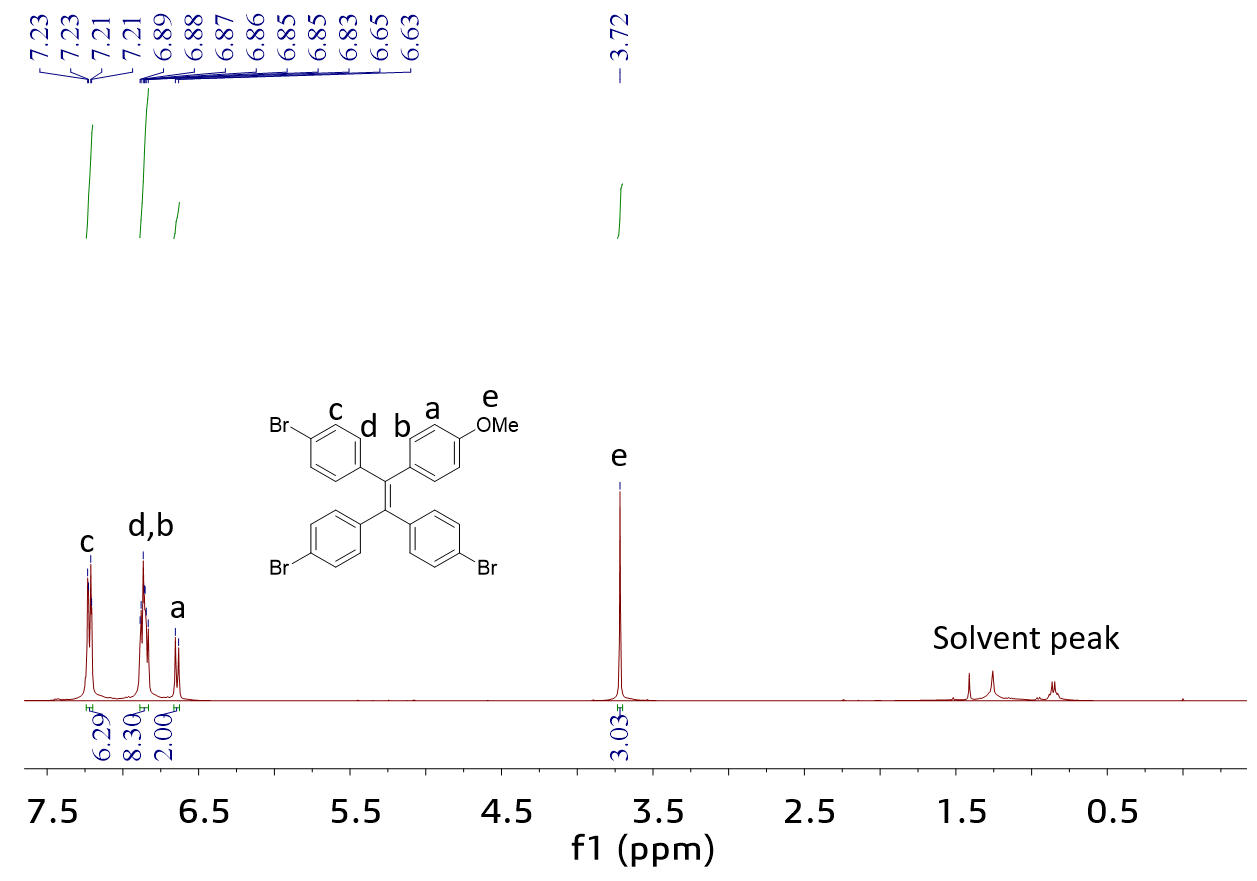


**Supplementary Fig. 44.** **The ^1^H-NMR spectrum of 3Br-TPE-OMe.**


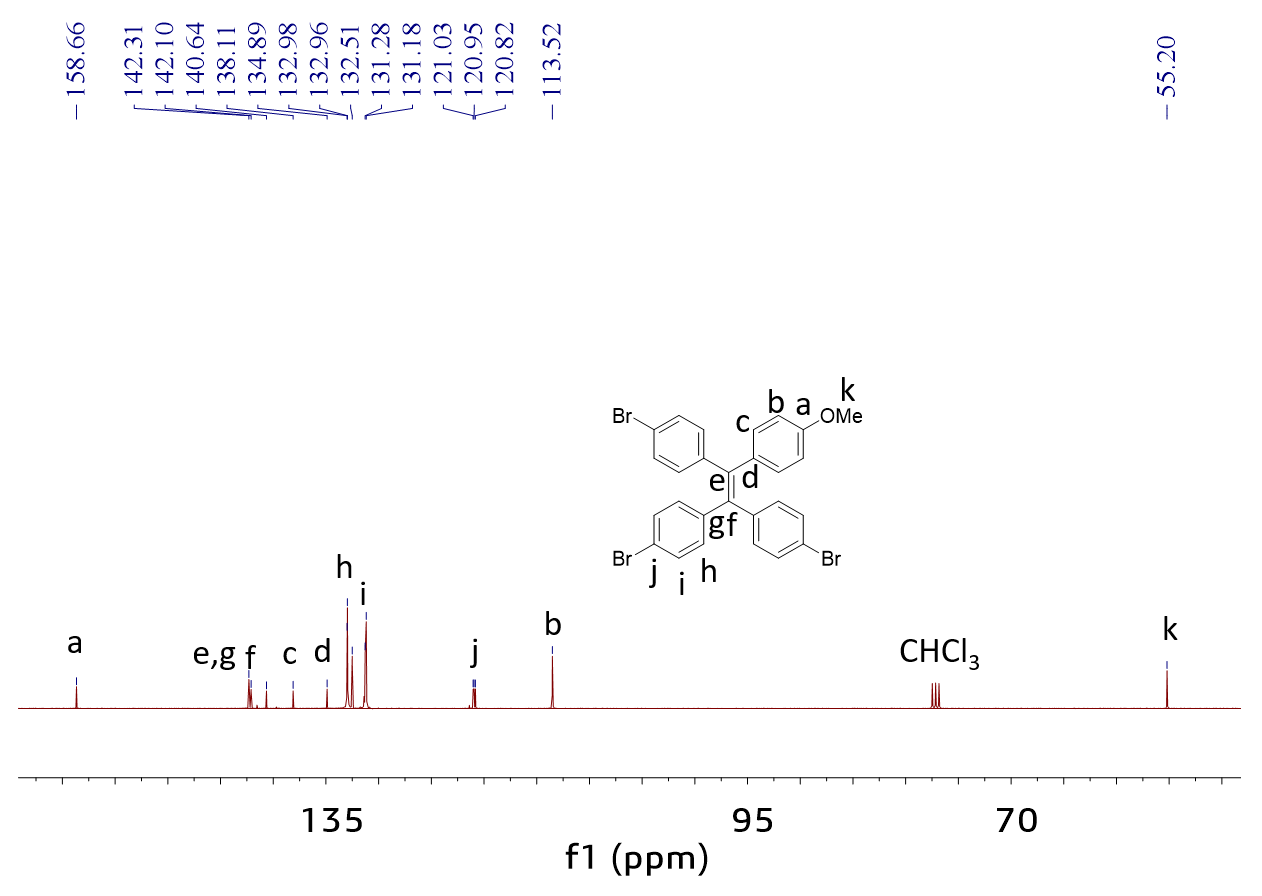


**Supplementary Fig. 45. The ^13^C-NMR spectrum of 3Br-TPE-OMe.**


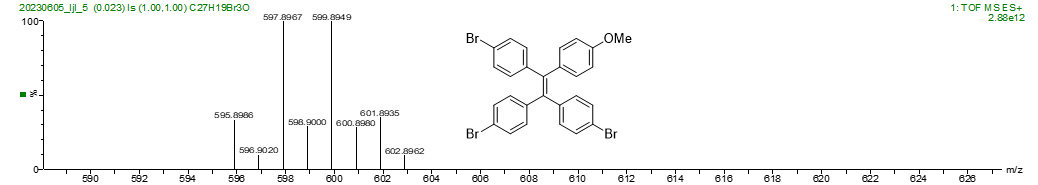


**Supplementary Fig. 46.** **The HRMS spectrum of 3Br-TPE-OMe.**

**4TPE-OMe**

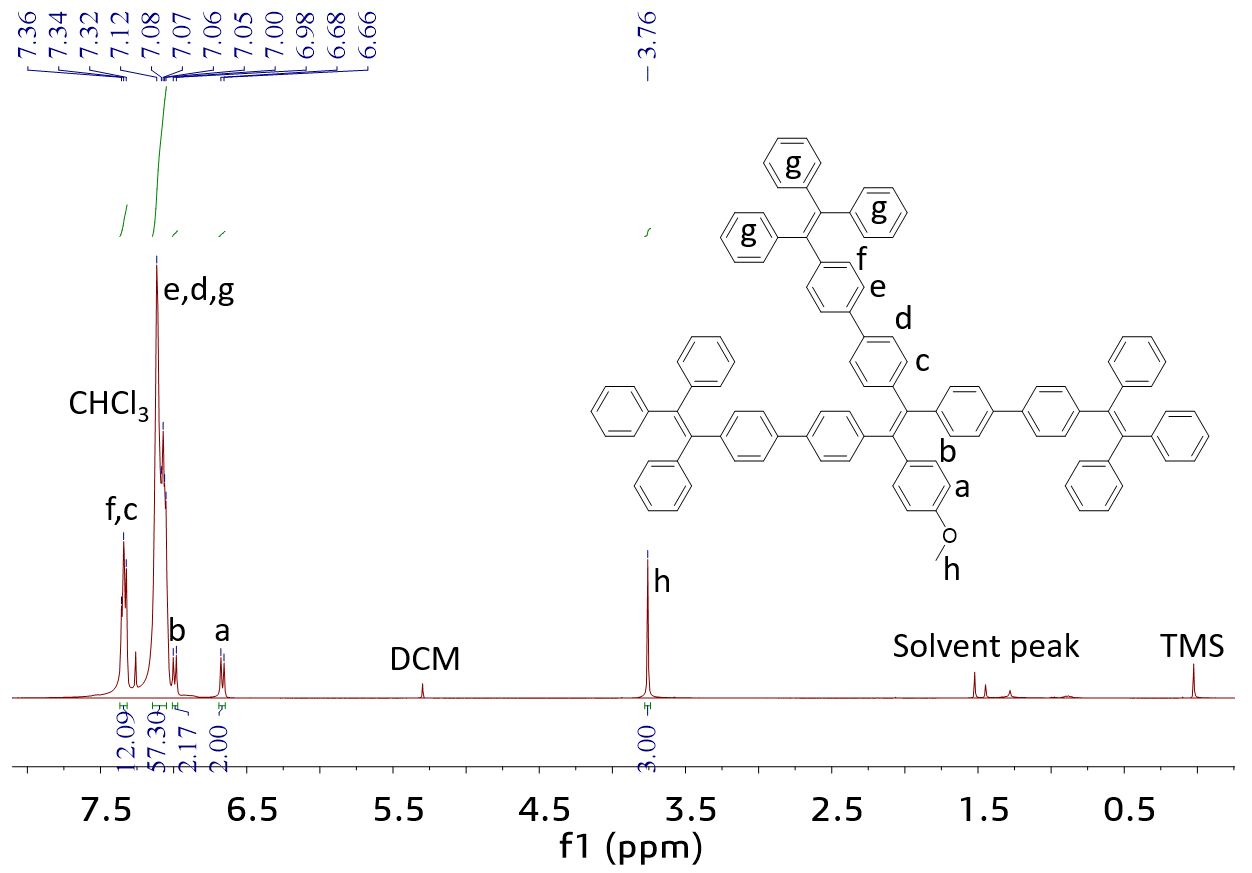


**Supplementary Fig. 47.** **The ^1^H-NMR spectrum of 4TPE-OMe.**


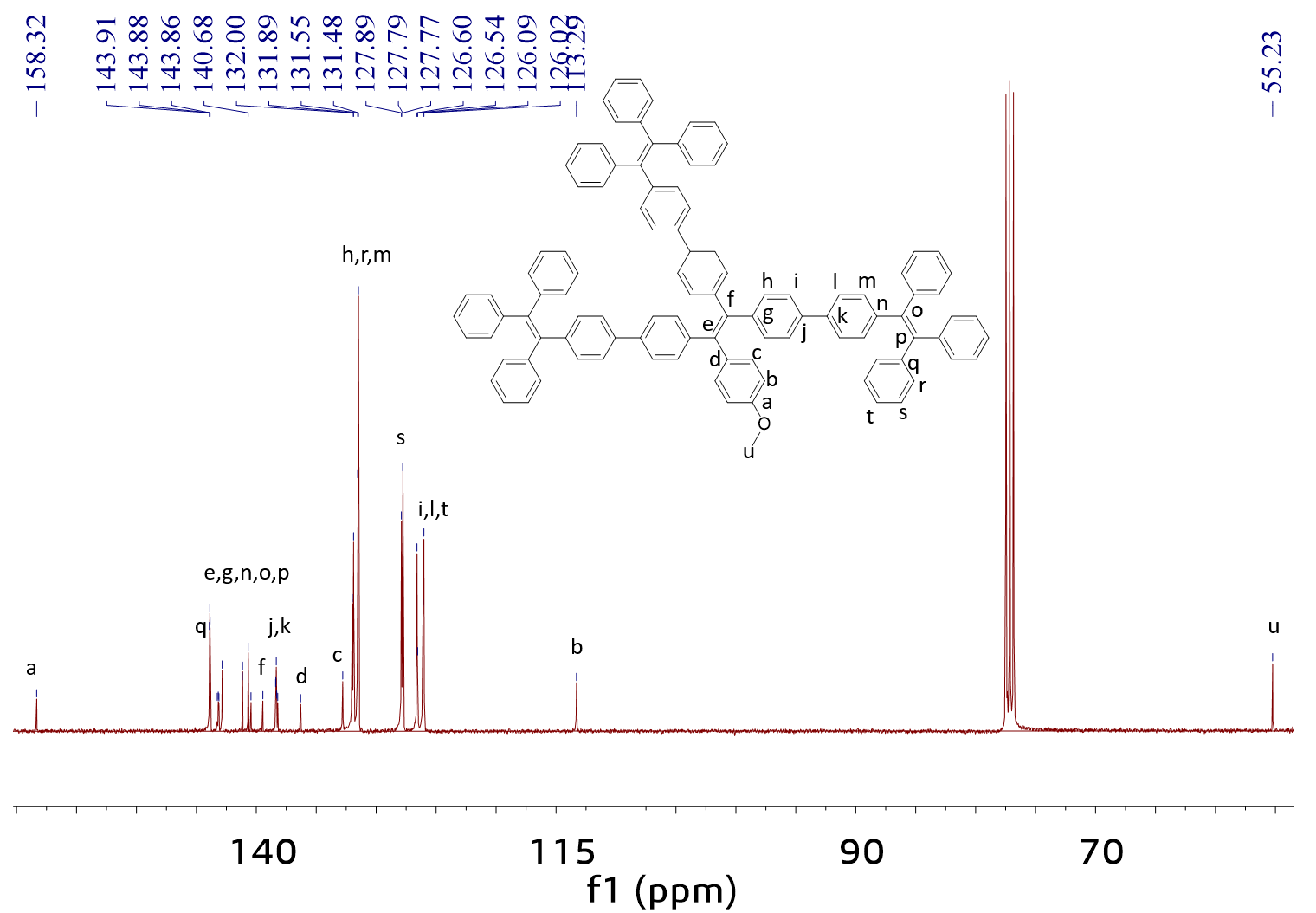


**Supplementary Fig. 48. The ^13^C-NMR spectrum of 4TPE-OMe.**


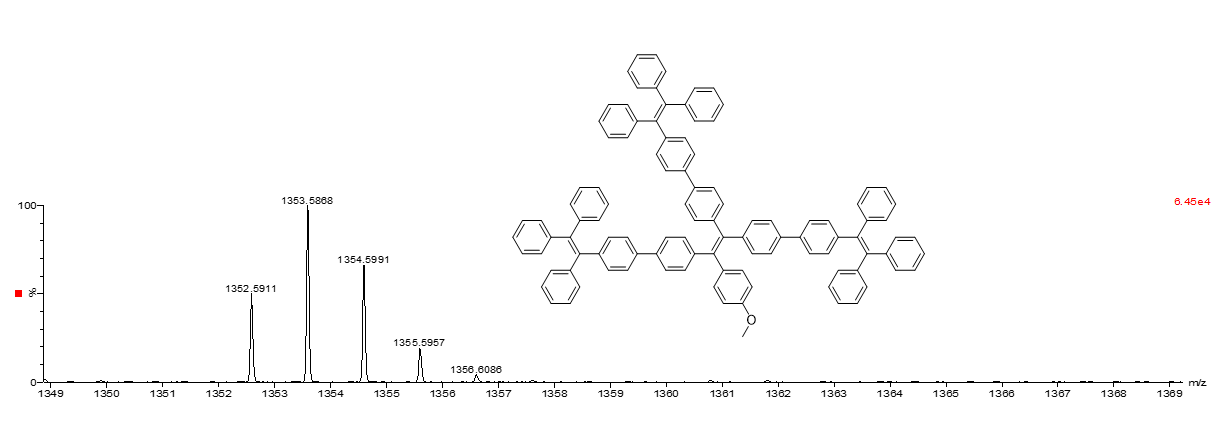


**Supplementary Fig. 49.** **The HRMS spectrum of 4TPE-OMe.**

**4TPE-OH**

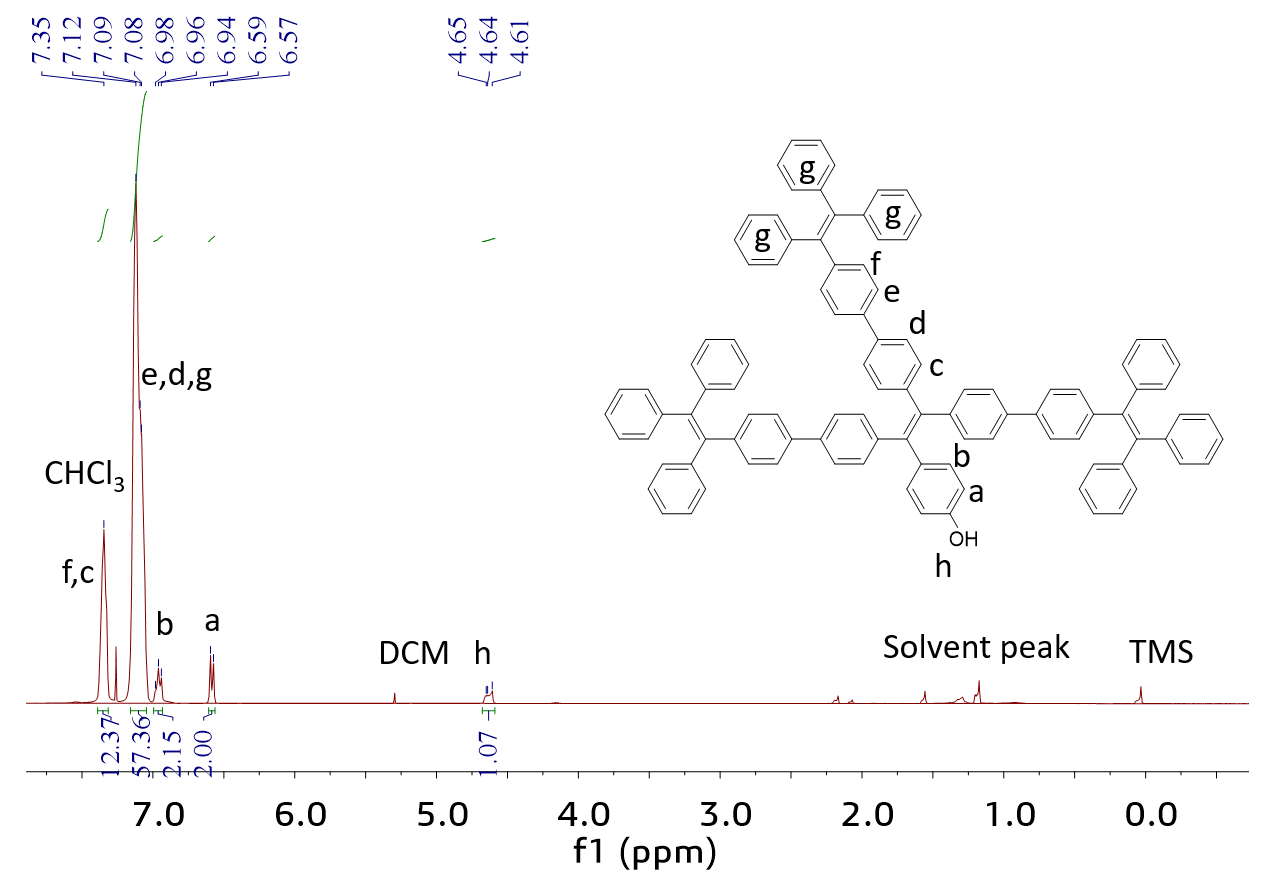


**Supplementary Fig. 50.** **The ^1^H-NMR spectrum of 4TPE-OH.**


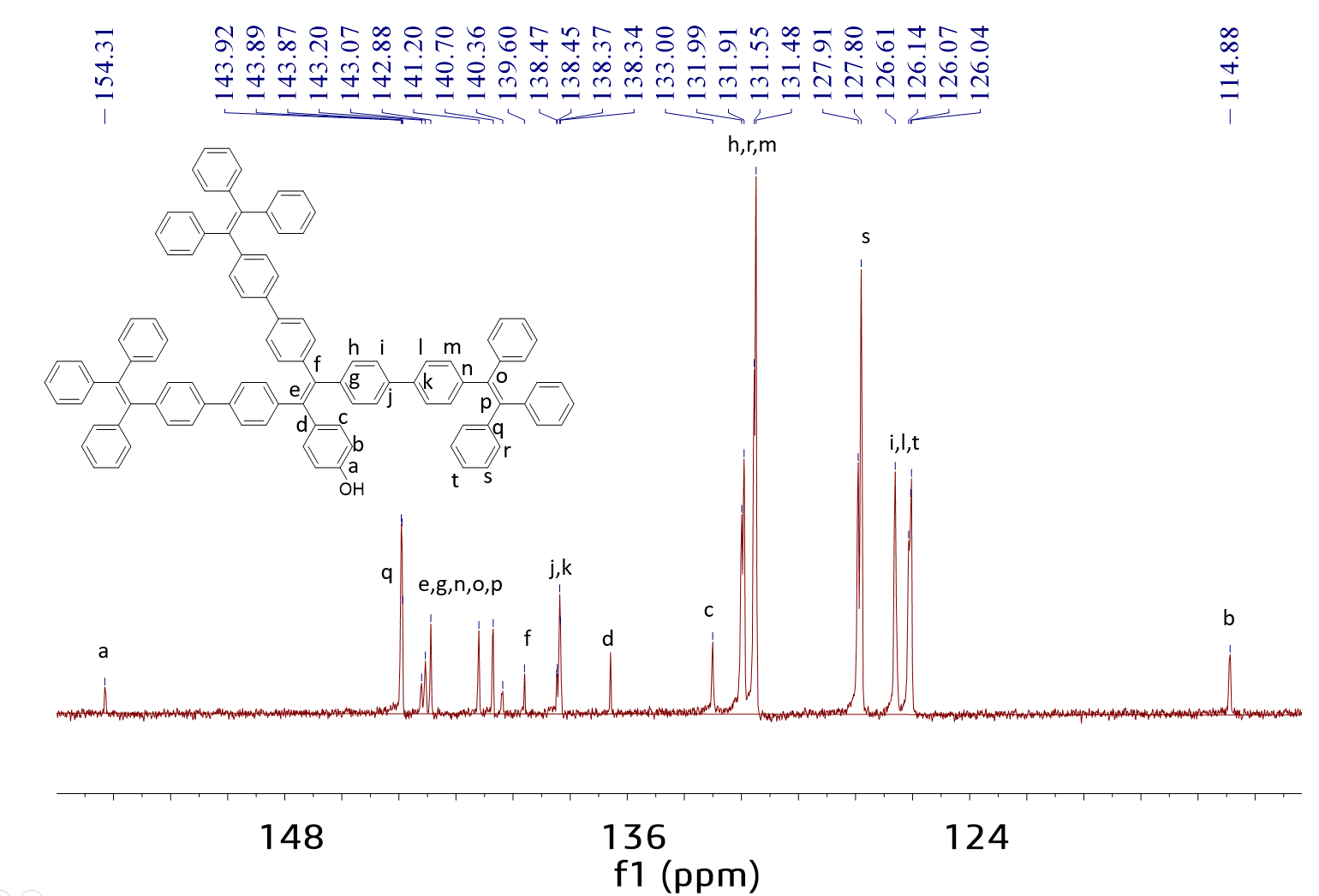


**Supplementary Fig. 51.** **The 13C-NMR spectrum of 4TPE-OH.**


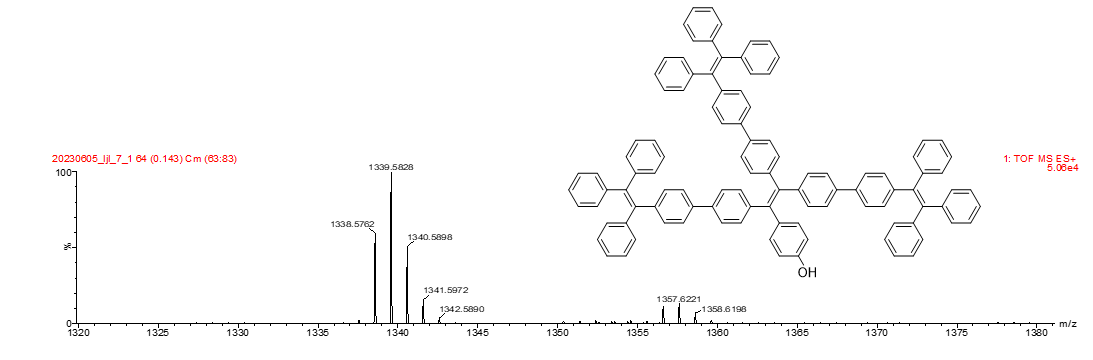


**Supplementary Fig. 52.** **The HRMS spectrum of 4TPE-OH.**

**4TPE-C_10_-STr**

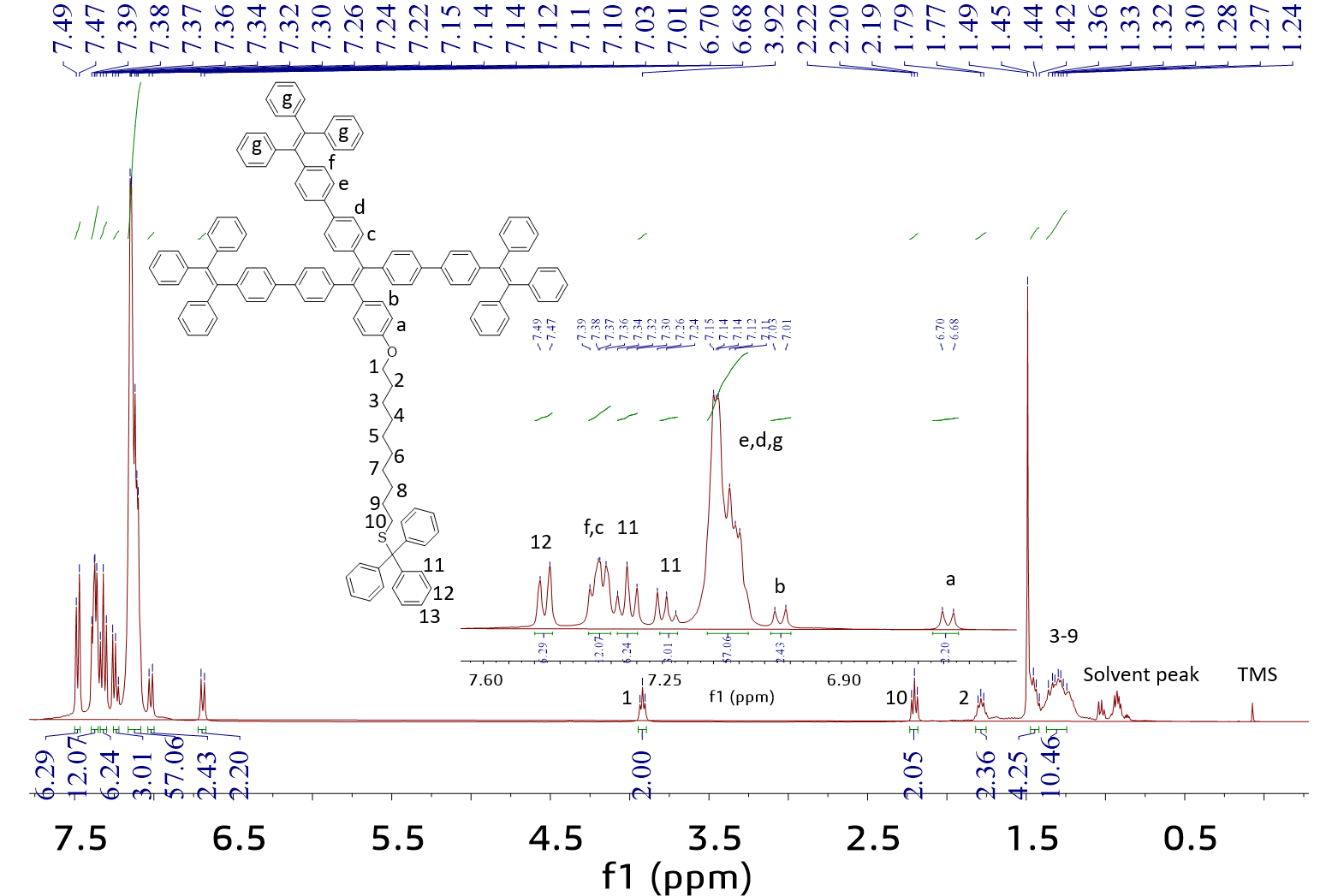


**Supplementary Fig. 53.** **The ^1^H-NMR spectrum of 4TPE-C_10_-STr.**


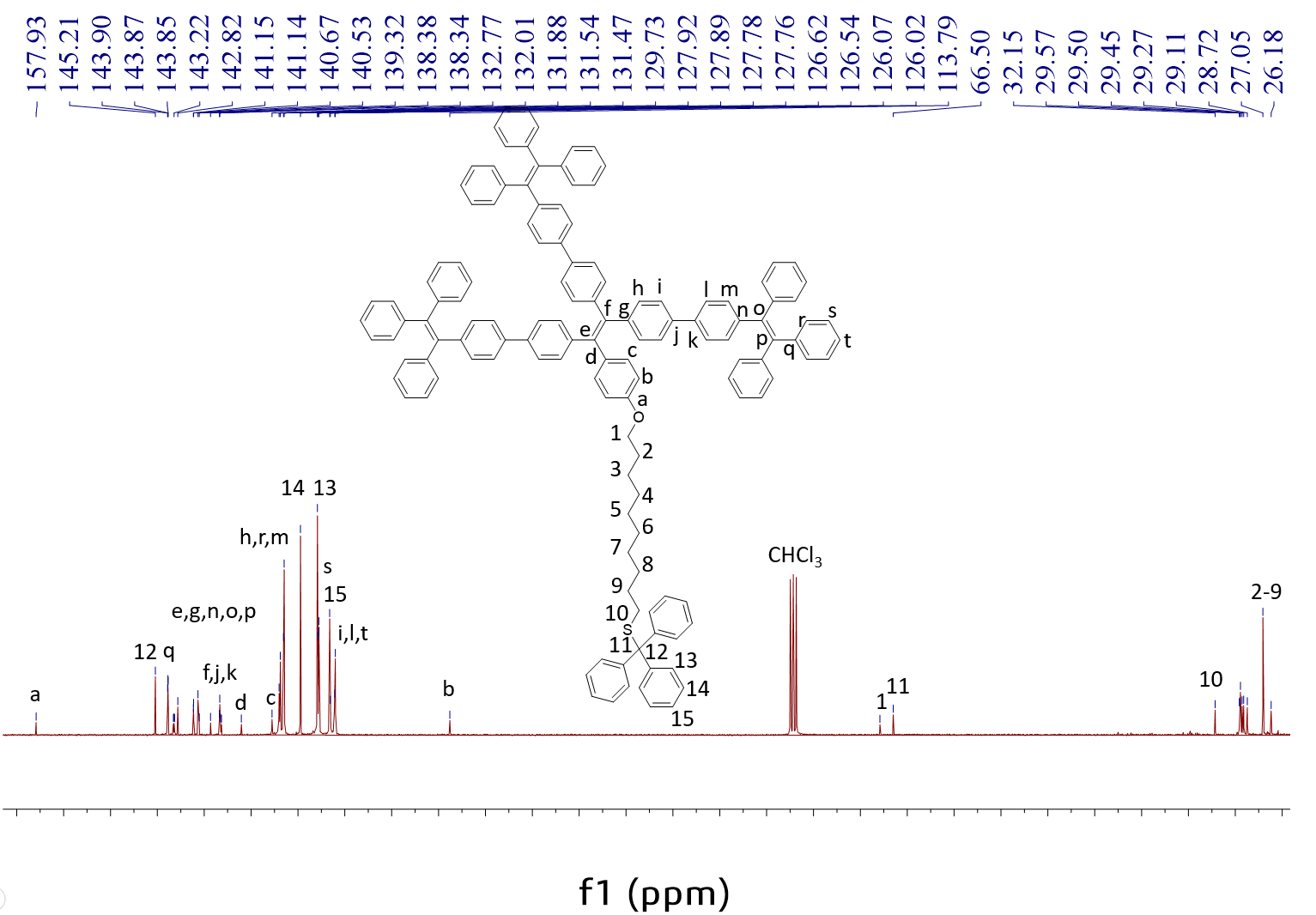


**Supplementary Fig. 54.** **The ^13^C-NMR spectrum of 4TPE-C_10_-STr.**


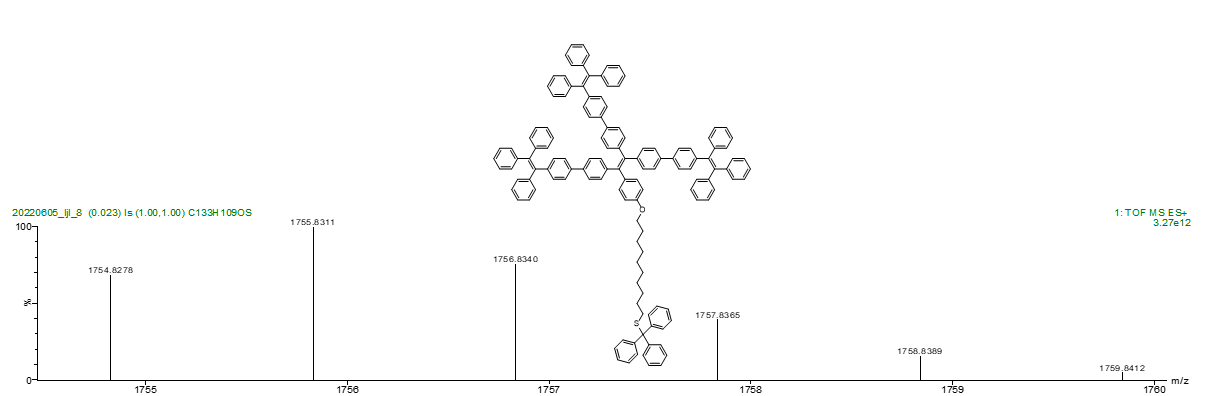


**Supplementary Fig. 55.** **The HRMS spectrum of 4TPE-C_10_-STr.**

**tetra-TPE**

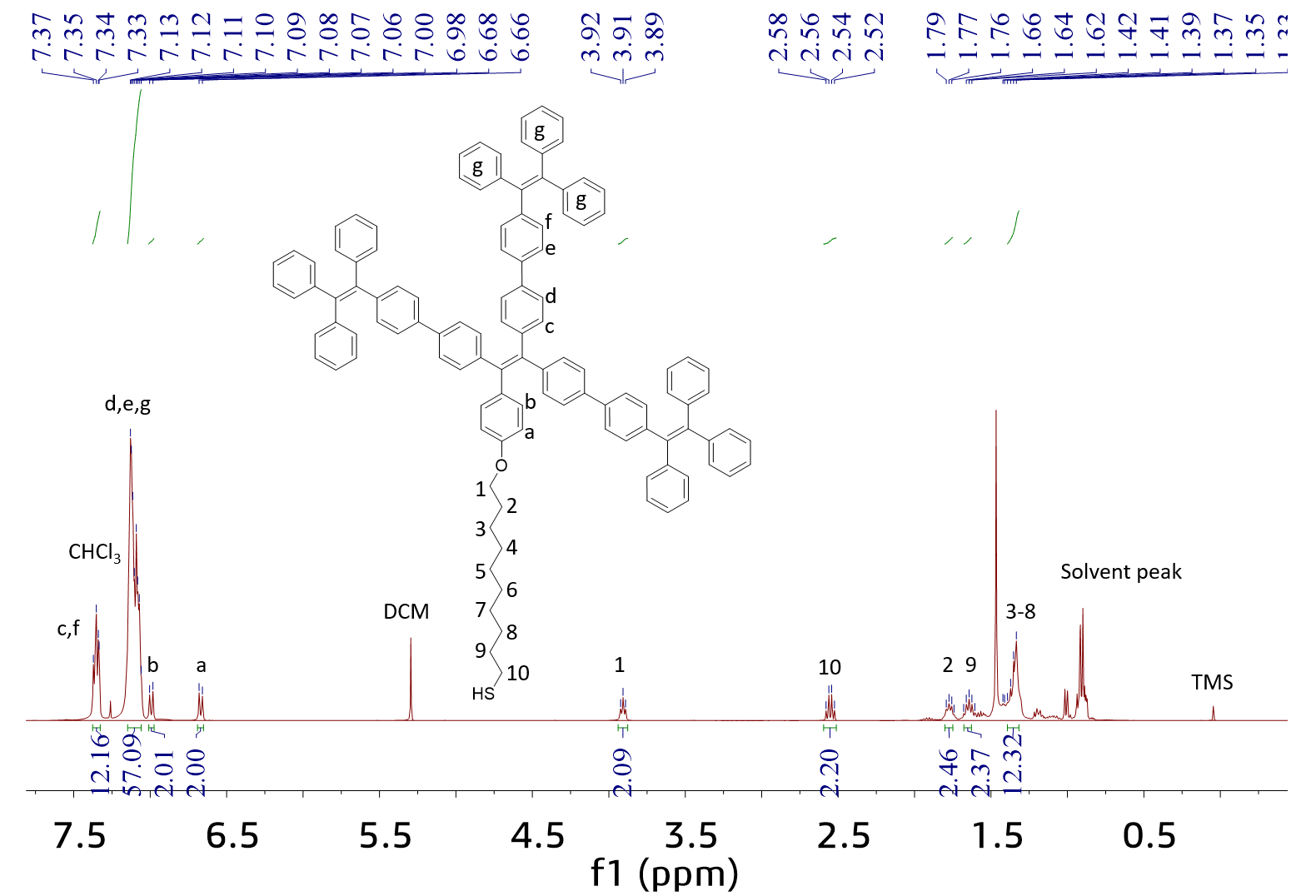


**Supplementary Fig. 56.** **The ^1^H-NMR spectrum of tetra-TPE.**


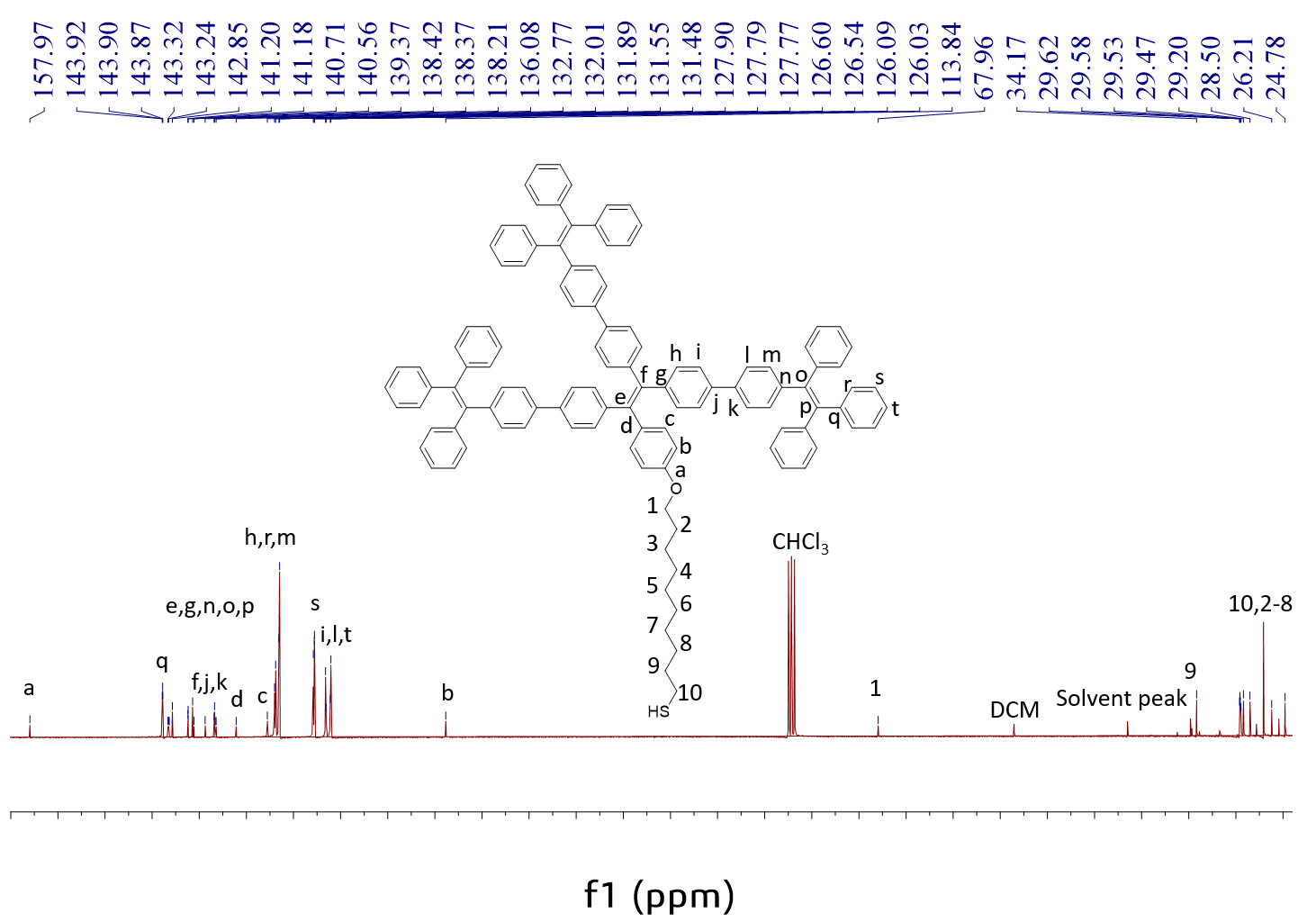


**Supplementary Fig. 57.** **The ^13^C-NMR spectrum of tetra-TPE.**


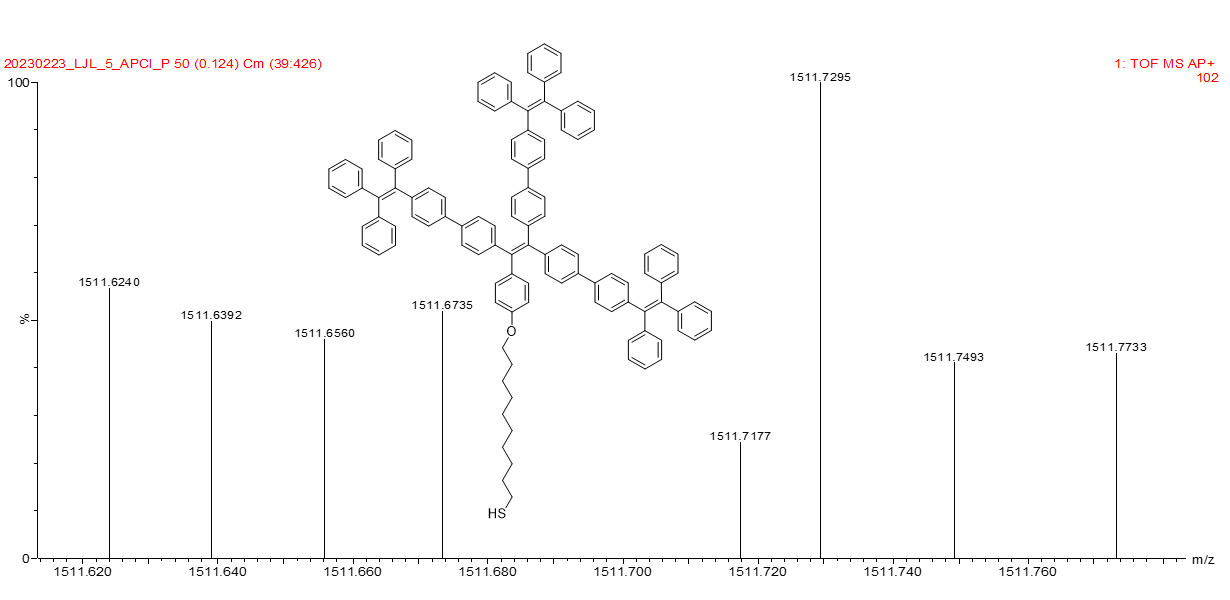


**Supplementary Fig. 58.** **The HRMS spectrum of tetra-TPE.**

**Bending bottom-electrodes**


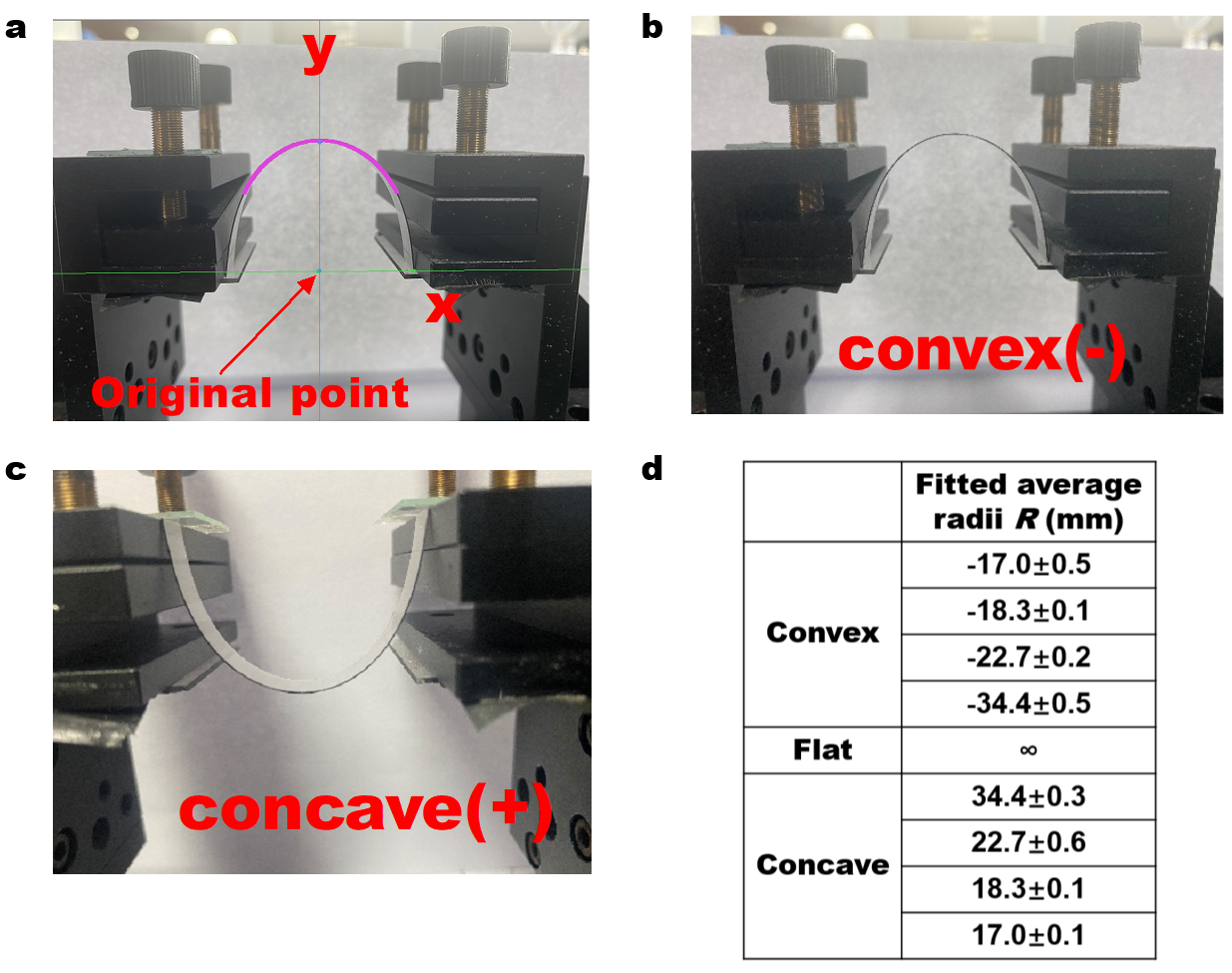


**Supplementary Fig. 59. Determination of radii.** **a, b, c,** Photograph of the junctions created on mechanically bent electrodes. **d,** The results of fitted average radii across the range of electrode shapes, extracted from the data in Supplementary Figs. 61-62. Error bar is calculated from three values from Supplementary Figs. 60-61.


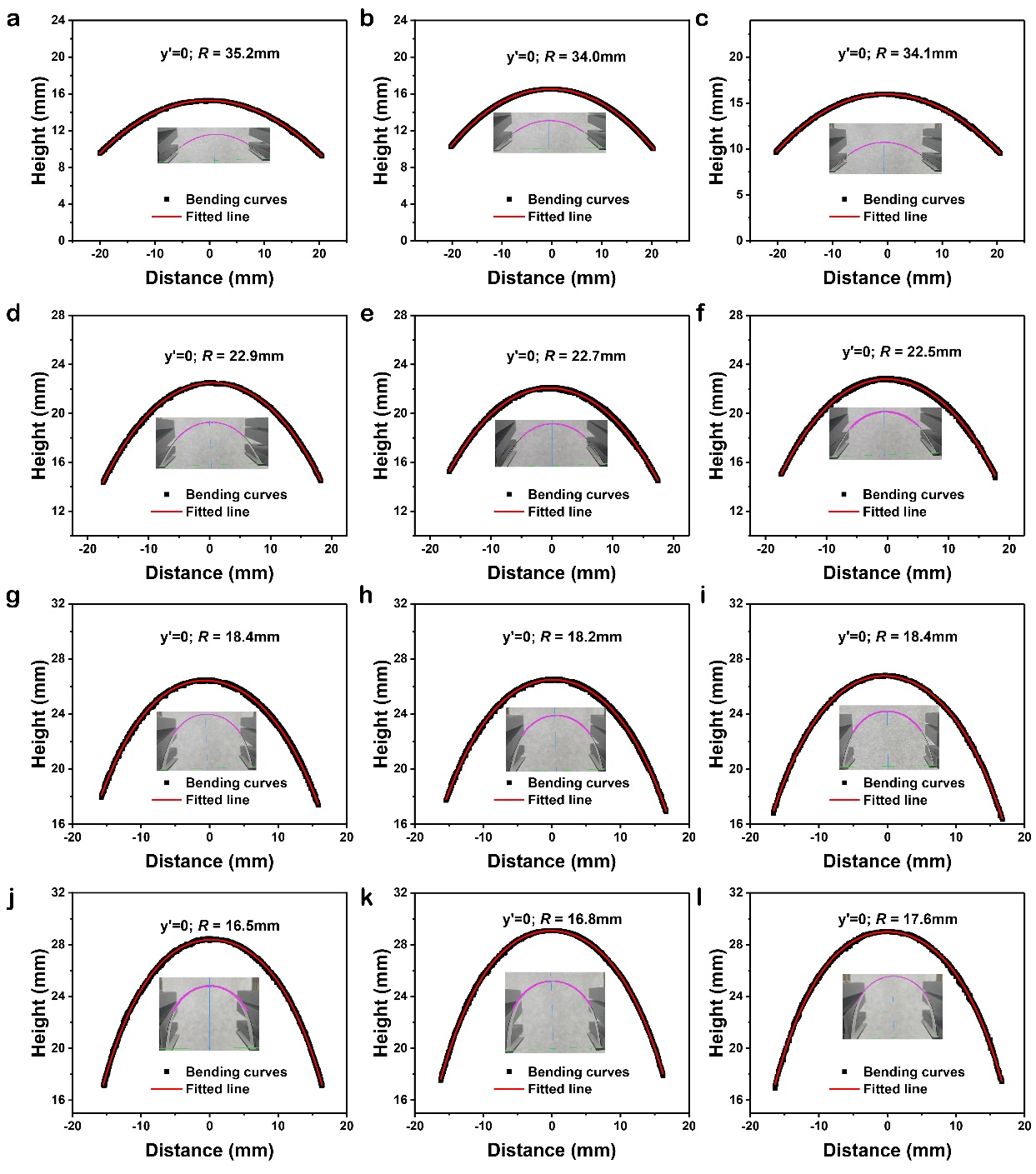


**Supplementary Fig. 60. Radii calculation of convex bending.** The real bending curves (inset) and fitted mathematic curves (red line) at various convex bending. We measured four different extents of convex bending and each *R* was fitted three times. Supplementary Fig. 59d shows the average *R* results. The convex *R* = -34.4±0.5 mm was fitted from **a**, **b** and **c**. *R* = -22.7±0.2 mm was fitted from **d**, **e** and **f**. *R* = -18.3±0.1 mm was fitted from **g**, **h** and **i**. *R* = -17.0±0.5 mm was fitted from **j**, **k** and **l**.


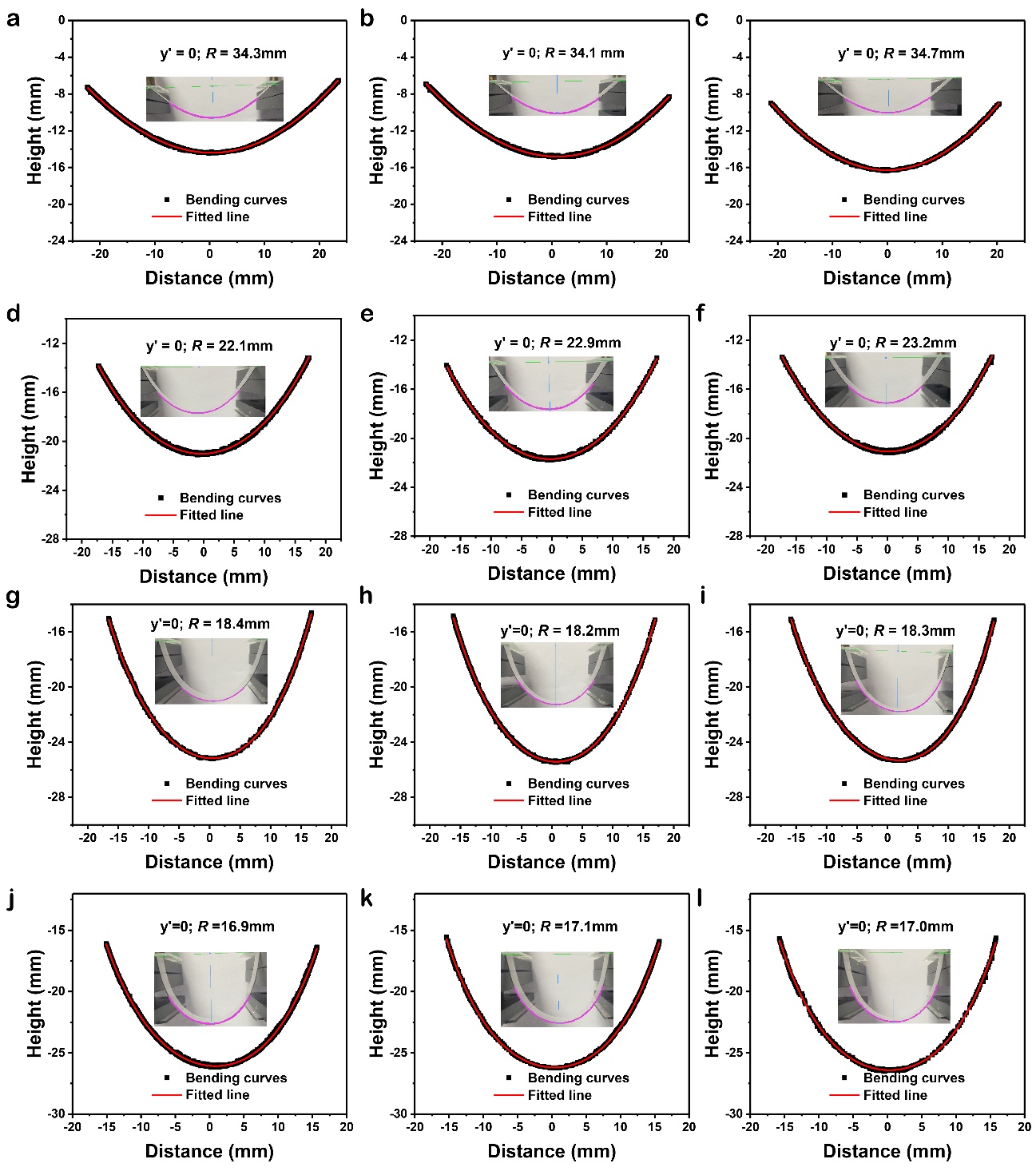


**Supplementary Fig. 61.** **Radii calculation of concave bending.** The real bending curves (inset) and fitted mathematic curves (red line) at various concave bending angles;. We measured four different extents of concave bending and each *R* was fittled three times. Supplementary Fig. 59d shows the average *R* results. The concave *R* = 34.4±0.3 mm was fitted from **a**, **b** and **c**. *R* = 22.7±0.6 mm was fitted from **d**, **e** and **f**. *R* = 18.3±0.1 mm was fitted from **g**, **h** and **i**. *R* = 17.0±0.1 mm was fitted from **j**, **k** and **l**.

**Electronic measurements**


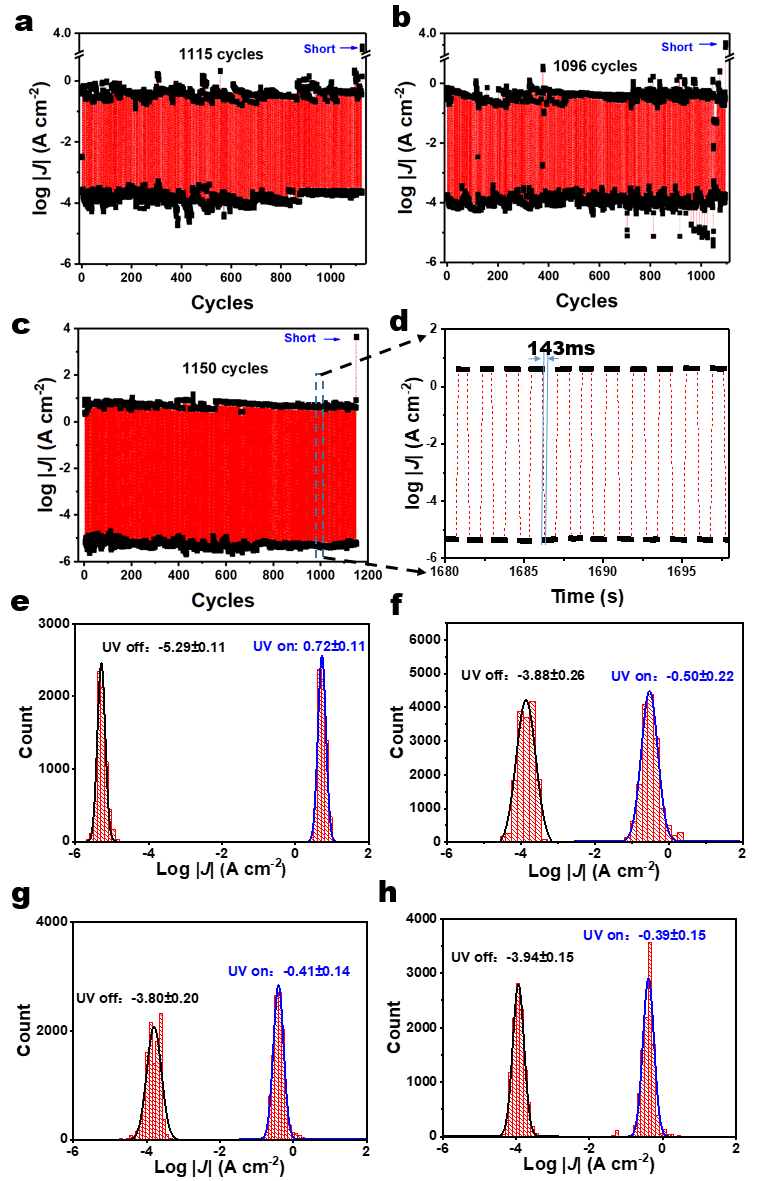


**Supplementary Fig. 62. Log*J(V)* curves and Log*J* histogram.** The real-time on-off cycles of Au-SC_10_-O-TPE at *R* = 17.0 mm with **a,** 1115 and **b,** 1096 cycles. **c**, The real-time on-off cycles of Au-SC_10_-O-tetraTPE at *R* = 17.0 mm with 1150 cycles. **d**, Zoom-in of sustained switching *vs.* time (the full dataset is in panel c) over ten consecutive cycles with UV blinking on and off. The black data points represent the log|*J*| at -1.0 V (*R* = 17.0 mm), and the dashed lines are a guide to the eye. The average switching time is 143 ±13 ms obtained from the >1000 cycles in panel c. **e**, Corresponding histogram of the data in panel c. **f**, Histogram of the data in Fig. 1d. **g**, Histogram of the data in panel a. **(a)**. **h**, Histogram of the data in panel b.


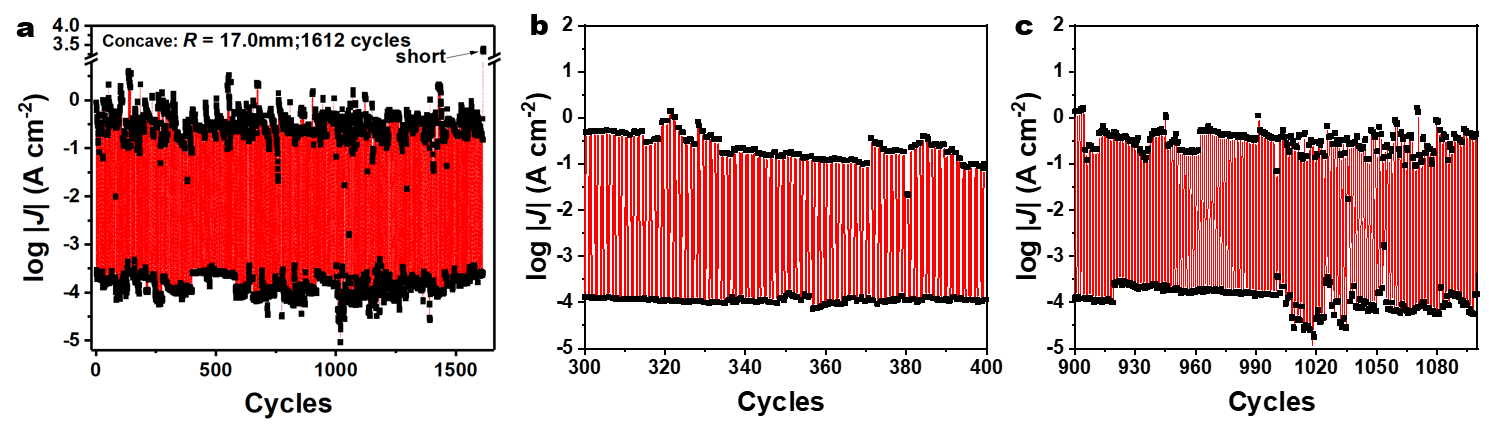


**Supplementary Fig. 63. On-off switching of Au-SC_10_-O-TPE.** **a**, The real-time on-off cycles of Au-SC_10_-O-TPE at *R* = 17.0 mm during 1612 cycles. Zoom-in of **b**, ~100 and **c**, ~195 sustained switching cycles.


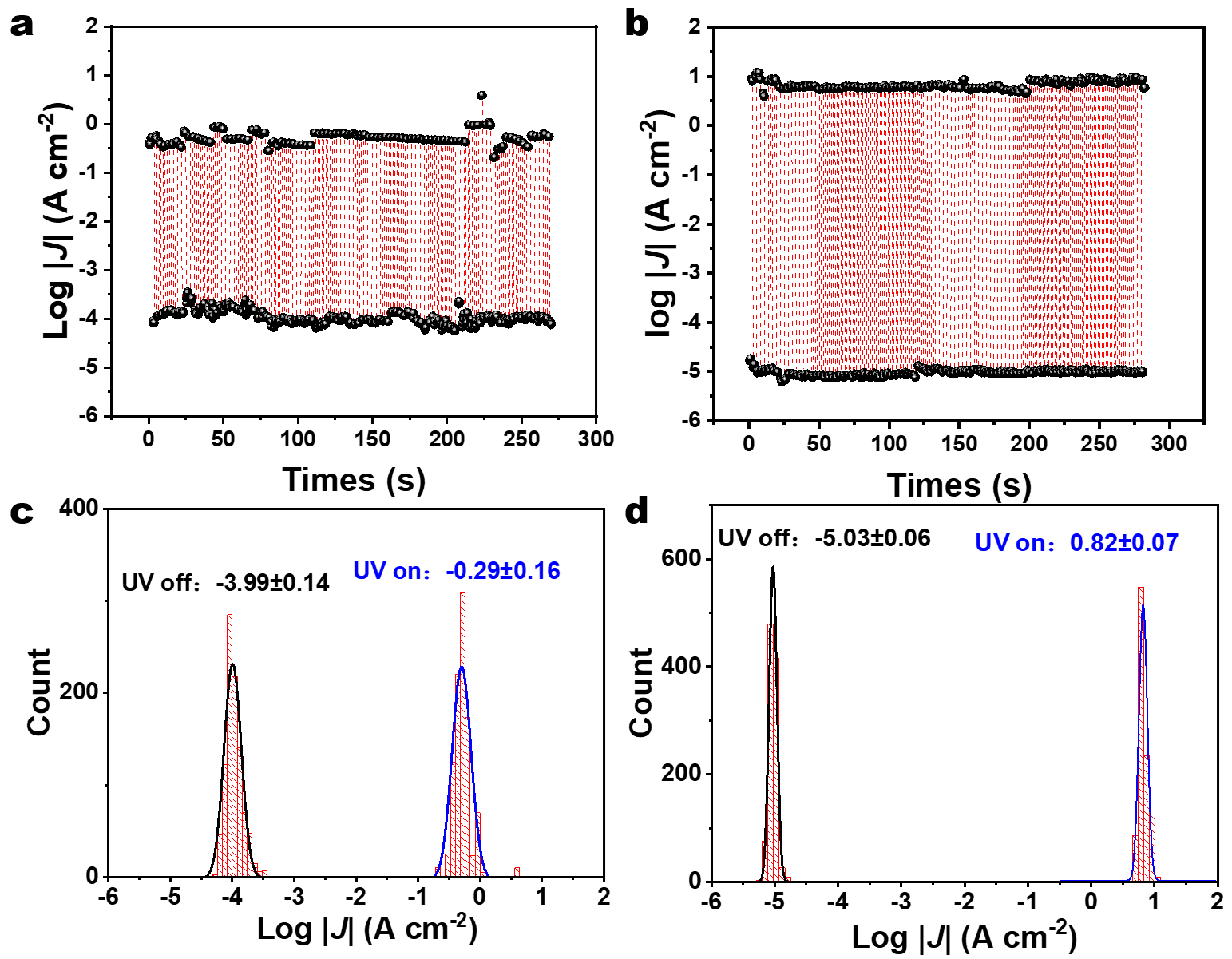


**Supplementary Fig. 64. On-off switching and related histogram.** **a**, The real-time on-off switching of Au-SC_10_-O-TPE at *R* = 17.0 mm. **b**, The real-time on-off switching of Au-SC_10_-O-tetraTPE at *R* = 17.0 mm. **c**, The histogram of data in panel a**. d**, The histogram of data in panel b.


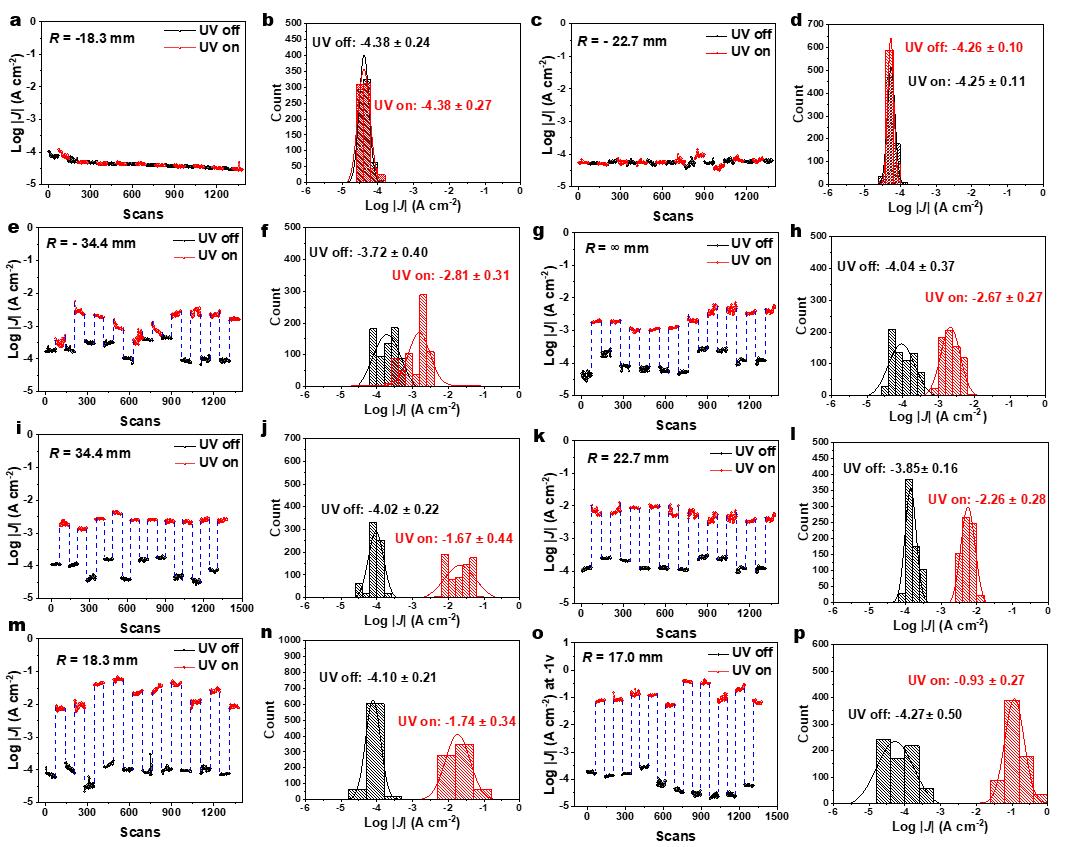


**Supplementary Fig. 65.** **On-off switch cycles under different bending conditions.** The *in-situ* on/off switch cycles of Au-SC_10_-O-TPE SAM at bending radii of **a,** -18.3 mm, **c,** -22.7 mm, **e,** -34.4 mm, **g,** flat, **i,** 34.4 mm, **k,** 22.7 mm, **m,** 18.3 mm and **o,** 17.0 mm. The corresponding histograms for **a**, **c**, **e**, **g**, **i**, **k**, **m**, and **o**, are plotted in panels **b**, **d**, **f**, **h**, **j**, **l**, **n**, and **p,** respectively.


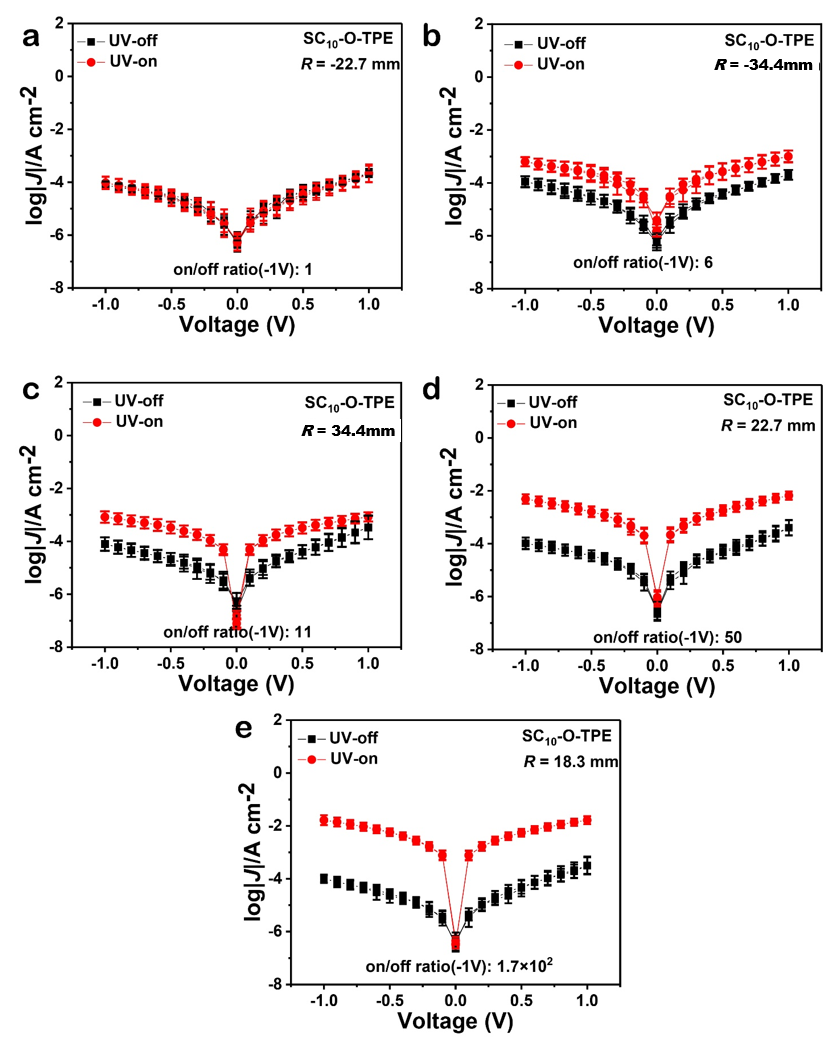


**Supplementary Fig. 66. Log*J(V)* curves of Au-SC_10_-O-TPE.** Log*J(V)* curves of Au-SC_10_-O-TPE at bending radii **a,** *R* = - 22.7 mm, **b,** *R* = - 33.4 mm **c,** *R* = 33.4 mm **d,** *R* = 22.7 mm and **e,** *R* = 18.3 mm .


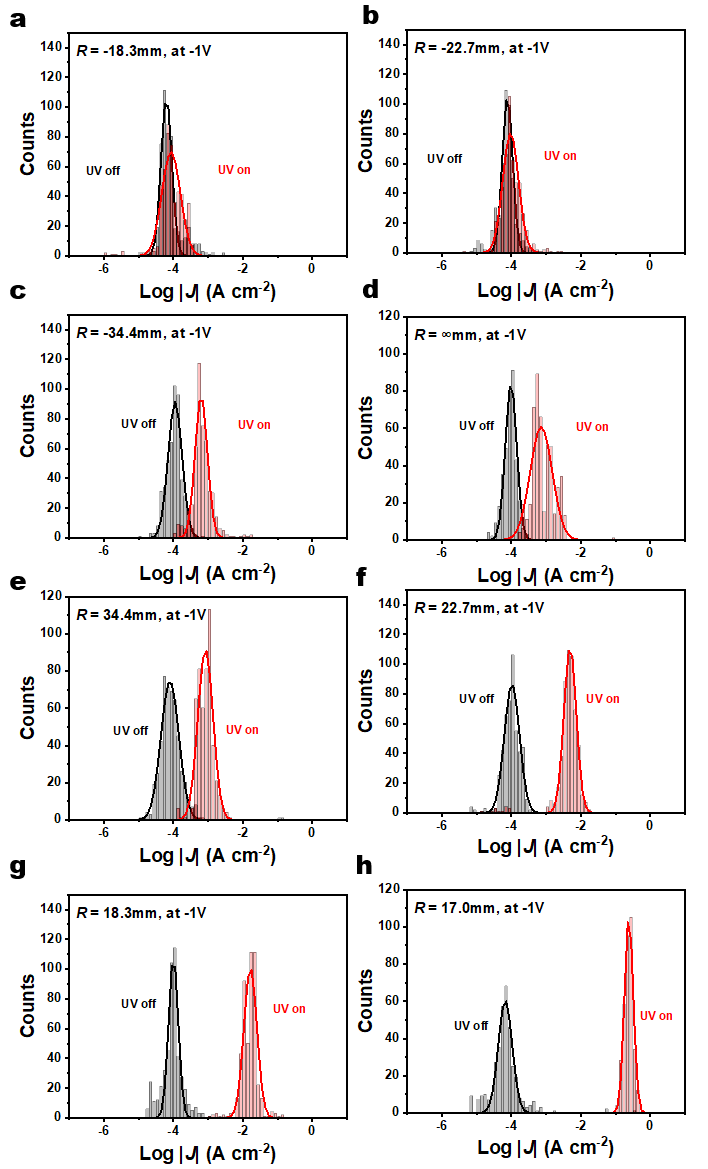


**Supplementary Fig. 67.** **Log*J* histogram of Au-SC_10_-O-TPE**. Histogram of Au-SC_10_-O-TPE bending at **a,** *R* = -18.3 mm, **b,** *R* = -22.7 mm, **c,** *R* = -33.4 mm, **d,** *R* = ∞ mm, **e,** *R* = 33.4 mm, **f,** *R* = 22.7 mm, **g,** *R* = 18.3 mm and **h,** *R* = 17.0 mm.


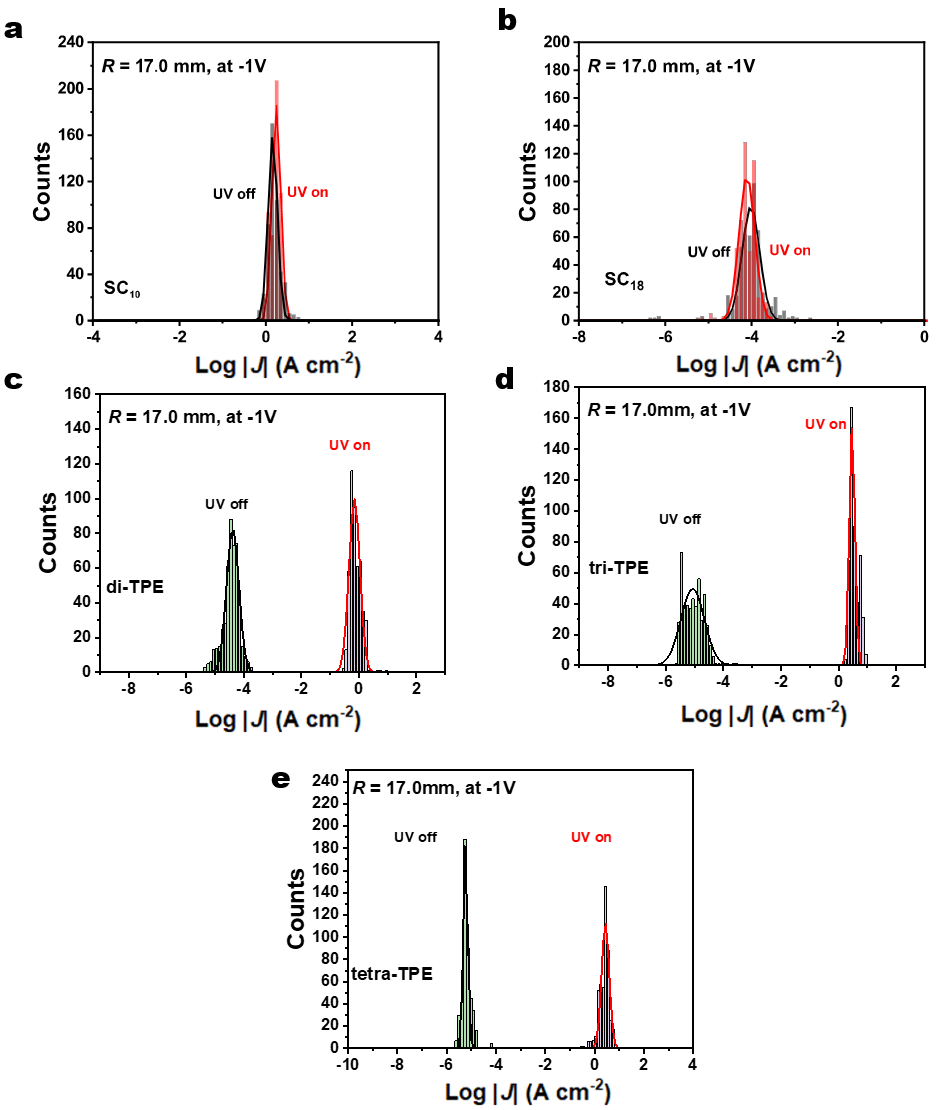


**Supplementary Fig. 68. Log*J* histogram of SC_10_, SC_18_, di-TPE, tri-TPE, and tetra- TPE.** Histogram of **a,** SC_10_, **b,** SC_18_ , **c**,di-TPE, **d**,tri-TPE, **and e**,tetra-TPE SAM ar *R* =17.0 mm.


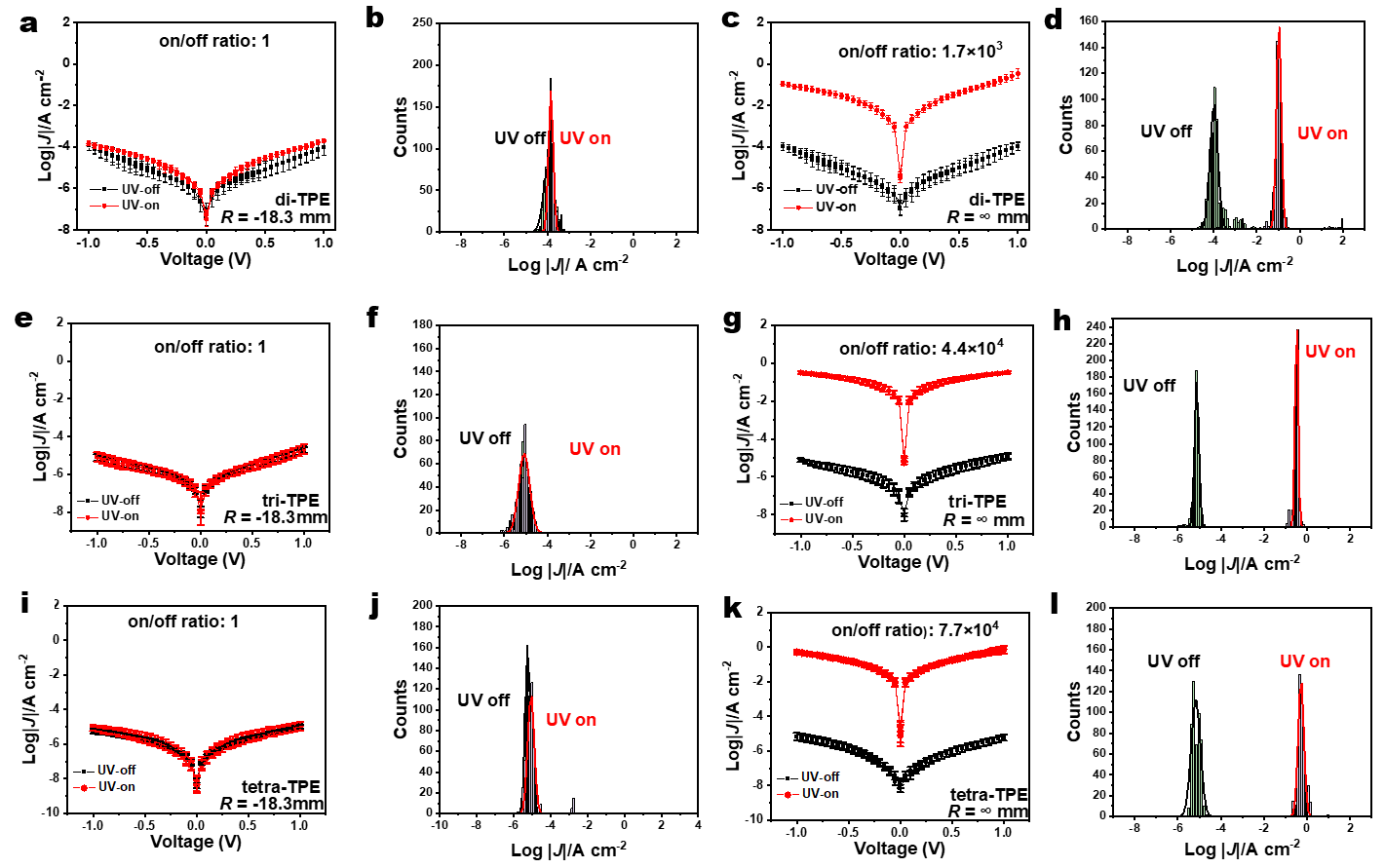
**Supplementary Fig. 69.** **a**, **Log*J(V)* curves and Log*J* histogram of multi-TPE.** Log*J(V)* curves of di-TPE at *R* = -18.3mm. **b,** Histogram of di-TPE at *R* = -18.3 mm. **c**, Log*J(V)* curves of di-TPE at *R* = ∞ mm. **d,** Histogram of di-TPE at *R* = ∞ mm. **e**, Log*J(V)* curves of tri-TPE at *R* = -18.3mm. **f,** Histogram of tri-TPE at *R* = -18.3 mm. **g**, Log*J(V)* curves of tri-TPE at *R* = ∞ mm. **h,** Histogram of tri-TPE at *R* = ∞ mm. **i**, Log*J(V)* curves of tetra-TPE at *R* = -18.3mm. **j,** Histogram of tetra-TPE at *R* = -18.3 mm. **k**, Log*J(V)* curves of tetra-TPE at *R* = ∞ mm. **l,** Histogram of tetra-TPE at *R* = ∞ mm.


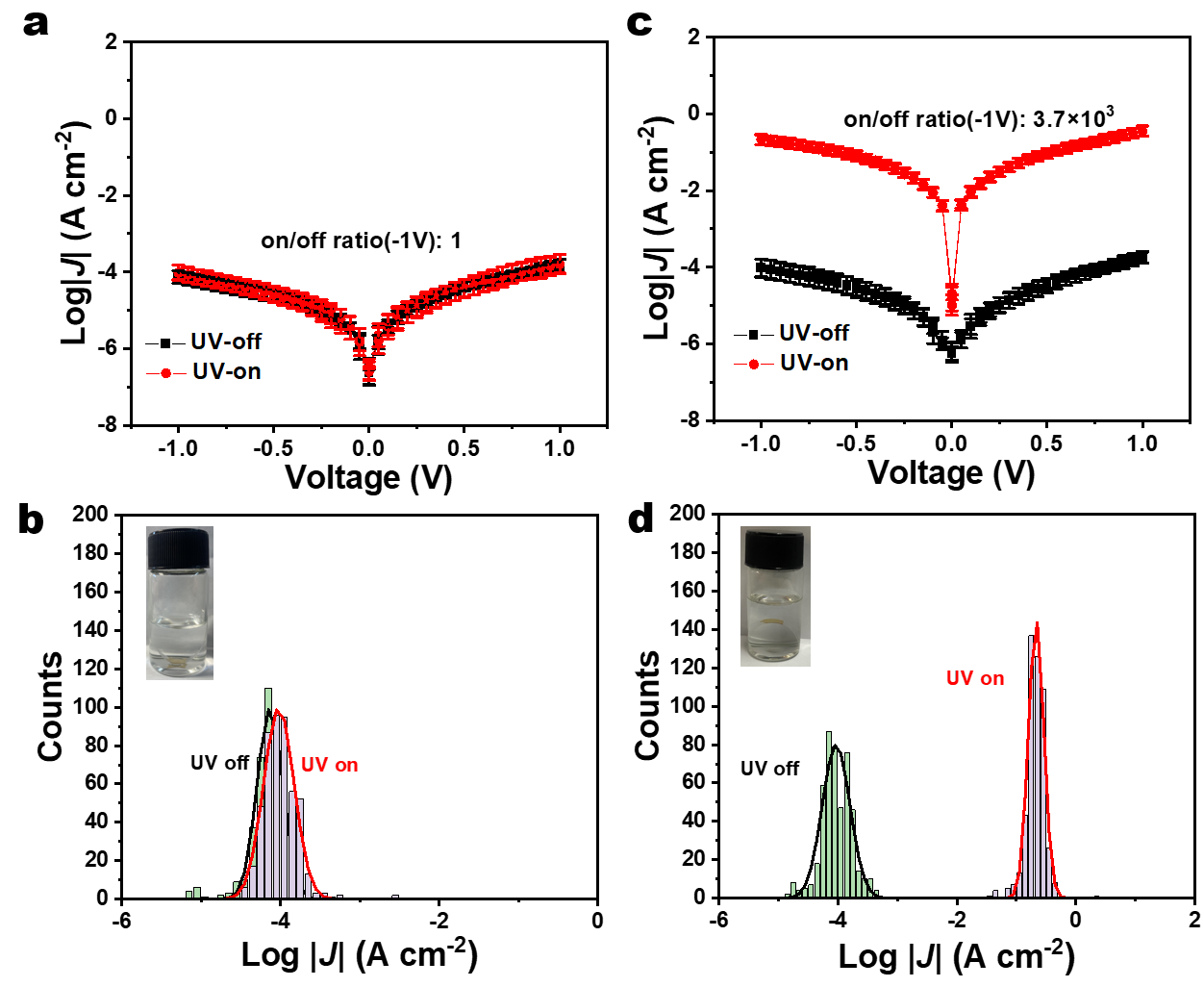


**Supplementary Fig. 70. Log*J(V)* curves and Log*J* histogram of concave and convex growth of mono-TPE.** SAMs grown on concavely bent substrates, and then bent and measured in convex geometry: **a**, The logarithm of the absolute value of the current density (log|*J*|) versus the voltage (V), and **b**, the corresponding histograms of log|*J*| at -1.0 V. SAMs grown on convexly bent substrates, and then bent and measured in concave geometry: **c**, The logarithm of the absolute value of the current density (log|*J*|) versus the voltage (V), and **d**, the corresponding histograms of log|*J*| at -1.0 V.


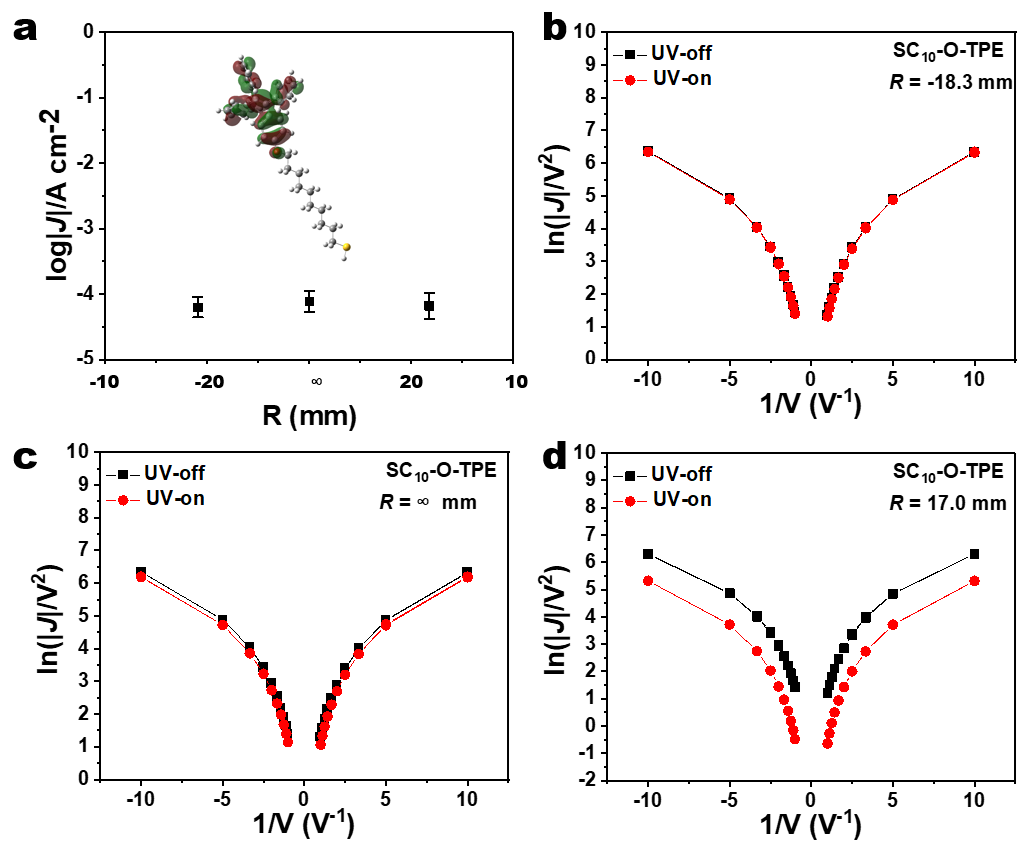


**Supplementary Fig. 71.** **Fowler−Nordheim plots. a**, Log |*J*| as a function of curvature in the dark; Inset is the computed TPE-SAMs molecular HOMO surface (see DFT methods section for details). **b-d**, Fowler−Nordheim (FN) plots for Au-SC_10_-O-TPE, derived from the current-density data in Fig. 2a-c at different bending radii as shown**.**


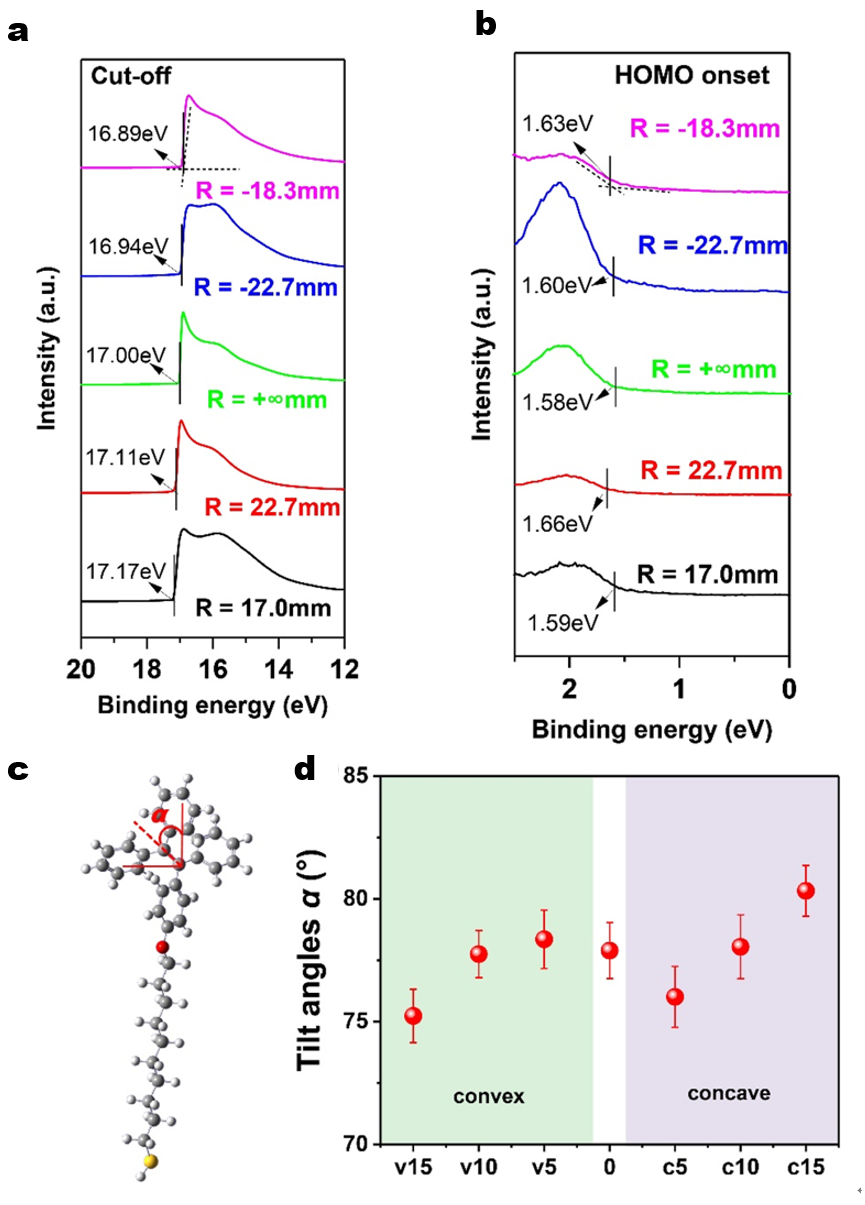


**Supplementary Fig. 72.** **UPS spectrum and tilt angles plot.** UPS of Au-SC_10_-O-TPE at different bending condition. **a,** Cut-off edge and **b,** HOMO onset edge of the UPS spectrum. **c,** 3D model of HSC_10_-O-TPE molecule. The tilt angle *α* is defined as the angle of average π* of the molecule against the surface normal. **d,** The plot of tilt angles *α* predicted from the molecular dynamics models (see Supplementary Fig. 74 below).


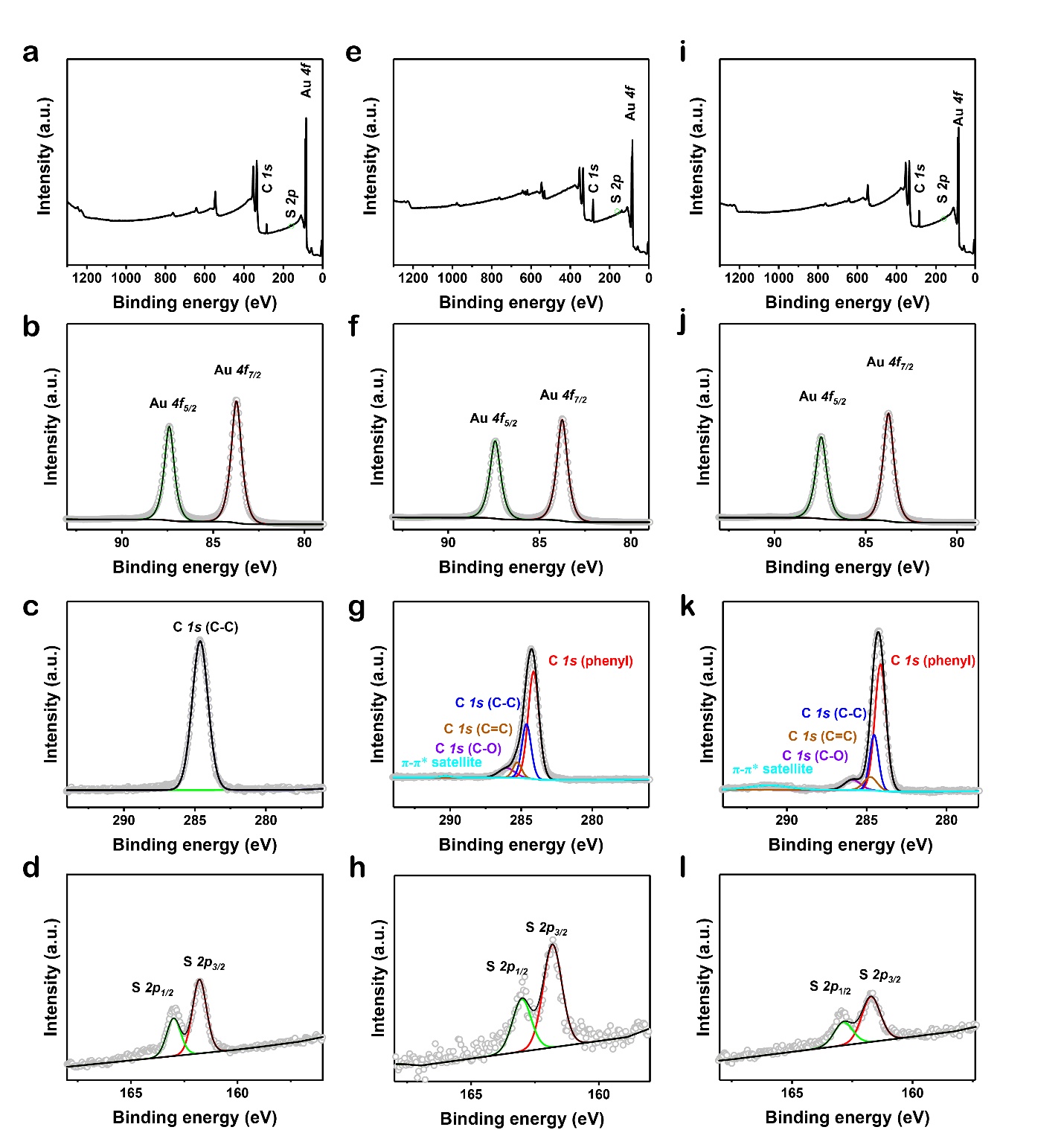


**Supplementary Fig. 73.** **XPS spectrum.** XPS of Au-SC_10_ at flat: **a,** survey; **b,** Au spectrum; **c,** C spectrum and **d,** S spectrum. XPS of Au-SC_10_-O-TPE at -18.3 mm convex bending: **e,** survey; **f,** Au spectrum; **g,** C spectrum and **h,** S spectrum. XPS of Au-SC_10_-O-TPE at -22.7 mm convex bending: **i,** survey; **j,** Au spectrum; **k,** C spectrum and **l,** S spectrum. All the spectra were recorded at *θ* = 90°. *θ* is the emission angles that between the emission electrons and the surface.


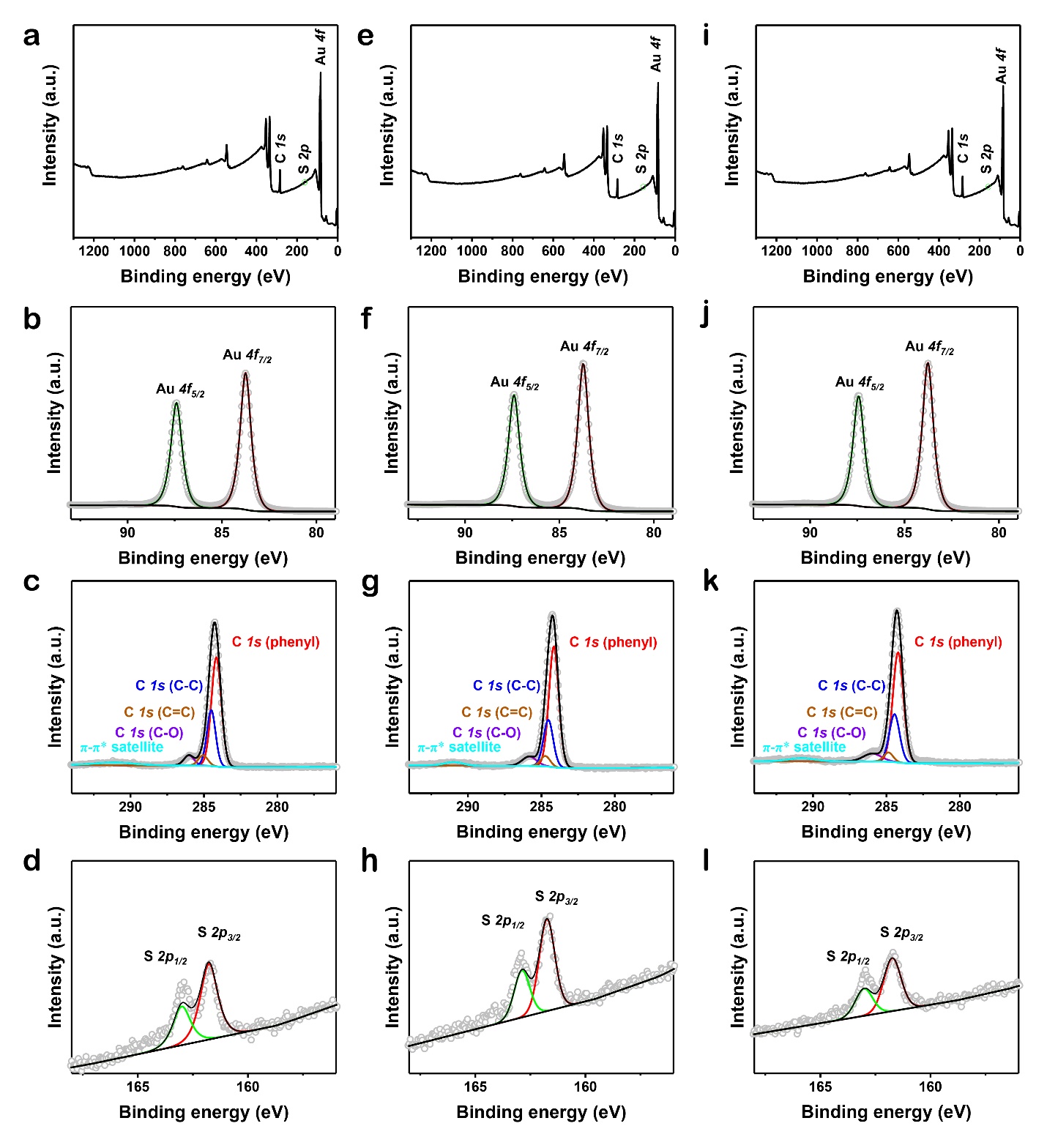


**Supplementary Fig. 74. XPS spectrum.** XPS of Au-SC_10_-O-TPE at flat: **a,** survey; **b,** Au spectrum; **c,** C spectrum and **d,** S spectrum. XPS of Au-SC_10_-O-TPE at 22.7 mm concave bending: **e,** survey; **f,** Au spectrum; **g,** C spectrum and **h,** S spectrum. XPS of Au-SC_10_-O-TPE at 17.0 mm concave bending: **i,** survey; **j,** Au spectrum; **k,** C spectrum and **l,** S spectrum.

**Simulation and calculation**


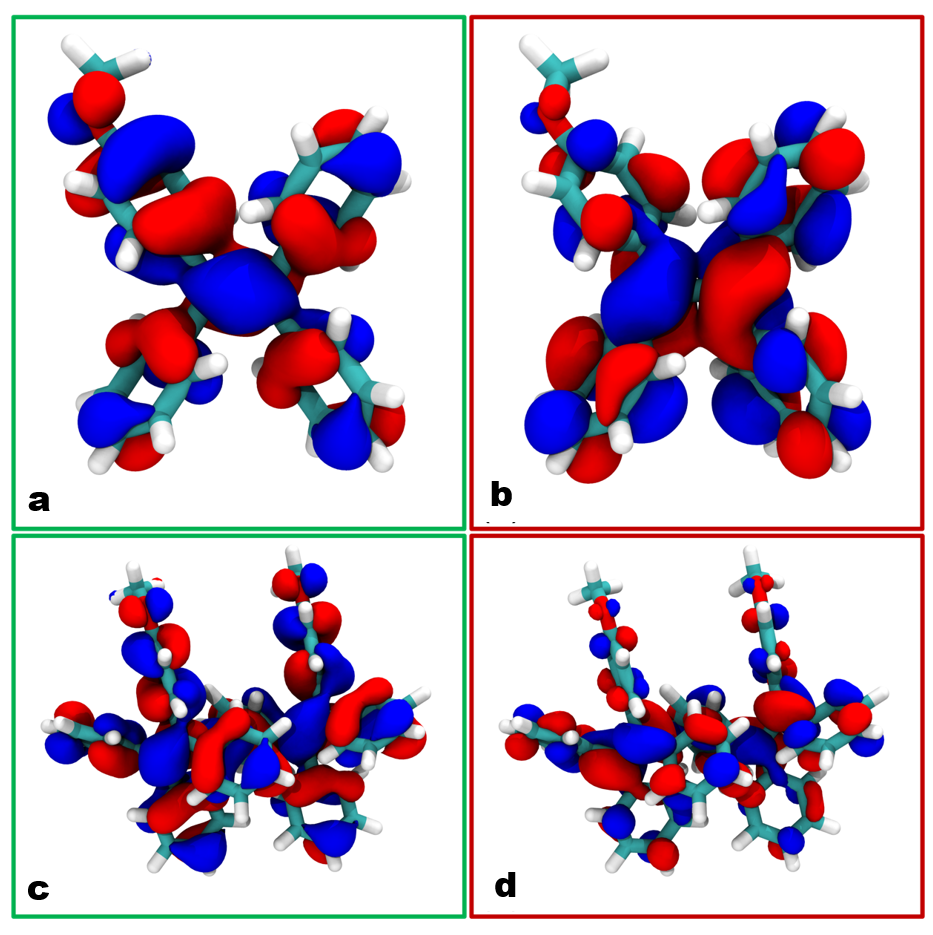


**Supplementary Fig. 75. Molecular orbitals.** Molecular orbitals computed from DFT calculations of the AIE monomer **a, b** and of the crystal dimer **c, d**. **a** and **c** depict the HOMO (green boxes) of the monomer and dimer, respectively. **b** and **d** depict the LUMO (red boxes) of the monomer and dimer, respectively.


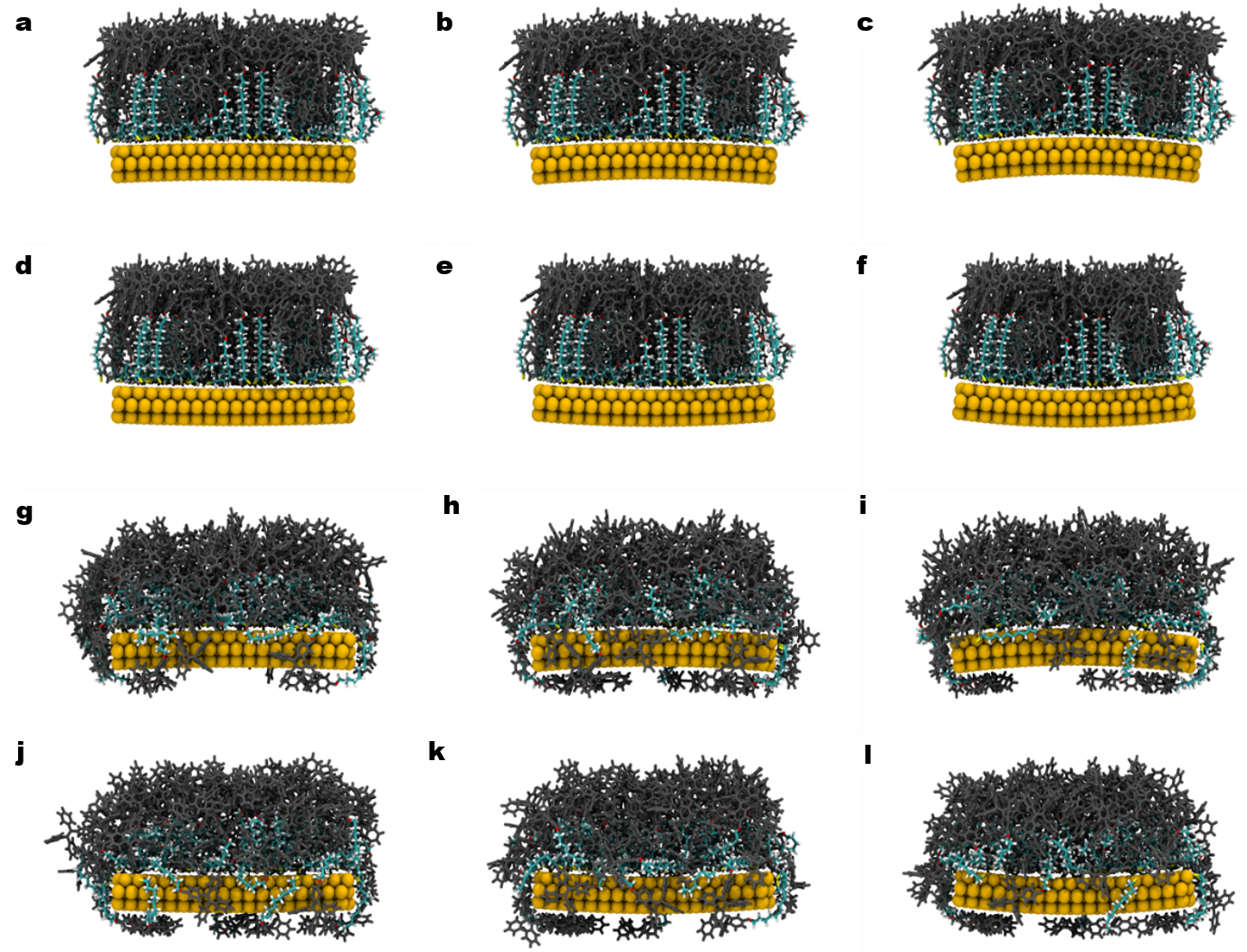


**Supplementary Fig. 76.** **SAM structure from MD simulations.** Computed SAM structures from MD simulations with the supporting gold electrode bent at 5°, 10°, 15° in a convex fashion **(a, b, c, g, h, i)** and in a concave fashion **(d, e, f, g, k, l)**. The starting structures are shown in **a**-**f**. The final structures are shown in **g**-**l**. The gold atoms are shown as van der Waals spheres, the SAM alkyl chains as sticks (Carbon in cyan, Oxygen in red, Sulfur in yellow, Hydrogen in white), and the AIE-active headgroups as grey sticks.


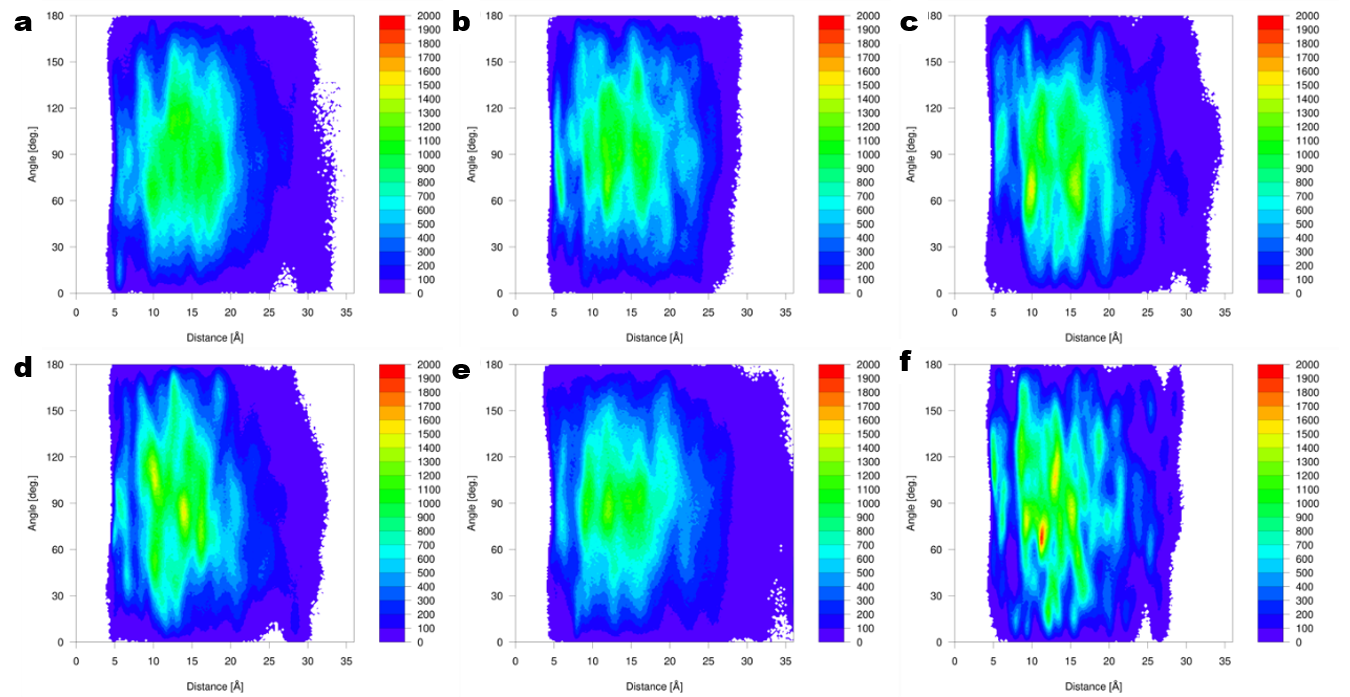


**Supplementary Fig. 77.** **Heat maps of phenyl rings orientation.** Heat maps of phenyl rings orientation vs. inter-molecular separations for the convex (**a**, **b**, and **c**) and the concave SAM geometries (**d**, **e**, and **f**), for 5° (**a**, **d**), 10° (**b**, **e**) and 15° (**d**, **f**) curvatures. The values are computed over 1000 nanoseconds of dynamics for all SAMs and measured between the same central molecule and surrounding patch of 20 molecules to ensure consistency between models and avoid edge effects.


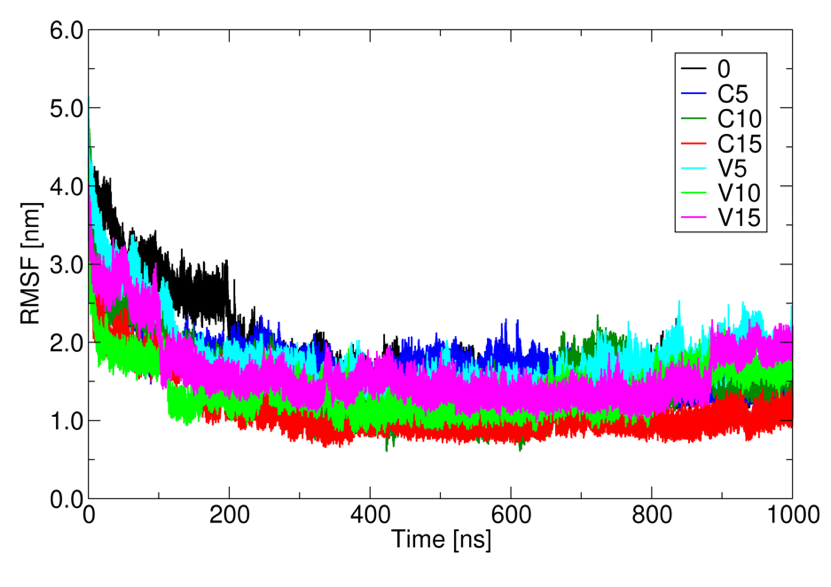


**Supplementary Fig. 78.** **Root mean square fluctuation of SAM.** Computed root mean square fluctuation (RMSF) of the SAM non-hydrogen atoms.


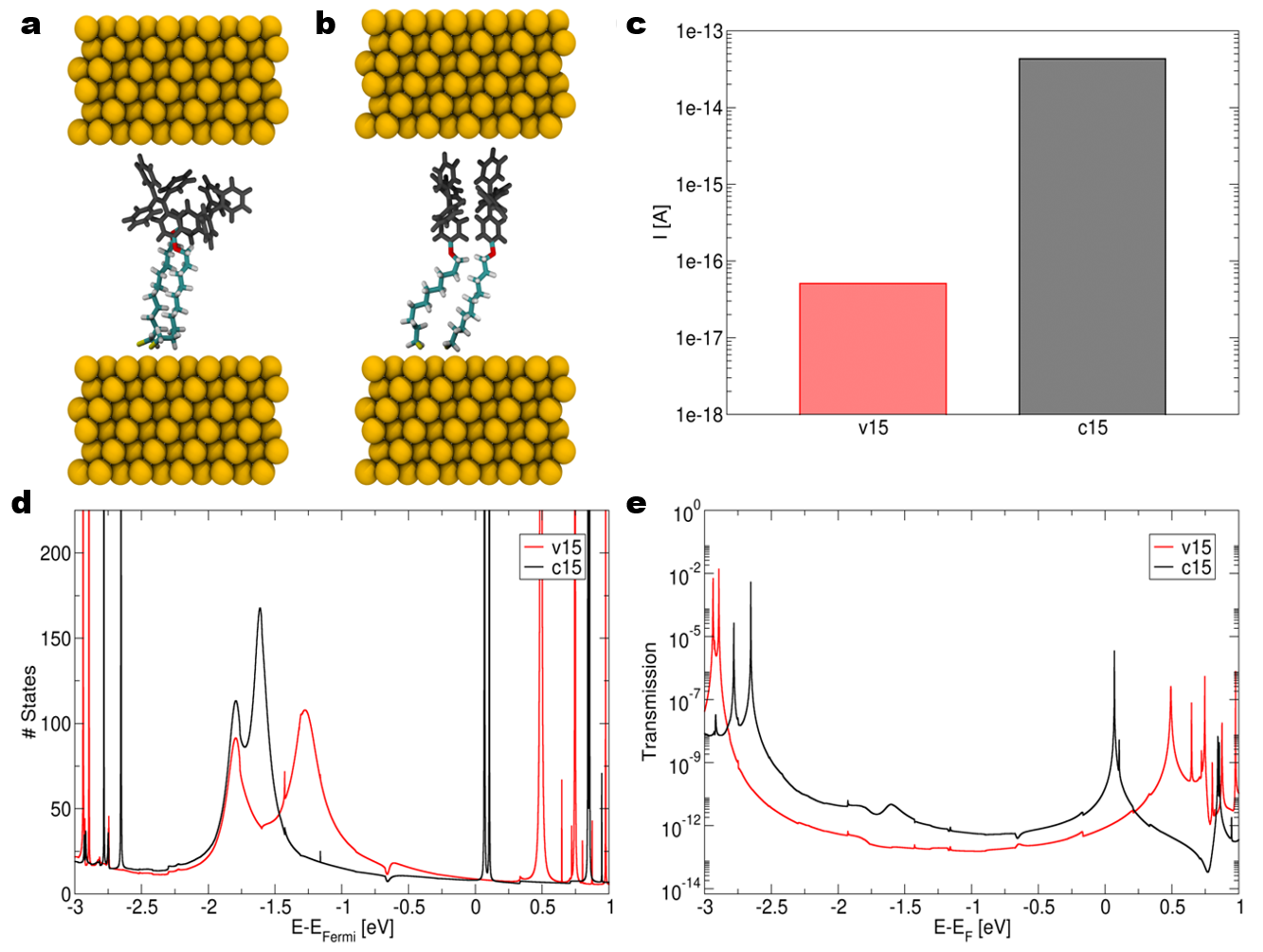


**Supplementary Fig. 79.** **Transport models, density states, and transmission functions.** Representation of transport models consisting of Au-SC_10_-O-TPE dimers from molecular dynamics calculations performed with **a**, a convex bending angle of 15˚, and **b**, a concave bending angle of 15˚. The alkyl chains are shown as colored sticks (Carbon in cyan, Oxygen in red, Sulfur in yellow, Hydrogen in white), the TPE headgroups as grey sticks, and the gold electrode atoms as gold colored spheres. **c**, Computed current in dark conditions (no photoexcitation) for the convex (red bar) and concave (black bar) systems with a biasing potential of 0.5 V. **d**, Density of States (DOS) for the convex (v15) and concave (c15) assemblies during transport at 0.5 V. **e**, Transmission functions for v15 (red) and c15 (black).

**Supplementary Tables**

**Supplementary Table 1.** Measured on/off ratio, HOMO and LUMO energy level, work function, emission peak position, and TPE tilt angles for the Au-SC_10_-O-TPE SAMs at various bending radii.

| Radius *R* (mm) | on/off ratio | HOMO (eV) | WF (eV) | Emission peak (nm) | LUMO (eV) | Tilt angle (°) |
| --- | --- | --- | --- | --- | --- | --- |
| -18.3 ± 0.1 | 1.0 ± 0.4 | -5.94 ± 0.05 | 4.31 ± 0.03 | 464.0 ± 0.3 | -3.27 ± 0.05 | 56 ± 5 |
| -22.7 ± 0.2 | 1.0 ± 0.4 | -5.86 ± 0.04 | 4.26 ± 0.04 | 464.3 ± 0.5 | -3.19 ± 0.04 | 57 ± 5 |
| ∞ | 26.4 ± 4.6 | -5.78 ± 0.03 | 4.20 ± 0.02 | 464.8 ± 0.2 | -3.11 ± 0.03 | 59 ± 5 |
| 22.7 ± 0.6 | 40.0 ± 4.4 | -5.75 ± 0.02 | 4.09 ± 0.03 | 465.6 ± 0.4 | -3.09 ± 0.02 | 60 ± 5 |
| 17.0 ± 0.1 | 3800 ± 100 | -5.62 ± 0.02 | 4.03 ± 0.02 | 466.2 ± 0.3 | -2.96 ± 0.02 | 69 ± 5 |

**Supplementary Table 2.** Comparison of *in-situ* molecular switching

| Photoswitching units | On/off ratio | Switch time | On/off cycles | Ref. |
| --- | --- | --- | --- | --- |
| Diarylethene | (1.1 ± 0.6)$\text{ ×}\text{ }$10^2^ | <100 ms | >1.0$\text{×}\text{ }$10^2^ | 1 |
| Hemicyanine | <100 | <60 s | 50 | 2 |
| mono-TPE | (3.8 $\text{±}\text{ }\text{0.1)}\text{ }\text{×}\text{ }$10^3^ | 140$\text{±}\text{ }$10ms | 1.6$\text{×}\text{ }$10^3^ | This work |
| tetra-TPE | (4.8 $\text{±}\text{ }\text{0.1)}\text{ }\text{×}\text{ }$10^5^ | 143$\text{±}$ 12ms | 1.2$\text{×}\text{ }$10^3^ | This work |

**Supplementary Table 3.** Radius of curvature for MD models

| Angle of Curvature [°] | Radius of Curvature [ nm] |
| --- | --- |
| 5 | 92.53 |
| 10 | 46.26 |
| 15 | 30.84 |

**Supplementary Methods**

**Synthesis**

All commercial reagents and solvents were used as received without further purification unless otherwise mentioned. Column chromatography was performed with silica gel (pore size 60 Å, 200-300 mesh particle size) and thin layer chromatography (TLC) was performed on silica gel with GF254 indicator. All yields given refer to isolated yields unless otherwise noted. Nuclear magnetic resonance (NMR) spectra were recorded on a 400 MHz Bruker spectrometer. Chemical shifts were reported in ppm. Coupling constants (J values) were reported in Hertz. ^1^H NMR chemical shifts were referenced to CHCl_3_ (7.26 ppm). ^13^C NMR chemical shifts were referenced to CDCl_3_ (77.16 ppm). High resolution mass spectra (ESI or APCI) was recorded on a LCMS-IT/TOF (Shimadzu. Japan) mass spectrometer or Waters Xevo G2 Q-TOF mass spectrometer.

**TPE-OH**

Synthesized according to the reference^3^. 1-bromo-1′,2,2′-triphenylethene (8.00 g, 23.9 mmol), 4-hydroxyphenylboronic acid (15.0 g, 50 mmol), potassium carbonate (13.8 g, 100 mmol),toulene (50 mL), water (5 mL) and Pd(PPh_3_)_4_ (50 mg) were added into a 100 mL two necked bottle. After degassing by bubbling with N_2_ for 15 min, the mixture was then heated to reflux for 18 h under N_2_ atmosphere. The mixture was then cooled down to room temperature, the solid was filtered and then solvent was removed by rotary evaporator, the crude product was puriﬁed by column chromatography (silica gel, CH_2_Cl_2_/petroleum ether = 1/1 v/v), TPE-OH was obtained as a white powder in 94% yield (7.83 g). ^1^H NMR (400 MHz, CDCl_3_) δ 7.14 – 6.99 (m, 15H), 6.91 – 6.87 (m, 2H), 6.58 – 6.54 (m, 2H), 4.58 (s, 1H). ^13^C NMR (101 MHz, CDCl_3_) δ 154.13, 144.12, 144.02, 144.01, 140.56, 140.31, 136.49, 132.86, 131.49, 131.47, 131.45, 127.84, 127.74, 126.51, 126.39, 114.72. TOF-MS m/z calc for C_26_H_20_O [M+H]^+^ 349.1592, found 349.1606.

**TPE-C_10_-Br**

Synthesized according to the reference^4^. TPE-OH (1.00 g, 2.87 mmol), 1,10-dibromodecane (4.31 g, 14.4 mmol) and potassium carbonate (0.59 g, 4.31 mmol) were added into a 250 mL round-bottom flask, stirred and refluxed in CH_3_CN (100 mL) under N_2_ atmosphere overnight. The mixture was then cooled down to room temperature, the solid was filtered and then solvent was removed by rotary evaporator, the crude product was puriﬁed by column chromatography (silica gel, petroleum ether to CH_2_Cl_2_/petroleum ether = 1/9 v/v) to yield TPE-C_10_-Br as a white solid (0.98 g, 60%). ^1^H NMR (400 MHz, CDCl_3_) δ 7.16 – 7.00 (m, 15H), 6.93 (d, J = 8.7 Hz, 2H), 6.64 (d, J = 8.7 Hz, 2H), 3.88 (t, J = 6.5 Hz, 2H), 3.42 (t, J = 6.9 Hz, 2H), 1.92 – 1.82 (m, 2H), 1.81 – 1.71 (m, 2H), 1.45 (br, 4H), 1.36 – 1.29 (m, 8H). ^13^C NMR (101 MHz, CDCl_3_) δ 157.81, 144.19, 144.13, 140.73, 140.10, 135.99, 132.60, 131.50, 131.46, 131.44, 127.80, 127.68, 126.43, 126.30, 113.70, 67.88, 34.04, 32.94, 29.54, 29.47, 29.42, 28.86, 28.28, 26.17. TOF-MS m/z calc for C_36_H_39_OBr [M+H]^+^ 567.2263, found 567.2252.

**TPE-C_10_-SAc**

TPE-C_10_-Br (0.82 g, 1.44 mmol) and potassium ethanethioate (0.75 g, 6.53 mmol) were added into a 250 mL flask containing CH_3_CN (100 mL). After degassing by bubbling with N_2_ for 15 min, the mixture was then heated at 85 °C for 12 h under N_2_ atmosphere. The mixture was then cooled down to room temperature, the solid was filtered and then solvent was removed by rotary evaporator. The crude product was puriﬁed by column chromatography (silica gel, CH_2_Cl_2_/petroleum ether = 1/9 v/v). TPE-C_10_-SAc was obtained as a white solid (0.524 g, 65%). ^1^H NMR (400 MHz, CDCl_3_) δ 7.16 – 7.00 (m, 15H), 6.96 – 6.90 (m, 2H), 6.66 – 6.60 (m, 2H), 3.87 (t, J = 6.6 Hz, 2H), 2.87 (t, J = 7.3 Hz, 2H), 2.33 (s, 2H), 1.78 – 1.69 (m, 2H), 1.62 – 1.53 (m, 8H), 1.47 – 1.29 (m, 1H). ^13^C NMR (101 MHz, CDCl_3_) δ 195.95, 157.78, 144.15, 144.10, 140.70, 140.06, 135.95, 132.57, 131.47, 131.43, 131.40, 127.78, 127.66, 126.40, 126.28, 113.68, 67.87, 30.72, 29.59, 29.54, 29.46, 29.45, 29.39, 29.23, 29.16, 28.88, 26.14. TOF-MS m/z calc for C_38_H_42_O_2_S [M+H]^+^ 563.2984, found 563.2952.

**TPE-C_10_-SH**

Synthesized according to the reference^5^. A solution of TPE-C_10_-SAc (563 mg, 1.0 mmol) in 25 mL anhydrous THF was cooled to 0 °C, 2.82 mL LiAlH_4_ (1.0 M in THF) was added dropwise under nitrogen atmosphere. After stirring for additional 30 min at 0 °C, the reaction mixture was warmed to room temperature and stirred for another 1 h. After cooling again to 0 °C, 3N hydrochloric acid was added until the entire solid was dissolved. The organic phase is separated and washed with brine, dried over Na_2_SO_4_, dried over sodium sulfate, filtered, and concentrated using rotary evaporation. The crude product was puriﬁed by column chromatography (CH_2_Cl_2_/petroleum ether = 1/9 v/v), TPE-C_10_-SH was obtained as a white solid (0.313 g, 60%). ^1^H NMR (400 MHz, CDCl_3_) δ 7.12-1.02 (m, 15H), 6.92 (d, J = 8.0 Hz, 2H), 6.62 (d, J = 8.1 Hz, 2H), 3.87 (t, J = 6.4 Hz, 2H), 2.52 (q, J = 7.3 Hz, 2H), 1.77 – 1.69 (m, 2H), 1.66 – 1.56 (m, 2H), 1.45 – 1.28 (m, 12H). ^13^C NMR (101 MHz, CDCl3) δ 157.82, 144.21, 144.16, 140.74, 140.11, 136.01, 132.64, 131.54, 131.51, 131.48, 127.84, 127.72, 126.46, 126.33, 113.71, 67.92, 34.18, 29.62, 29.59, 29.53, 29.45, 29.20, 28.52, 26.20, 24.81. TOF-MS m/z calc for C_36_H_40_OS [M+H]^+^ 521.2878, found 521.2859.

**2Ph-2Bpin-NH**

Synthesized according to the reference^6^. bis(4-bromophenyl)amine (1.0 g, 3.06 mmol), bis(pinacolato) diboron (3.10 g, 12.23 mmol), potassium acetate (1.2 g, 12.23 mmol) and Pd(PPh_3_)_4_ (175 mg, 0.153 mmol) were dissolved in dry 1,4-dioxane (30 mL) in a Schlenk tube, and degassed thoroughly. Then the reaction mixture was allowed to reflux for 16 h under N_2_ atmosphere. After cooling to room temperature, the reaction was quenched with 50 mL water and extracted 3 times with dichloromethane (50 mL × 3). The combined organic layer was dried with anhydrous Na_2_SO_4_, filtered and dried via rotary evaporator. The crude product was purified via column chromatography using ethyl acetate (EA): petroleum ether (1:2 v/v) as eluent to obtain product as a thick colorless gel. (272 mg, 35% Yield). ^1^H NMR (400 MHz, CDCl_3_) δ 7.73 (d, J = 8.4 Hz, 4H), 7.09 (d, J = 8.4 Hz, 4H), 6.04 (s, 1H), 1.34 (s, 24H). ^13^C NMR (101 MHz, CDCl_3_) δ 145.13, 136.37, 116.86, 83.62, 24.97. TOF-MS m/z calc for C_24_H_33_B_2_NO_4_ [M+H]^+^ 420.2747, found 420.2739.

**2TPE-NH**

Synthesized according to the reference^6^.In a 100 mL flask, compound 2Ph-2Bpin-NH (614 mg, 1.46 mmol), 1-bromo-1′,2,2′-triphenylethene (1.2 g, 3.58 mmol) and potassium carbonate (2.02 g, 14.6 mmol), was dissolved in THF (50 mL) with H_2_O (5 mL) added. The mixture was thoroughly degassed and then added Pd(PPh_3_)_4_ (84.4 mg, 0.073 mmol). The reaction was allowed to reflux for 18 h, then cooled down to room temperature and quenched by adding aqueous sodium bicarbonate. The mixture was extracted with DCM and the combined organic layer dried with Na_2_SO_4_, filtered and the solvent was removed via rotary evaporator. The crude product was purified via column chromatography using DCM:PE (1:1 v/v) as eluent to obtain product as white solids. (494 mg, 50% Yield). ^1^H NMR (400 MHz, CDCl_3_) δ 7.15 – 6.99 (m, 30H), 6.88 (d, J = 8.5 Hz, 4H), 6.74 (d, J = 8.3 Hz, 4H), 5.59 (s, 1H). ^13^C NMR (101 MHz, CDCl_3_) δ 144.28, 144.17, 144.10, 141.21, 140.83, 140.06, 136.38, 132.51, 131.59, 131.52, 131.50, 127.84, 127.72, 126.48, 126.38, 126.33, 116.72. TOF-MS m/z calc for C_52_H_39_N [M]^+^ 677.3083, found 677.3070.

**2TPE-C_10_-STr**

Compound 2TPE-NH (300 mg, 0.44 mmol), sodium hydride (60% w/w in grease, 20.6 mg, 0.52 mmol) and dry dimethyl formamide (10mL) was added into a Schlenk tube. Then the reaction mixture was allowed to stir at room temperature for half an hour until all gas has been liberated. Br-C_10_-STr ^5^ (243 mg, 0.49 mmol) was dissolved in 5 mL dry DMF was then added drop-wise on stirring. The reaction mixture was then allowed to heat to 90 °C for 24 h. It was then cooled down to room temperature and added dropwise to 20 mL of cold water where solid suspensions were formed. The solid crude products were filtered out and purified via column chromatography using DCM:PE (1:3 v/v) as eluent to obtain product as pale yellow solids. (144 mg, 30% Yield). ^1^H NMR (400 MHz, CDCl_3_) δ7.51 – 7.49 (m, 6H), 7.35 – 7.32 (m, 6H), 7.28 – 7.24 (m, 3H), 7.20 – 7.08 (m, 30H), 6.94 (d, J = 8.6 Hz, 4H), 6.74 (d, J = 8.6 Hz, 4H), 3.65 – 3.59 (m, 2H), 2.23 (t, J = 7.3 Hz, 2H), 1.61 (br, 2H), 1.47 (dt, J = 14.9, 7.2 Hz, 2H), 1.30 – 1.25 (m, 12H). ^13^C NMR (101 MHz, CDCl_3_) δ 146.15, 145.23, 144.28, 144.16, 144.02, 140.95, 140.03, 136.30, 132.27, 131.56, 131.51, 131.47, 129.73, 127.91, 127.72, 127.69, 126.61, 126.43, 126.32, 126.28, 119.81, 66.52, 52.09, 32.16, 29.62, 29.53, 29.46, 29.26, 29.12, 28.73, 27.57, 27.15. TOF-MS m/z calc for C_81_H_73_NS [M-H]^+^ 1090.5385, found 1090.5386.

**di-TPE**

Synthesized according to the reference^7^. Precursor compound 2TPE-C_10_-STr (500 mg, 0.458 mmol) was deprotected by dissolution in dry CH_2_Cl_2_ (20 mL), followed by addition of triethylsilane (2 mL), trifluoroacetic acid (0.4 mL) was immediately added in one portion, and the resulting solution swirled for 20 min. The reaction was quenched through addition of 20 mL water, the mixture was extracted three times with CH_2_Cl_2_ (3 × 20 mL), the combined organic phases were then dried over Na_2_SO_4_, filtered and concentrated under reduced pressure. The crude product was then purified by column chromatography (CH_2_Cl_2_/petroleum ether = 1/3 v/v) and obtained pure product as a yellow solid (0.241 g, 62%). ^1^H NMR (400 MHz, CDCl_3_) δ 7.12 – 6.99 (m, 30H), 6.85 (d, J = 8.5 Hz, 4H), 6.66 (d, J = 8.5 Hz, 4H), 3.58 – 3.52 (t, J = 15.2, 2H), 2.52 (q, J = 7.4 Hz, 2H), 1.61 (dt, J = 14.8, 7.3 Hz, 2H), 1.43 – 1.25 (m, 14H). ^13^C NMR (101 MHz, CDCl_3_) δ 146.13, 144.30, 144.17, 144.04, 140.93, 140.01, 136.28, 132.27, 131.58, 131.53, 131.49, 127.73, 127.70, 126.45, 126.33, 126.30, 119.79, 52.10, 34.17, 29.67, 29.59, 29.56, 29.19, 28.52, 27.55, 27.16, 24.80. TOF-MS m/z calc for C_62_H_59_NS [M]^+^ 849.4368, found 849.429.

**2Br-TPE-OMe**

Synthesized according to the reference^8^. 4,4'-Dibromobenzophenone (2.57 g, 7.56 mmol), (4-Methoxyphenyl)(phenyl)methanone (1.46 g, 6.87 mmol), and zinc powder (4.49 g, 68.7 mmol) were added to a 250 mL three-necked flask. Nitrogen was pumped for three times and anhydrous tetrahydrofuran (80 mL) was added at 0℃ and stirred for 30 minutes. Titanium tetrachloride (3.8 mL, 34.4 mmol) was added to the reaction small amount for several times, and then heated to reflux for 12 h. The reaction was quenched by adding 10% K_2_CO_3_ solution. The mixture was extracted by ethyl acetate, washed by saturated NaCl solution, and dried by anhydrous Na_2_SO_4_. Compound 2Br-TPE-OMe as white powder was obtained by silica gel chromatography with petroleum ether. (1.07g, yield: 30%). ^1^H NMR (400 MHz, CDCl_3_) δ 7.28 – 7.23 (m, 4H), 7.16 – 7.13 (m, 3H), 7.05 (dd, J = 6.1, 2.6 Hz, 2H), 6.94 (t, J = 8.8 Hz, 4H), 6.89 (d, J = 8.3 Hz, 2H), 6.69 (d, J = 8.6 Hz, 2H), 3.77 (s, 3H). ^13^C NMR (101 MHz, CDCl_3_) δ 158.52, 143.36, 142.62, 142.52, 141.97, 137.54, 135.43, 133.08, 132.54, 131.32, 131.15, 131.04, 127.98, 126.95, 120.61, 113.39, 55.20. TOF-MS m/z calc for C_27_H_20_Br_2_O [M]^+^ 517.9881, found 517.9888.

**2Br-TPE-OH**

Synthesized according to the reference^8^. In a 250 mL three-necked flask, anhydrous CH_2_Cl_2_ was added to dissolve compound 2Br-TPE-OMe (1.58 g, 3.06 mmol). To the solution was dropwise added 1.0 M BBr_3_ in CH_2_Cl_2_ (12 mL, 12 mmol) at -20 °C. After warming to room temperature, the mixture was stirred overnight. Then water (20 mL) was added to the mixture, and it was stirred for additional 1 h. The mixture was extracted with CH_2_Cl_2_, and the organic layer was dried over Na_2_SO_4_. the crude product was puriﬁed by column chromatography (CH_2_Cl_2_/petroleum ether = 1/1 v/v) to yield compound 2Br-TPE-OH was obtained as a white solid (1.39 g, 90%). ^1^H NMR (400 MHz, CDCl_3_) δ 7.26 – 7.21 (m, 4H), 7.16 – 7.11 (m, 3H), 7.02 (dd, J = 6.5, 2.9 Hz, 2H), 6.92 – 6.84 (m, 6H), 6.59 (d, J = 8.6 Hz, 2H), 4.81 (s, 1H). ^13^C NMR (101 MHz, CDCl_3_) δ 154.38, 143.26, 142.55, 142.43, 141.85, 137.68, 135.76, 133.05, 132.74, 131.30, 131.15, 131.06, 128.00, 126.98, 120.65, 114.99, 27.02. TOF-MS m/z calc for C_26_H_18_Br_2_O [M]^+^ 503.9724, found 503.9739.

**2Br-TPE-C_10_-STr**

2Br-TPE-OH (0.51 g, 1.0 mmol), Br-C_10_-STr (0.59 g, 1.2 mmol) and potassium carbonate (0.35 g, 2.5 mmol) were added into a 100 mL round-bottom flask, stirred and refluxed in CH_3_CN (50 mL) under N_2_ atmosphere overnight, then cooled to room temperature. The mixture was then cooled down to room temperature, the solid was filtered and then solvent was removed by rotary evaporator, the crude product was puriﬁed by column chromatography (CH_2_Cl_2_/petroleum ether = 1/4 v/v) to yield 2TPE-C_10_-STr as a white solid (0.60 g, 65%). ^1^H NMR (400 MHz, CDCl_3_) δ 7.44 – 7.39 (m, 6H), 7.27 (dd, J = 10.7, 4.1 Hz, 6H), 7.25 – 7.18 (m, 7H), 7.13 – 7.11 (m, 3H), 7.00 (dd, J = 6.5, 3.0 Hz, 2H), 6.88 (d, J = 8.5 Hz, 4H), 6.85 (d, J = 8.5 Hz, 2H), 6.64 (d, J = 8.8 Hz, 2H), 3.88 (t, J = 6.6 Hz, 2H), 2.14 (t, J = 7.3 Hz, 2H), 1.77 – 1.68 (m, 2H), 1.45 – 1.34 (m, 4H), 1.32 – 1.13 (m, 10H). ^13^C NMR (101 MHz, CDCl_3_) δ 158.21, 145.25, 143.46, 142.73, 142.64, 142.12, 137.49, 135.27, 133.12, 132.55, 131.38, 131.19, 131.08, 129.77, 128.00, 127.93, 126.96, 126.64, 120.61, 113.95, 68.02, 66.53, 32.18, 29.58, 29.50, 29.47, 29.43, 29.29, 29.14, 28.74, 26.18. TOF-MS m/z calc for C_55_H_52_Br_2_OS [M]^+^ 918.2106, found 918.2139.

**3TPE-C_10_-STr**

In a 100 mL flask, compound 2Br-TPE-C_10_-STr (1.34 g, 1.46 mmol), 4,4,5,5-Tetramethyl-2-(4-(1,2,2-triphenylvinyl)phenyl)-1,3,2-dioxaborolane (2.76 g, 6.00 mmol) and potassium carbonate (2.02 g, 14.6 mmol), was dissolved in THF (50 mL) with H_2_O (5 mL) added. The mixture was thoroughly degassed and then added Pd(PPh_3_)_4_ (84.4 mg, 0.073 mmol). The reaction was allowed to reflux for 18 h, then cooled down to room temperature and quenched by adding aqueous sodium bicarbonate. The mixture was extracted with DCM and the combined organic layer dried with Na_2_SO_4_, filtered and the solvent was removed via rotary evaporator. The crude product was purified via column chromatography using DCM:PE (1:3 v/v) as eluent to obtain product as yellow solids. (1.77 g, 85% Yield). ^1^H NMR (400 MHz, CDCl_3_) δ 7.44 – 7.42 (m, 6H), 7.37 – 7.26 (m, 14H), 7.24 – 7.19 (m, 3H), 7.15 – 7.03 (m, 43H), 6.96 (d, J = 8.6 Hz, 2H), 6.65 (d, J = 8.7 Hz, 2H), 3.88 (t, J = 6.4 Hz, 2H), 2.16 (t, J = 7.3 Hz, 2H), 1.78 – 1.70 (m, 2H), 1.42 – 1. 23 (m, 14H)^. 13^C NMR (101 MHz, CDCl_3_) δ 157.93, 145.21, 143.90, 143.87, 143.85, 143.29, 143.22, 143.17, 142.82, 141.15, 141.14, 140.67, 140.53, 139.32, 138.38, 138.34, 138.25, 138.16, 136.04, 132.77, 132.01, 131.88, 131.54, 131.47, 129.73, 127.92, 127.89, 127.78, 127.76, 126.62, 126.54, 126.07, 126.02, 113.79, 67.91, 66.50, 32.15, 29.57, 29.50, 29.45, 29.27, 29.11, 28.72, 27.05, 26.18. TOF-MS m/z calc for C_107_H_90_OS [M+H]^+^ 1423.6791, found 1423.6758.

**tri-TPE**

Precursor compound 3TPE-C_10_-STr (1.42 g, 1.0 mmol) was deprotected by dissolution in dry CH_2_Cl_2_ (30 mL), followed by addition of triethylsilane (3 mL), trifluoroacetic acid (1.0 mL) was immediately added in one portion, and the resulting solution swirled for 30 min. The reaction was quenched through addition of 30 mL water, the mixture was extracted three times with CH_2_Cl_2_ (3 × 30 mL), the combined organic phases were then dried over Na_2_SO_4_, filtered and concentrated under reduced pressure. The crude product was then purified by column chromatography (CH_2_Cl_2_/petroleum ether = 1/3 v/v) and obtained pure product as a yellow solid (0.94 g, 80%). ^1^H NMR (400 MHz, CDCl_3_) δ 7.39 – 7.30 (m, 8H), 7.07 – 7.12 (m, J = 43H), 6.98 (d, J = 8.4 Hz, 2H), 6.67 (d, J = 8.5 Hz, 2H), 3.90 (t, J = 6.5 Hz, 2H), 2.55 (q, J = 7.4 Hz, 2H), 1.82 – 1.73 (m, 2H), 1.64 (dt, J = 14.8, 7.2 Hz, 2H), 1.49 – 1.33 (m, 12H). ^13^C NMR (101 MHz, CDCl_3_) δ 157.91, 144.22, 143.90, 143.87, 143.25, 143.15, 142.84, 142.81, 141.19, 140.97, 140.69, 139.26, 138.42, 138.40, 138.20, 136.09, 132.68, 131.98, 131.97, 131.89, 131.86, 131.53, 131.47, 127.89, 127.79, 127.76, 126.60, 126.54, 126.10, 126.01, 113.81, 67.94, 34.16, 29.60, 29.56, 29.51, 29.45, 29.18, 28.49, 26.19, 24.77. TOF-MS m/z calc for C_88_H_76_OS [M-1]^+^ 1179.5539, found 1179.5547.

**3Br-TPE-OMe**

Synthesized according to the reference^8^. 4,4'-Dibromobenzophenone (2.57 g, 7.56 mmol), (4-Bromophenyl)(4-methoxyphenyl)methanone (2.00 g, 6.87 mmol), and zinc powder (4.49 g, 68.7 mmol) were added to a 250 mL three-necked flask. Nitrogen was pumped for three times and anhydrous tetrahydrofuran (80 mL) was added at 0℃ and stirred for 30 minutes. Titanium tetrachloride (3.8 mL, 34.4 mmol) was added to the reaction small amount for several times, and then heated to reflux for 12 h. The reaction was quenched by adding 10% K_2_CO_3_ solution. The mixture was extracted by ethyl acetate, washed by saturated NaCl solution, and dried by anhydrous Na_2_SO_4_. Compound 3Br-TPE-OMe as white powder was obtained by silica gel chromatography with petroleum ether. (1.49 g, yield: 33%). ^1^H NMR (400 MHz, CDCl_3_) δ 7.22 (dd, J = 8.5, 2.3 Hz, 6H), 6.89 – 6.82 (m, 8H), 6.64 (d, J = 8.7 Hz, 2H), 3.72 (s, 3H). ^13^C NMR (101 MHz, CDCl_3_) δ 158.66, 142.31, 142.10, 140.64, 138.11, 134.89, 132.98, 132.96, 132.51, 131.28, 131.18, 121.03, 120.95, 120.82, 113.52, 55.20. TOF-MS m/z calc for C_27_H_19_Br_3_O [M]^+^ 595.8986, found 595.8959.

**4TPE-OMe**

In a 100 mL flask, compound 3Br-TPE-OMe (874 mg, 1.46 mmol), 4,4,5,5-Tetramethyl-2-(4-(1,2,2-triphenylvinyl)phenyl)-1,3,2-dioxaborolane (3.35 g, 7.30 mmol) and potassium carbonate (2.02 g, 14.6 mmol), was dissolved in THF (50 mL) with H_2_O (5 mL) added. The mixture was thoroughly degassed and then added Pd(PPh_3_)_4_ (84.4 mg, 0.073 mmol). The reaction was allowed to reflux for 18 h, then cooled down to room temperature and quenched by adding aqueous sodium bicarbonate. The mixture was extracted with DCM and the combined organic layer dried with Na_2_SO_4_, filtered and the solvent was removed via rotary evaporator. The crude product was purified via column chromatography using DCM:PE (1:3 v/v) as eluent to obtain product as yellow solids. (1.68 g, 85% Yield). ^1^H NMR (400 MHz, CDCl_3_) δ 7.37 – 7.31 (m, 12H), 7.16 – 7.04 (m, 57H), 6.99 (d, J = 8.6 Hz, 2H), 6.67 (d, J = 8.7 Hz, 2H), 3.76 (s, 3H). ^13^C NMR (101 MHz, CDCl_3_) δ 158.32, 143.91, 143.88, 143.86, 143.25, 143.18, 143.12, 142.85, 141.17, 141.15, 140.68, 140.44, 139.46, 138.38, 138.37, 138.34, 138.30, 138.22, 136.30, 132.79, 132.00, 131.89, 131.55, 131.48, 127.89, 127.79, 127.77, 126.60, 126.54, 126.09, 126.02, 113.29, 55.23. TOF-MS m/z calc for C_105_H_76_O [M]^+^ 1352.5896, found 1352.5911.

**4TPE-OH**

In a 250 mL three-necked flask, anhydrous CH_2_Cl_2_ was added to dissolve compound 4TPE-OMe (4.06 g, 3.00 mmol). To the solution was dropwise added 1.0 M BBr_3_ in CH_2_Cl_2_ (12 mL, 12 mmol) at -20 °C. After warming to room temperature, the mixture was stirred overnight. Then water (20 mL) was added to the mixture, and it was stirred additional 1 h. The mixture was extracted with CH_2_Cl_2_, and the organic layer was dried over Na_2_SO_4_. the crude product was puriﬁed by column chromatography (CH_2_Cl_2_/petroleum ether = 1/1 v/v) to yield compound 3TPE-OH was obtained as a yellow solid (3.62 g, 90%). ^1^H NMR (400 MHz, CDCl_3_) δ 7.35 (br, 12H), 7.17 – 7.03 (m, 57H), 6.95 (d, J = 8.3 Hz, 2H), 6.58 (d, J = 8.5 Hz, 2H), 4.68 – 4.59 (br, 1H). ^13^C NMR (101 MHz, CDCl_3_) δ 154.31, 143.92, 143.89, 143.87, 143.20, 143.07, 142.88, 141.20, 140.70, 140.36, 139.60, 138.47, 138.45, 138.37, 138.34, 133.00, 131.99, 131.91, 131.55, 131.48, 127.91, 127.80, 126.61, 126.14, 126.07, 126.04, 114.88. TOF-MS m/z calc for C_104_H_74_O [M]^+^ 1338.5740, found 1338.5762.

**4TPE-C_10_-STr**

4TPE-OH (1.34 g, 1.0 mmol), Br-C_10_-STr (0.59 g, 1.2 mmol) and potassium carbonate (0.35 g, 2.5 mmol) were added into a 100 mL round-bottom flask, stirred and refluxed in CH_3_CN (50 mL) under N_2_ atmosphere overnight, then cooled to room temperature. The mixture was then cooled down to room temperature, the solid was filtered and then solvent was removed by rotary evaporator, the crude product was puriﬁed by column chromatography (CH_2_Cl_2_/petroleum ether = 1/3 v/v) to yield 4TPE-C_10_-STr as a yellow solid (1.64 g, 70%). ^1^H NMR (400 MHz, CDCl_3_) δ 7.48 (d, J = 7.6 Hz, 6H), 7.39 – 7.36 (m, 12H), 7.32 (t, J = 7.6 Hz, 6H), 7.28 – 7.22 (m, 3H), 7.18 – 7.07 (m, 57H), 7.02 (d, J = 8.6 Hz, 2H), 6.69 (d, J = 8.6 Hz, 2H), 3.92 (t, J = 6.4 Hz, 2H), 2.20 (t, J = 7.3 Hz, 2H), 1.82 – 1.72 (m, 2H), 1.47 – 1.41 (m, 4H), 1.36 – 1.24 (m, 10H). ^13^C NMR (101 MHz, CDCl_3_) δ 157.93, 145.21, 143.90, 143.87, 143.85, 143.29, 143.22, 143.17, 142.82, 141.15, 141.14, 140.67, 140.53, 139.32, 138.38, 138.34, 138.25, 138.16, 136.04, 132.77, 132.01, 131.88, 131.54, 131.47, 129.73, 127.92, 127.89, 127.78, 127.76, 126.62, 126.54, 126.07, 126.02, 113.79, 67.91, 66.50, 32.15, 29.57, 29.50, 29.45, 29.27, 29.11, 28.72, 27.05, 26.18.TOF-MS m/z calc for C_133_H_108_OS [M]^+^ 1754.8277, found 1754.8278.

**tetra-TPE**

Precursor compound 4TPE-C_10_-STr (1.75 g, 1.0 mmol) was deprotected by dissolution in dry CH_2_Cl_2_ (30 mL), followed by addition of triethylsilane (3 mL), trifluoroacetic acid (1.0 mL) was immediately added in one portion, and the resulting solution swirled for 30 min. The reaction was quenched through addition of 30 mL water, the mixture was extracted three times with CH_2_Cl_2_ (3 × 30 mL), the combined organic phases were then dried over Na_2_SO_4_, filtered and concentrated under reduced pressure. The crude product was then purified by column chromatography (CH_2_Cl_2_/petroleum ether = 1/2 v/v) and obtained pure product as a yellow solid (1.18 g, 78%). ^1^H NMR (400 MHz, CDCl_3_) δ7.33 – 7.37(m 12H), 7.00 – 7.13 (m, 57H), 6.99 (d, J = 8.6 Hz, 2H), 6.67 (d, J = 8.7 Hz, 2H), 3.91 (t, J = 6.5 Hz, 2H), 2.55 (q, J = 7.4 Hz, 2H), 1.77 (dd, J = 13.7, 6.4 Hz, 2H), 1.69 – 1.61 (m, 2H), 1.42 – 1.29 (m, 12H). ^13^C NMR (101 MHz, CDCl_3_) δ 157.97, 143.92, 143.90, 143.87, 143.32, 143.24, 143.18, 142.85, 141.20, 141.18, 140.71, 140.56, 139.37, 138.42, 138.37, 138.30, 138.21, 136.08, 132.77, 132.01, 131.89, 131.55, 131.48, 127.90, 127.79, 127.77, 126.60, 126.54, 126.09, 126.03, 113.84, 67.96, 34.17, 29.62, 29.58, 29.53, 29.47, 29.20, 28.50, 26.21, 24.78. TOF-MS m/z calc for C_114_H_94_OS [M+1]^+^ 1511.7104, found 151.7177.

**Spectroscopy**

**Near edge X-ray absorption fine structure spectroscopy.**

Supplementary Fig. 72 shows the angular dependent NEXAFS data at C *K*-edge for SAMs of Au-SC_10_-O-TPE at different bending radii. The lowest resonance peaks are due to the transitions from C 1s to π*_C=C_ orbitals (at ~286.2 eV). Here we hypothesize that the vector π* of the four phenyls is canceled due to their symmetry. Thus, the total vectors of π* orbitals of TPE is parallel to the middle C=C bond in Supplementary Fig. 72c, which connect the four phenyls. The peak at ~288.4 eV is ascribed to C-H orbitals. The broader signals at higher photon energy are assigned to *σ** transitions. Assuming a random azimuthal orientation between molecular plane and substrate, the π* resonances intensity (I*) ratio at 90^o^ and 40^o^ incident angles (*θ*) can be expressed as follows:

$\frac{I_{\pi^{*}}\text{(}\text{α}\text{, }\text{90°)}}{I_{\pi^{*}}\text{(}\text{α}\text{, }\text{40°)}}\text{=}\frac{{\text{P}\text{(sin}}^{\text{2}}\text{α}\text{sin}^{\text{2}}\text{90°+2}\text{cos}^{\text{2}}\text{α}\text{cos}^{\text{2}}\text{90°)+(1-}\text{P}\text{)}\text{sin}^{\text{2}}\text{α}}{{\text{P}\text{(sin}}^{\text{2}}\text{α}\text{sin}^{\text{2}}\text{40°+2}\text{cos}^{\text{2}}\text{α}\text{cos}^{\text{2}}\text{40°)+(1-}\text{P}\text{)}\text{sin}^{\text{2}}\text{α}}$ (1)

in which *P* = 0.90 is the linear polarisation factor, and *α* is the average tilt angle of the middle C=C

bond long axis with respect to the substrate surface normal^9, 10^.

**X-ray and ultraviolet photoelectron spectroscopy.**

We recorded UPS spectrum to determine the HOMO levels (*E*_HOMO_) and work function. HOMO level is calculated from the *E*_cut off_ of the secondary electron peak and HOMO-onset (*E*_HOMO-onset_). Supplementary Fig. 72a and b shows the secondary electron peak and HOMO onset edge of the UPS spectra. The detailed analysis procedure has been reported before ^11^:

*E*_HOMO_ = *hv*- (*E*_cut off_ –*E*_HOMO- onset_) (2)

where *hv* is the incident photon energy 21.22 eV. We determined the values of *E*_cut off_ from the intercept between the linear extrapolation of the baseline and linear extrapolation of the secondary electron peak (Supplementary Fig. 72a). Similarly, the values of *E*_HOMO-onset_ were determined from the intercept between the linear extrapolation of the baseline and linear extrapolation of the lower binding energy side of HOMO onset peak (Supplementary Fig. 72b).

Work function (WF) is also calculated from the UPS spectrum and by the following equation:

WF = *hv*- (*E*_cut off_ –*E*_Fermi_ ) (3)

where *hv* is also the incident photon energy 21.22 eV. All UPS spectra were referenced to the Fermi edge of a clean Au surface and therefore the values of *E*_Fermi_ is 0 eV.

Next, we can also estimate the LUMO energy level (*E*_LUMO_) by using the values of the optical HOMO-LUMO gap obtained from the emission spectra (Fig. 4a and Supplementary Table 1) subtract the values of *E*_HOMO_. The values of *E*_LUMO_ are listed in the Supplementary Table 1.

Supplementary Fig. 73-74 shows the XPS spectrum of Au-SC_10_ and Au-SC_10_-O-TPE SAMs at different convex and concave bending radius. All the Au singnal (Supplementary Fig. 73b, f, j and Supplementary Fig. 74b, f, j) include two peaks of Au 4f_5/2_ and Au *4f_7/2_*. The carbon signal can be fitted to servel peaks. From low to high binding energy, these C1s peaks are corresponding to phenyl (centered at ~284.0 eV ), C-C (centered at ~284.4 eV ), C=C (centered at ~285.2 eV ) and C-O (centered at ~286.0 eV ), respectively. The weak peaks centered at ~ 291.0 eV is attributed to π-π* satellite.

All the sulfur signals (Supplementary Fig 73d, h, l and 74d, h, l) are assigned to *S2p_1/_*_2_ and *S2p_3/2_*. We fitted the *2p* spin-orbit doublet with a splitting of 1.18 eV and an area ratio of S *2p_3/2_*:*2p_1/2_* = 2:1. The *2p_3/2_* and *2p_1/2_* peaks are centered at ~161.8 eV (red line) and at ~163.1 eV (green line). The spectra indicate formatioin of dense, well packed Au-SC_10_-O-TPE SAMs on the gold electrode.

We calculated the surface coverage from the sulfur signal using eq. 4.

*I_0,S_* = *I_s_* / exp (*-d / λ*) (4)

Here, *I_0,S_* is the initial signal generated from the 2p orbital of S atoms without attenuation over the thickness of SAMs. The reported theoretical value of *Γ_SAM_* of Au-SC_10_ SAMs on Au (111)^12^ surface is 9 × 10^-10^ mol/cm^2^. The X-ray photoelectron spectrum of Au-SC_10_ is shown in Supplementary Fig. 39a-d. Thus, we compared the values of *I_0,S_* of the SAMs formed on Au substrate surfaces against the value *I_0,S_* of SC_10_ SAMs (Supplementary Fig. 73a-d) formed on Au substrate to estimate the relative *Γ_SAM_* values of the SAMs in this study. The estimated *Γ_SAM_* of Au-SC_10_-O-TPE is 5.9 × 10^-10^ mol/cm^2^.

**Simulation and calculation**

To create the curved SAM models, the length *l_x_* of the gold substrate along the bending axis, here the x-axis, is divided by the chosen angle *θ*. This gives the base radius of curvature *r_c_* in eq. 5:

$r_{c}=\frac{l_{x}}{\theta}$ (5)

The system is shifted so that the z-position of the botto layer of gold atoms is set at zero, and the middle of the xy plane coincides with the center of the gold slab. For each atom $i$ in the model, the effective radius of curvature *r_i_* is defined as follows in eq 6:

$r_{i}=r_{c}+z_{i}$ (6)

The angle formed between the atom and the center of curvature is:

$\theta_{i}=\frac{x_{i}}{r_{i}}$ (7)

The new position of the atom is then as eq. 8:

$\left\{ \begin{matrix} x_{i}^{'}=r_{i}\sin\theta_{i} \\ z_{i}^{'}=r_{i}\cos\theta_{i}-r_{c} \end{matrix} \right.$ (8)

Supplementary Fig. 76 shows the curved systems, both convex and concave, for three angles of curvature: 5, 10, 15°, and Table S3 shows the corresponding radius of curvature for each model.

Supplementary Fig. 75 shows computed heat maps of phenyl rings relative orientation with respect to their separation. For all systems except the most concave with +15° curvature, the pair distribution is relatively diffuse with a large hill centered around an orientation angle of 90° (loose T-stack) that extends to large distance demonstrating the large-scale correlation of the SAM structure. The most concave model shows a higher level of structuration with high density at short distance with peaks showing a marked tendency towards 60° and 120° for the relative orientation. This predicts that the most concave SAM geometry forces the molecules to depart from the weak, sparsely populated T-stack structure to favor more extensive parallel stacking that was prevented by steric constraints prior to the mechanically-stimulated supramolecular crowding in the most aggregated junction. The MD models confirm that concave curvature is therefore able to induce a well-defined and large-scale ordering of the SAM (see main text Fig. 5b, and also reduced atomic fluctuations in the most concave c15 model in Supplementary Fig. 76 above) that maximizes π-π stacking by increasing the TPE head groups proximity, leading to an improvement in the supramolecular interaction (main text Fig. 5c) in good agreement with the red-shifting of the UV-vis spectrum in the DFT calculations (main text Fig. 5d) and in good agreement with the experimental materials characterization and measured device properties (main text Fig. 1-4 and Supplementary Information and analysis above).

Supplementary Fig. 79 shows the results of the transport calculations performed in dark conditions (no photoexcitation) on dimers obtained from the convex (Supplementary Fig. 79a) and concave (Supplementary Fig. 79b) MD models (Supplementary Fig. 76). The calculated larger current (Supplementary Fig. 79c) in the concave assembly reflects the lowering of the acceptor levels (DOS in Supplementary Fig. 79d) consistent with the computed (Fig. 5d) and measured (Fig. 4b) UV-Vis spectra. This places the low-lying empty levels close to the Fermi level, for easy population during photoexcitation, as reflected also in the computed transmission function (Supplementary Fig. 79e). Populating the lowest unoccupied orbitals close to the Fermi level promotes the transmission and explains the current increase measured experimentally for the concave system during photoexcitation.

**Supplementary References**

1 Jia, C. *et al.* Covalently bonded single-molecule junctions with stable and reversible photoswitched conductivity. *Science* **352**, 1443-1445 (2016).

2 Pourhossein, P., Vijayaraghavan, R. K., Meskers, S. C. J. & Chiechi, R. C. Optical modulation of nano-gap tunnelling junctions comprising self-assembled monolayers of hemicyanine dyes. *Nat. Commun.* **7**, 11749 (2016).

3 Xu, B. *et al.* High-performance two-photon absorption luminophores: large action cross sections, free from fluorescence quenching and tunable emission of efficient non-doped organic light-emitting diodes. *J. Mater. Chem. C* **2**, 3416-3428 (2014).

4 Cao, T., Li, D., Yao, X., Xu, Y. & Ma, X. Preparation and properties of organo-soluble tetraphenylethylene monolayer-protected gold nanorods. *Dyes Pigm.* **124**, 1-5 (2016).

5 Yuan, L. *et al.* Transition from direct to inverted charge transport Marcus regions in molecular junctions via molecular orbital gating. *Nat. Nanotechnol.* **13**, 322-329 (2018).

6 Chua, M. H., Zhou, H., Lin, T. T., Wu, J. & Xu, J. W. Aggregation-induced emission active 3,6-bis(1,2,2-triphenylvinyl)carbazole and bis(4-(1,2,2-triphenylvinyl)phenyl)amine-based poly(acrylates) for explosive detection. *J. Polym. Sci. A Polym. Chem.* **55**, 672-681 (2017).

7 Lin, J.-L. et al. Molecular diodes with tunable threshold voltage based on π-extended tetrathiafulvalene. *Adv. Mater. Interfaces* **9**, 2201238 (2022).

8 Cao, F., Duan, H., Li, Q. & Cao, L. A tetraphenylethene-based hexacationic molecular cage with an open cavity. *Chem Commun* **58**, 13389-13392 (2022).

9 Nerngchamnong, N. *et al.* The role of van der Waals forces in the performance of molecular diodes. *Nat. Nanotechnol.* **8**, 113-118 (2013).

10 Yuan, L. *et al.* Controlling the direction of rectification in a molecular diode. *Nat. Commun.* **6**, 6324 (2015).

11 Qi, Y. *et al.* Filled and empty states of alkanethiol monolayer on Au (111): Fermi level asymmetry and implications for electron transport. *Chem. Phys. Lett.* **511**, 344-347 (2011).

12 Yuan, L., Jiang, L. & Nijhuis, C. A. The drive force of electrical breakdown of large-area molecular tunnel junctions. *Adv. Funct. Mater.* **28**, 1801710 (2018).
